# Nonenzymatic Detection of SARS-CoV‑2 RNA Using DNA Nanoswitches

**DOI:** 10.1021/acsnanomed.6c00053

**Published:** 2026-07-10

**Authors:** Javier Vilcapoma, Asmer Aliyeva, Andrew Hayden, Arun Richard Chandrasekaran, Lifeng Zhou, Jibin Abraham Punnoose, Darren Yang, Clinton H. Hansen, Simon Chi-Chin Shiu, Alexis Russell, Kirsten St. George, Wesley P. Wong, Ken Halvorsen

**Affiliations:** † The RNA Institute, University at Albany, State University of New York, Albany, New York 12222, United States; ‡ Department of Nanoscale Science and Engineering, University at Albany, State University of New York, Albany, New York 12222, United States; § Program in Cellular and Molecular Medicine, Boston Children’s Hospital, Boston, Massachusetts 02115, United States; ∥ Wyss Institute for Biologically Inspired Engineering, 1812 Harvard University, Boston, Massachusetts 02115, United States; ⊥ Department of Biological Chemistry and Molecular Pharmacology, Blavatnik Institute, Harvard Medical School, Boston, Massachusetts 02115, United States; # Laboratory of Viral Diseases, Wadsworth Center, New York State Department of Health, Albany, New York 12208, United States; ∇ Department of Biomedical Science, University at Albany, State University of New York, Albany, New York 12222, United States

**Keywords:** DNA nanoswitches, biosensing, DNA nanotechnology, SARS-CoV-2, diagnostics, viral RNA detection

## Abstract

The emergence of a highly contagious coronavirus in 2019 led to an unprecedented need for large-scale diagnostic testing. The associated challenges, including reagent shortages, cost, deployment delays, and turnaround time, have all highlighted the need for an alternative suite of low-cost tests. Here, we demonstrate a test for SARS-CoV-2 RNA that provides direct detection of viral RNA and eliminates the need for costly enzymes. We employ DNA nanoswitches that respond to segments of the viral RNA by a change in shape that can be read by gel electrophoresis. A multitargeting approach samples 120 different viral regions to improve the limit of detection and provide robust detection of viral variants. We applied our approach to a cohort of clinical samples, positively identifying a subset of samples with high viral loads. Since our method directly detects multiple regions of viral RNA without amplification, it eliminates the risk of amplicon contamination and renders the method less susceptible to false positives. This tool can benefit diagnostic options for COVID-19 and future emerging outbreaks, providing a third option between amplification-based RNA detection and protein antigen detection. Ultimately, we believe that this tool can be adapted both for low-resource onsite testing as well as for monitoring viral loads in recovering patients.

## Graphical abstract

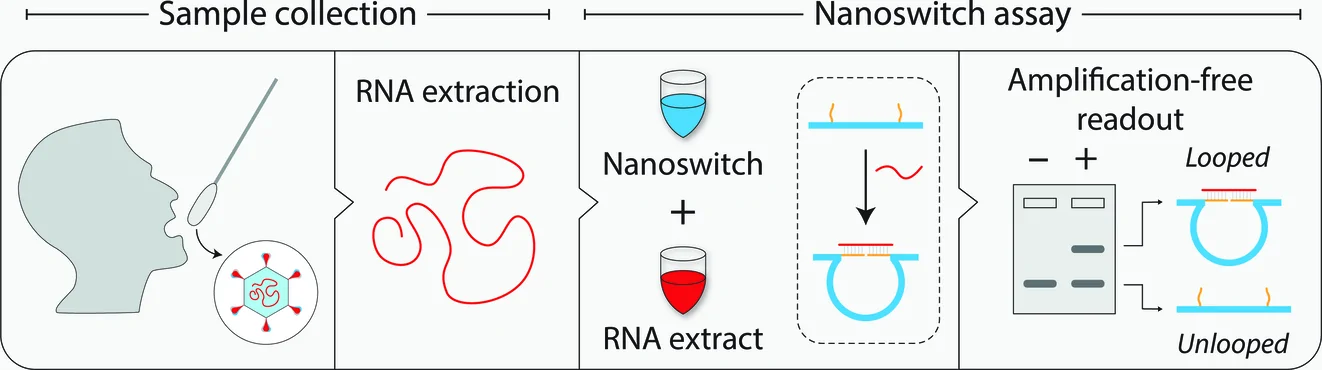

## Introduction

A novel coronavirus, SARS-CoV-2 (severe acute respiratory syndrome coronavirus 2), caused an outbreak of the disease COVID-19,^1,2^ with over 600 million reported cases and 6.7 million deaths globally in the first three years of the pandemic.^3^ The pandemic has highlighted the need for the development of rapid and low-cost alternate detection strategies for large-scale and frequent testing.^4,5^ The gold-standard method for viral detection in clinical samples is RT-qPCR (reverse transcription quantitative polymerase chain reaction), which involves nucleic acid amplification and its monitoring via fluorescently labeled probes. The tests can be performed in a couple of hours, but logistical issues and the need for specialized laboratories make typical turnaround times several days, creating a gap in preventing COVID positive individuals from transmitting the virus before the results are obtained. Antigen tests can be used to detect the various proteins of SARS-CoV-2 virions using lateral flow assay technology.^5^ These tests have been developed and marketed as rapid home-based tests and are now widely used. They have less sensitivity than RT-qPCR tests and are commonly negative for 2–3 days following onset of symptoms but provide results quicker and with less expense. At various points in the pandemic, large-scale global testing strained supply chains and created backorders of reagents and consumables required for RT-qPCR assays with consequent bottlenecks in testing^6,7^ or general scarcity of rapid antigen tests. These limitations in testing have negatively affected our ability to control the pandemic. Further, in the acute phase of the pandemic, the frequency and speed of testing were found to be more practically relevant than test sensitivity for large-scale COVID-19 testing.^8^ Thus, the focus of new diagnostic assays shifted from solely high sensitivity to also include aspects of cost, turnaround time, and operation outside a lab by nonspecialists.^9^ These alternate testing strategies provide rapid results that aid in quick diagnosis and thus control of disease spread in contrast to highly sensitive tests with longer turnaround times.

The challenges faced by traditional in vitro diagnostic techniques and the need for more frequent testing have accelerated the development of alternative COVID-19 testing strategies, with many new tests being developed.^4,5,10,11^ Many of the more conventional tests rely on RT-qPCR^12,13^ for viral RNA or antigen testing for viral proteins.^14^ New methods are increasingly adopting alternate approaches such as CRISPR,^15–18^ isothermal amplification methods^19,20^ including loop-mediated isothermal amplification (LAMP),^21,22^ and nanotechnology-enabled variations to enhance performance.^10,23,24^ Nanotechnology-based approaches for detecting SARS-CoV-2 include those based on gold nanoparticles,^25–28^ molecular switches,^29^ carbon nanotubes,^30^ graphene,^31,32^ and quantum dots.^33–35^ Methods are also advancing with detection technologies including the innovative use of lateral flow,^36^ microfluidics,^37^ color changes,^18^ electrochemical readout,^38,39^ and spectroscopic readouts.^40–42^ With few exceptions, these approaches rely on enzymatic amplification for detection or the use of proteins and antibodies for signal generation, which add cost and logistical challenges for transportation, storage, and use. To address some of these challenges, a few methods have been developed for nonenzymatic detection of viral RNA.^43,44^ One promising approach for nonenzymatic detection of viral RNAs is with DNA nanotechnology, which allows for construction and dynamic control of nanoscale objects built from DNA.^45,46^ Precise spatial positioning and programmable control over the shape of DNA nanostructures have been used extensively for many applications including biosensing and drug delivery.^47^ For applications to viruses, DNA nanostructures have been employed to elucidate viral vaccine design principles^48^ and in viral diagnostics^49^ and therapeutics.^50^

In our own work, we previously established proof of concept for detection of Zika virus RNA.^51^ There we established some design parameters for designing DNA nanoswitches to detect viral RNA, but applicability remained limited by overnight incubation, preamplification of viral RNA, and lack of validation with clinical samples. Here, we considerably expand on our prior work to develop a DNA nanoswitch-based assay for nonenzymatic, direct detection of SARS-CoV-2 from clinical samples. We optimized DNA nanoswitch design and reaction conditions to enable detection in under 2 h and as short as a few minutes. We developed a novel DNA nanoswitch design for simultaneous targeting of multiple (up to 120) fragments of the SARS-CoV-2 genome, improving our detection limit by nearly 2 orders of magnitude. We optimized processing conditions for viral RNA and ultimately applied the methodology to the detection of SARS-CoV-2 from clinical samples with visual identification of 10/10 negative samples and 14/24 positive samples.

## Results

### DNA Nanoswitch Design and Operation

The DNA nanoswitch is constructed based on DNA origami principles. Typically, in DNA origami, a long single stranded DNA is folded into specific two- or three-dimensional shapes using short complementary strands.^52^ Here, we create a simple linear duplex (the “off” state) using a 7249-nt long scaffold strand (commercially available M13 bacteriophage viral genome) tiled with short complementary backbone oligonucleotides^53,54^ (Figure S1). Two of the oligonucleotides contain single stranded extensions (detectors) that are complementary to parts of a target nucleic acid (Figure 1a). On binding the target sequence, the nanoswitch is reconfigured from the linear “off” state to a looped “on” state.^55^ This conformational change is easily read out on an agarose gel where the on and off states of the nanoswitch migrate differently due to the topological difference (Figure 1a, inset). Importantly, this approach requires no complex equipment or enzymatic amplification. The signal comes from the intercalation of thousands of dye molecules from regularly used DNA gel stains (GelRed in this case), providing a high detection signal due to the length of the nanoswitch. In previous work, we used similar DNA nanoswitches for detection of microRNAs,^56,57^ viral RNAs,^51^ antigens,^58^ and enzymes.^59^

**Figure 1:**
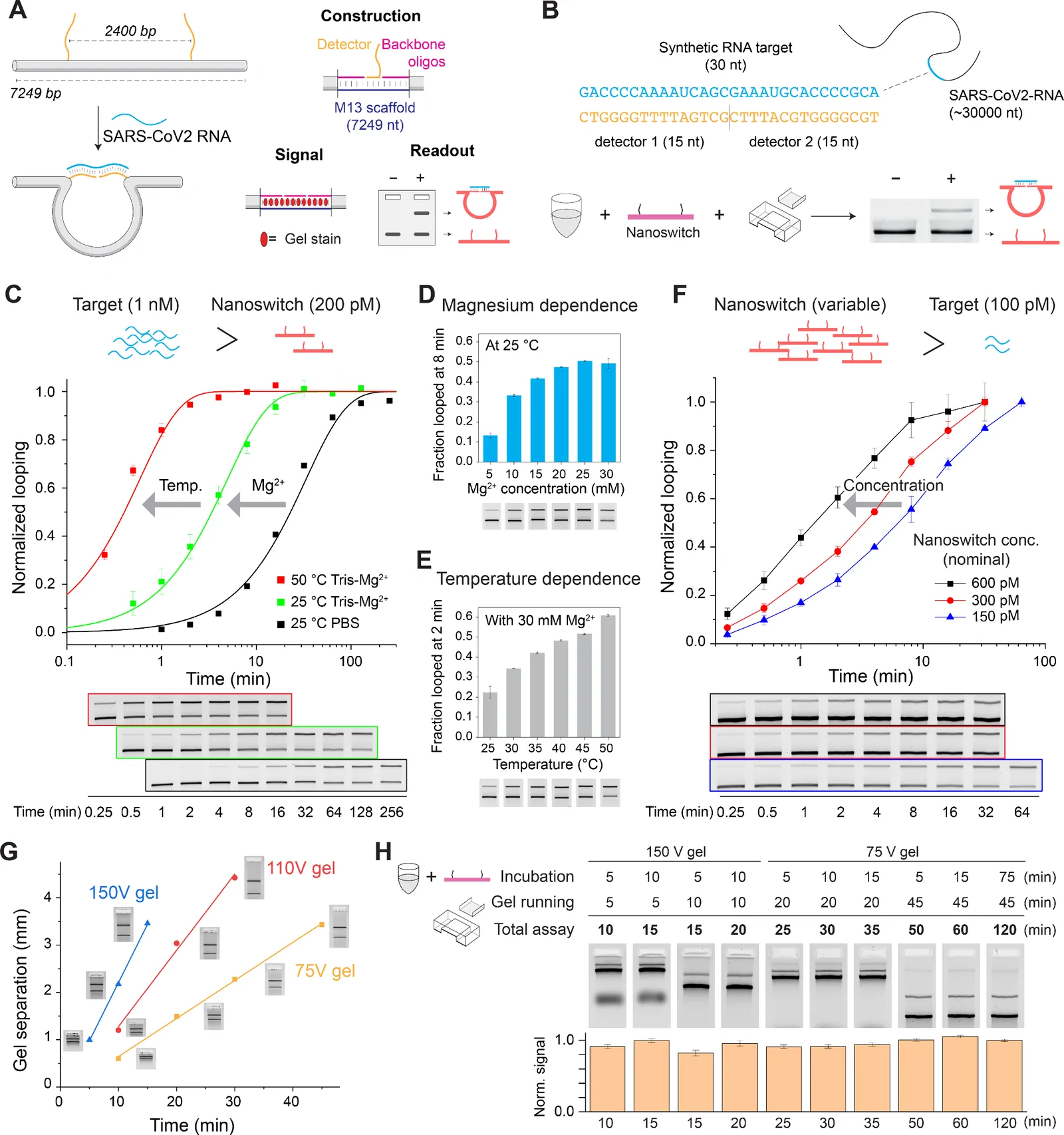
DNA nanoswitch detection of SARS-CoV-2 RNA. (A) The DNA nanoswitch is a self-assembled DNA construct designed to form a loop upon interacting with a specific target sequence. The nanoswitch is stained with intercalating dye and imaged on a gel for detection readout. (B) Design and validation of a DNA nanoswitch targeting a 30 nt portion of the N-gene. (C) Reaction kinetics for a 30 nt RNA target in excess shows nearly 2 orders of magnitude improvement with optimal magnesium and temperature. (D) Effect of various magnesium concentrations on room temperature kinetics. (E) Effect of various temperatures on kinetics. (F) Reaction kinetics with limited target. (G) Gel separation of nanoswitch with different times and voltages. (H) Overall assay time for a 50 pM target. Error bars represent standard deviation from three technical replicates.

In this work, we considerably expand on our previous knowledge to accomplish direct, nonenzymatic detection of SARS-CoV-2 viral RNA. As a first step, we designed a nanoswitch with detectors that respond to a target sequence in SARS-CoV-2 that was expanded from one of the original CDC N2 primer targets.^60^ Using a synthetic RNA target, we confirmed that our nanoswitches could detect this SARS-CoV-2 fragment (Figure 1b). Next, we moved to address one of the key challenges in detection of SARS-CoV-2 RNA: achieving a short detection time for a low concentration target. Previous nanoswitch work with proteins had shown rapid detection with a 30 min incubation,^58^ but this goal had not yet been achieved for nucleic acids, where secondary structures can slow reaction kinetics. To address this issue, we screened the kinetics of the nanoswitch assay for a variety of conditions using a 30 nt synthetic RNA target that corresponds to a region in the SARS-CoV-2 genome nucleocapsid (N) gene (Figure 1c). First, we performed kinetic experiments at room temperature in phosphate buffered saline (PBS), which took nearly 2 h to reach completion (Figure 1c). We then considered the addition of magnesium, which is known to facilitate nucleic acid hybridization.^61,62^ Using a Tris-HCl buffer, we tested different concentrations of magnesium. We found that achieving the highest detection signal required at least 10 mM MgCl_2_ and that kinetics were enhanced up to 30 mM MgCl_2_ (Figure 1d and Figure S2). Choosing 30 mM MgCl_2_, we then measured kinetics at different temperatures, which showed a continued increase until ∼50 °C (Figure 1e and Figure S3). With a 1 nM target RNA concentration, the magnesium and elevated temperature enabled complete reactions in less than 2 min, nearly 2 orders of magnitude faster than our starting condition (Figure 1c).

In a typical viral detection assay, we expect the target concentration (∼fM level) to generally be lower than the nanoswitch concentration (∼200–500 pM level). To assess more realistic conditions, we evaluated the kinetics for reactions with excess nanoswitch (Figure 1f). First, we confirmed that the rate of the signal accumulation relative to maximum signal is independent of the target concentration under these conditions (Figure S4). Next, we evaluated the kinetics of the reactions for 100 pM target RNA with varying nanoswitch concentrations. As expected, we found that increasing the nanoswitch concentration increased the reaction rate. At the highest nanoswitch concentration tested (∼600 pM), it took less than 2 min to reach half signal and less than 10 min to reach 90% signal (Figure 1f). To ensure a short end-to-end assay time, we additionally optimized the gel running conditions. We maximized band separation at a given voltage by minimizing buffer height above the gel (Figure S5), which nearly doubled the separation. We then imaged looped nanoswitches at different voltages and running times, reducing the gel running time from 45 min to 5–20 min with minimal signal loss (Figure 1g). These optimizations enabled detection of a 50 pM RNA target with an end-to-end assay time of as little as 10 min without sacrificing more than 20% of the signal (Figure 1h).

### Targeting Multiple Viral RNA Fragments for Signal Enhancement

Next, we turned our attention to improving the analytical sensitivity of the assay to be suitable for viral targets. For SARS-CoV-2, the viral load can vary widely among patients from ∼10^3^ to 10^11^ copies/mL^63,64^ due to various factors including viral variant, patient profile (age, health, vaccination status, etc.), and time since infection.^65^ Viral load is often considered a proxy for infectiousness, with most infectious individuals having excess of 10^6^ copies/mL and one study^63^ reporting a median viral load as 10^6.8^ copies/mL (roughly 10 fM). Our previous sensitivity for small RNA targets such as microRNA has been reported in the ∼100 fM range. To improve sensitivity, we developed a new strategy for simultaneous targeting of different fragments of viral RNA. The concept is based on the idea that fragmenting the viral RNA produces many discrete targets from a single viral RNA genome (Figure 2a). A typical assay with a single target sequence can capture one target RNA per viral RNA, but an assay with multiple target sequences can capture multiple target RNAs per viral RNA to increase signal. In a nanoswitch assay, this can be accomplished by targeting several different SARS-CoV-2 sequences with identical loop sizes to essentially add the signal from multiple targets (Figure 2b). To achieve this, we developed a new nanoswitch design where multiple detectors that target distinct regions of the viral genome can be integrated onto a single nanoswitch, improving on previous ideas from combining individual single-target nanoswitches.^51^

**Figure 2:**
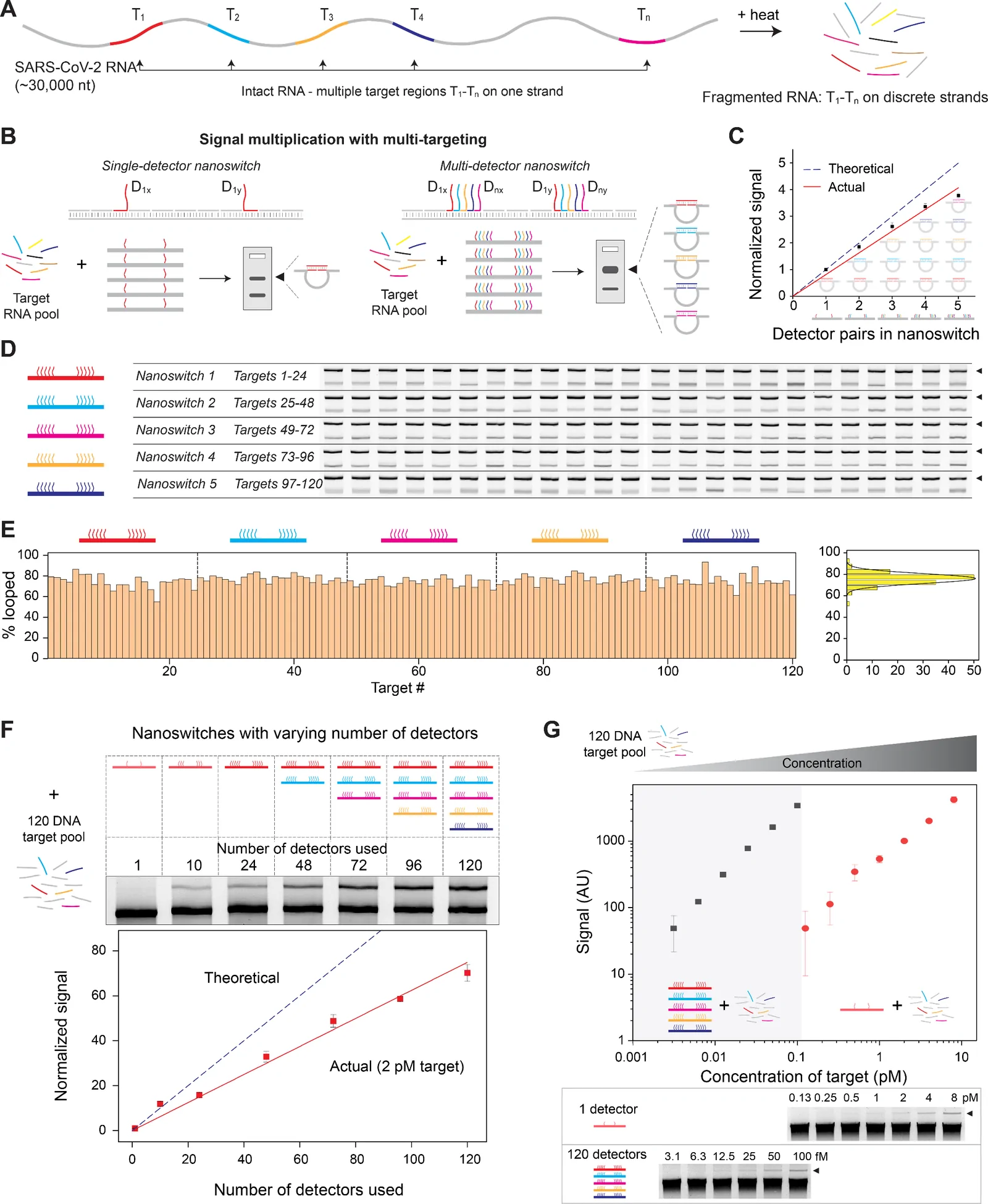
Improving sensitivity with multidetector nanoswitches. (A) Long viral RNA can have many target regions that separate into discrete strands after fragmentation. (B) Concept of single-detector and multidetector sensing. (C) Validation of multidetector sensing concept by targeting one to five sequences in a five-sequence pool. (D) Development and validation of five 24 detector nanoswitches to enable targeting of 120 different regions. Gels demonstrate detection of each individual target. (E) Quantified looped % of each nanoswitch target, with a histogram showing the distribution. (F) Detection of a mock viral RNA using 120 equimolar targets and corresponding signal increase with increased detectors. (G) Overall sensitivity of the 120 detector nanoswitch approach compared with single detector. Error bars represent standard deviation from three technical replicates.

The detection signal for the nanoswitch assay is based on the looping of the nanoswitch when a target RNA binds a pair of detectors on the nanoswitch. To design a multidetector nanoswitch, we incorporated pairwise detectors on the nanoswitch, with each pair offset along the length of the nanoswitch. We designed these pairwise detectors to be a fixed distance apart so that they all form the same loop size on binding their specific target (Figure S1). Under typical conditions for SARS-CoV-2 detection (when nanoswitch concentration ≫ target concentration), this results in the ability to detect multiple SARS-CoV-2 fragments with an additive signal (Figure 2b). To implement this concept, we first identified loops that could be repositioned along the length of the nanoswitch with similar gel migrations. We screened different loop sizes and positions and found that a nearly centered loop of ∼2400 bp provided a constant signal when shifted (Figure S6). The identified design provided two 720 nt regions to place detector pairs. As a first proof of concept, we designed and built a five-target nanoswitch to recognize five 30 nt regions of the SARS-CoV-2 genome. We confirmed that the nanoswitch responded to each of the five targets individually with an indistinguishable signal (Figure S7). We also verified that the kinetics of binding a single RNA target is identical between a multidetector nanoswitch and a single target nanoswitch as expected (Figure S8). To demonstrate the signal multiplication concept, we used an equimolar pool of the 5 RNA targets representing a perfectly fragmented viral RNA and tested them against 5 different nanoswitches with different numbers of detectors that can target from 1 to all 5 of the targets. We show that for a constant target pool, we achieved a nearly stoichiometric increase in the signal as we increase the targeting capability (Figure 2c).

Having proven the concept, we sought to expand the number of targets. Considering the long length of viral RNAs such as SARS-CoV-2 (∼30,000 nt) and the short length required for detection (∼20–60 nt), this idea can in principle be expanded to hundreds of targets and achieve a corresponding level of signal amplification. We divided the 720 nt nanoswitch regions into 30 nt segments to enable development of 24-target nanoswitches. We designed and built five such 24-target nanoswitches to provide detection of up to 120 fragments that span the SARS-CoV-2 viral genome. We confirmed the positive detection of each of the 120 individual targets using DNA oligonucleotides (Figure 2d) and showed that maximum detection efficiency appears to be a Gaussian distribution with a mean 76% (Figure 2e). To measure the signal enhancement, we used an equimolar pool of the 120 targets and measured the detection signal using single nanoswitches with 1, 10, or 24 detector pairs and mixtures of 1, 2, 3, 4, and 5 24-target nanoswitches. We found that the signal increases nearly linearly with the number of detectors in the mixture (Figure 2f). Using serial dilutions of the 120-target mixture, we tested the sensitivity of the assay with the five multidetector nanoswitch mixture and a single target nanoswitch. Compared to the LoD of 122 fM or 0.76 amol for a single target sequence, our signal multiplication strategy provides a ∼65-fold improvement to 1.9 fM or 0.01 amol, corresponding to ∼6000 genome copies (Figure 2g).

### Testing and Validation with Transcribed Viral RNA

Key to our signal enhancement strategy is the ability to robustly fragment the long viral RNA. To develop and test fragmentation protocols, we first made and purified an in vitro transcribed (IVT) RNA of the SARS-CoV-2 N-gene (1279 nt). We tested three fragmentation reagents, a commercial buffer and two homemade buffers and settled on a homemade Tris-Magnesium reagent with 3 mM MgCl_2_ and pH 8.5 (Figure S9). Using our homemade fragmentation buffer, we fragmented the N-gene RNA for various lengths of time from 1 min to ∼1 h. We ran the products on a 2% agarose gel (Figure 3b) and estimated fragment sizes with a reference ladder. We found mean fragment sizes decreasing from ∼800 nt for 1 min to ∼50 nt for 64 min with distribution widths also decreasing with fragmentation time (Figure 3c).

**Figure 3:**
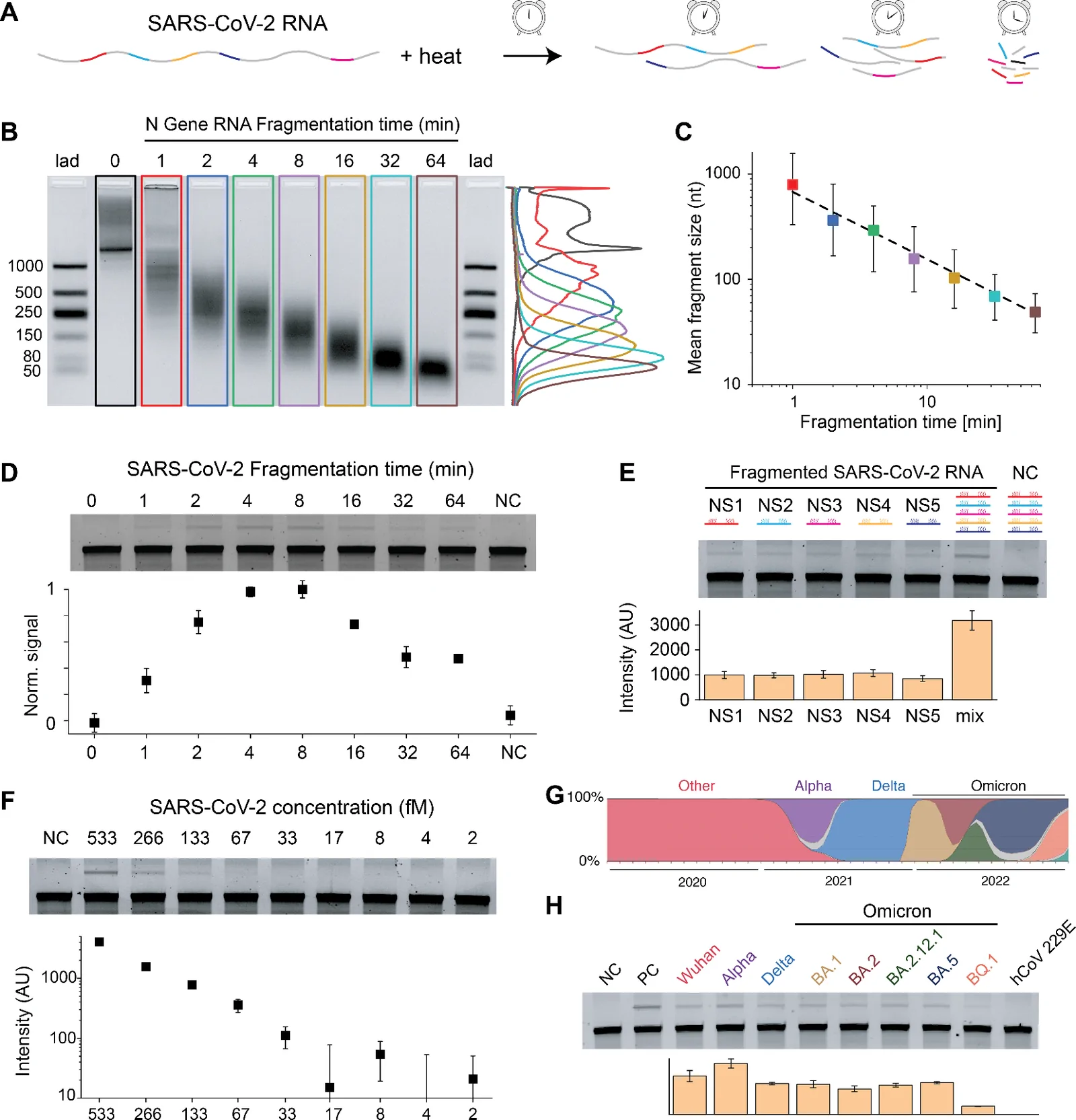
Detection of SARS-CoV-2 RNA. (A) Scheme for SARS-CoV-2 fragmentation. (B) In vitro transcribed N-gene RNA is fragmented using heat for various times with progressively smaller fragments over time. (C) Distribution of apparent fragment sizes as a function of fragmentation time. (D) Multidetector nanoswitch detection of fragmented SARS-CoV-2 full genome RNA controls show optimal performance in the 2–16 min range. (E) Detection of full genome SARS-CoV-2 at 533 fM concentration with each of five 24 detector nanoswitches and the mixture of all five. (F) Analytical sensitivity of the 120 multidetector assay against full genome SARS-CoV-2 RNA (4 fM data point below *x*-axis). (G) Prominence of different variants during the first 3 years of the pandemic in the United States (data from GISAID). (H) Detection of major variants, and no detection of a non-SARS human coronavirus hCoV 229E. Error bars represent standard deviation from three technical replicates.

Next, we optimized the nanoswitch design for the RNA fragments. Long RNAs, including viral RNAs, are known to contain strong secondary structures, some of which may persist even as the RNA is fragmented into smaller pieces in the 200 nt range.^66,67^ Such structured regions could slow or otherwise impede binding to the DNA nanoswitch. During this project, evidence in our lab emerged that longer detector regions enhanced capture of a 401 nt RNA.^68^ To see if that result applied to our fragmented viral RNA, we made and tested nanoswitches with detection regions of 15, 20, 25, and 30 nt and found that overall detection signal clearly increased with longer detector lengths (Figure S10). This result was substantial enough to warrant redesign and reconstruction of our five 24 detector nanoswitches with the 30 nt detector pairs. We redesigned our target regions and detectors for the new nanoswitches, which we again validated for each target (Figure S11). One unintended consequence of this change was false-positive detection, likely due to weak cross-reactivity of detectors. These false positives were eliminated by poststaining our gels rather than adding the stain to the samples, so we altered our protocol accordingly.

Having optimized our multitargeting nanoswitches for fragmented N-gene RNA, we expanded the IVT detection to a more realistic target RNA, spanning the full SARS-CoV-2 genome across six strands. We fragmented the IVT RNA and found an optimal fragmentation time of 4–8 min based on detection of our five 24 detector nanoswitch mixture, balancing the quality of signal with our time requirements for a short test (Figure 3d). This result suggests that mean fragment sizes in the range of ∼150–300 nt provide the most efficient detection. Choosing a fragmentation time of 4 min, we were able to detect the SARS-CoV-2 RNA with each of our five multidetector nanoswitches in roughly equal proportion, with over a 3-fold increase when they were combined (Figure 3e). This result confirms good genome coverage for targeting different regions as well as a robust signal multiplication effect. Using this mixture of five 24-detector nanoswitches, we were able to achieve an LOD for this full SARS-CoV-2 genome equivalent of 33 fM or about 10^5^ copies (Figure 3f).

### Detection of Viral RNA from SARS-CoV-2 Variants

Throughout the pandemic, we were challenged with an evolving virus. Several dominant variants have emerged and were later replaced by others. In some cases, mutations in these variants have affected the reliability of diagnostic tests.^69,70^ To test the robustness of our nanoswitch assay, we obtained full genome RNA controls for the original (Wuhan) strain and each of the 6 other variants that have been dominant in the US during the first 3 years of the pandemic according to data from GISAID^71^ (Figure 3g). We additionally obtained a non-SARS human coronavirus (hCoV229E) to test cross-reactivity. Since our nanoswitch-based assay covers the entire SARS-CoV-2 genome, our assay is uniquely indiscriminate to viral variants with different mutations. To demonstrate this, we used the five 24-detector nanoswitch mixtures with fragmented RNA from the original strain as well as the six variants and the negative control (Figure 3h). We found that our nanoswitch assay was able to similarly detect all SARS-CoV-2 variants but exhibited no reactivity with hCoV229E. We believe that intensity variations in some variants were due to differing concentrations in the source material, which was supported by RT-qPCR data on the same material that showed nearly a 4-fold variation across samples, with BQ.1 having the lowest concentration (Figure S12). Our unique approach of wide genome coverage makes our system robust against new variants.

### Detection of SARS-CoV-2 from Clinical Samples

To test our assay on clinical samples, we obtained 23 positive and 12 negative samples of RNA extracts from nasal or nasopharyngeal swabs (previously confirmed by RT-qPCR). Our optimized assay is suitable for use with RNA extraction protocols typically used for validation by RT-qPCR, with only an additional fragmentation step. We used 2 μL of RNA extract and fragmented the sample for 8 min, followed by incubation with the five 24-detector nanoswitch mixture. Our assay was able to confirm all 12 negatives and registered positive on 14 out of the 23 samples (Figure 4 and Figure S13). The positive hits were largely correlated with the Ct values obtained from RT-qPCR, indicating a Ct threshold for our current assay in the mid to low 20s. Interestingly, Ct values did not always correlate with the nanoswitch readout. As one example of many, samples 14 and 15 had Ct values of 21.4 and 18.4, respectively (Figure S13). This would suggest that sample 15 should have ∼8× more SARS-CoV-2 RNA, and yet we clearly observe ∼2× less signal from our measurement. The cause of such discrepancies may have to be resolved with further investigation before progressing as a clinical tool.

**Figure 4:**
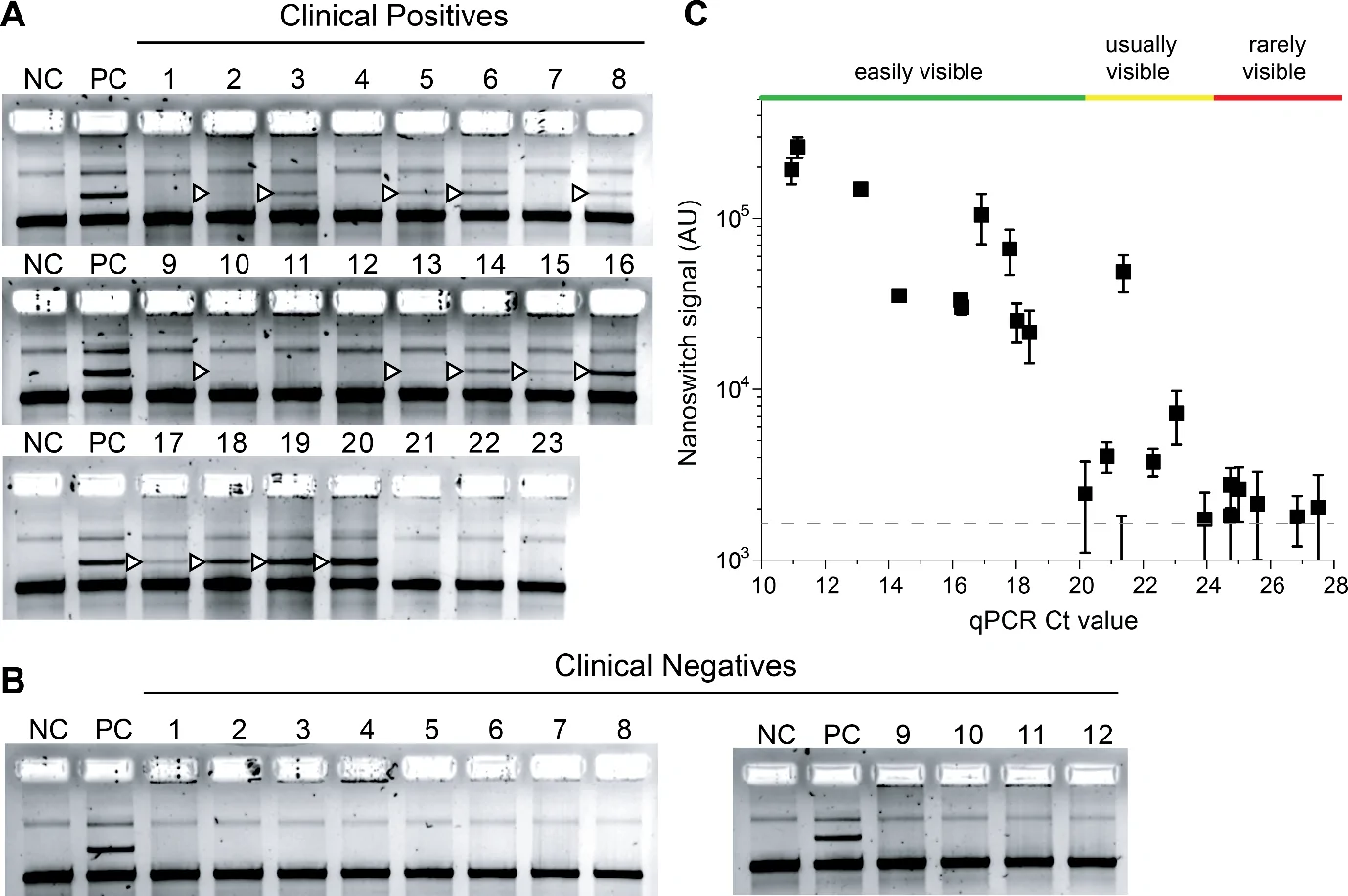
Detection of clinical SARS-CoV-2. (A) Nanoswitch based detection of RNA extracts from clinical positive samples and (B) clinical negative samples. (C) Integrated band intensity vs Ct values obtained by qPCR. Error bars represent standard deviations from three technical replicates. The dotted line represents the calculated limit of detection from the mean plus 3 standard deviations of triplicate negative controls.

## Discussion

Overall, our assay provides a unique nonenzymatic approach for direct detection of SARS-CoV-2 RNA. We have shown our method to be sensitive enough to capture a subset of positive clinical samples with moderate to high viral loads. The DNA nanoswitch approach has several advantages. The direct detection without enzymes eliminates high cost and complexity of enzymes including cold chain and provides a direct linear response that is proportional to viral load, which has been shown to be related to infectivity^72^ and severity of disease.^73^ A related advantage that became obvious during the pandemic is that our assay uses different reagents than almost all other approaches, helping to diversify against supply shortages or bottlenecks.

Our multitargeting approach provides coverage over large portions of the genome, which can enable detection even while variants continue to evolve. We have shown robust detection of the reference strain from Wuhan as well as the more recent Alpha, Delta, and Omicron variants.This is a distinct advantage over PCR-based methods, where some variants have affected the accuracy of certain diagnostic assays.^69,70,74,75^ Alternatively, the flexible design of the nanoswitch and the detectors also allows options for designing for specificity between variants, especially in those cases where there are numerous mutations. This same versatility also enables our method to be quickly adapted in response to new diseases. While it is natural to wonder about off-target effects with so many detection elements, our optimized protocol exhibits no signs of false positive detection nor of off-target detection. We suspect this derives from three main aspects: (1) low probability of exactly matched sequences, (2) enhanced stringency of binding due to gel running,^58^ and (3) off-target binding unlikely to produce signal or to substantially impact detection when 120 different elements are used.

As it stands, we believe our assay could fill a niche somewhere between diagnostic RT-qPCR and rapid antigen tests. In comparison with RT-qPCR, our test is likely to be substantially less expensive with a potentially faster turnaround, but with lower analytical sensitivity more comparable to rapid antigen tests. However, it has been argued that frequent testing is more important than very high sensitivity in limiting SARS-CoV-2 spread.^8^ There is also a clear pathway for improving our test sensitivity by processing more material with our nanoswitches. Currently, we are only testing about 1/1000th of the collected sample (based on typical dilution of a swab into 1–3 mL of media and our use of ∼2 μL of extracted RNA) so a sensitivity increase of 10–100× could be readily achievable with different sample preparation. For example, the swab could be diluted in less initial volume, the RNA extraction volumes could be modified to process more volume and elute less volume, and more volume could be used in the final nanoswitch reaction. Compared to RT-qPCR, our method has another advantage of being more tolerant of certain contaminants that are known to affect RT-qPCR (i.e., salts and detergents) and being operable without the intense cleanliness that is required of RT-qPCR. Some RT-qPCR has been affected by false positives due to sample contamination.^76^ It is also worth noting that our test performance improves with degraded RNA (to a point), a unique feature that may potentially reduce stringency of sample collection, transportation, and the testing environment. Compared to rapid antigen tests, our method has the potential to provide similar processing times and analytical sensitivity with similar or lower cost if developed into a commercial product.

This work represents the first demonstration of the DNA nanoswitches with isolates from clinical samples, and a first step toward diagnostic use for SARS-CoV-2. Further work includes making the readout more user-friendly, faster, and compatible for home or clinic use. The current total assay time as implemented here is ∼3 h (exempting RNA extraction with another ∼1 h). To illustrate the potential for improvements, we demonstrated detection of a positive sample with high viral load in less than 20 min (Figure S14). Cartridge-based bufferless gels could be a simple way to further improve or streamline the process, and we have previously demonstrated that our test can be ported to the Invitrogen E-gel system with a cell phone camera.^58^ Recent development in our lab of a 3D printed mini-gel system has shown resolution of DNA nanoswitches in as little as 2 min, in a format more compatible with point-of-care testing.^77^ Optimization of gel percentage, running voltage, and dye type could improve this method further, bringing it closer to diagnostic use. Other systems such as automated electrophoresis or capillary electrophoresis could be also adapted for this purpose. Some groups have integrated DNA nanoswitch detection into a nanopore readout,^78,79^ or a nanoswitch-like structure with a paper based lateral flow assay,^80^ illustrating that this approach need not be limited to gel electrophoresis.

Our assay is also modular, enabling it to be quickly adapted in response to new diseases. Once the sequence information on a newly emerging virus becomes available, the detectors can be designed and synthesized within a day, and the nanoswitch assembly itself takes only a few hours. This plug-and-play design will be useful not just for viral RNA detection but for a range of biomarkers and even a combined nucleic acid/antigen test for one or more viral diseases. We and others have previously demonstrated multiplexed detection of up to 6 targets using different loop sizes.^81,82^ Similar strategies could be adopted with this approach, but with the caveat that multitargeting may need to be compromised. While it would pose substantial challenges to multiplex this exact assay due to scaffold limitations, compromises such as multiplexing this assay with a single protein detection or multiplexing several RNA viruses with lower sensitivity could be readily achieved. Additionally, the nanoswitches can be stored dry or frozen without a loss of stability or functionality,^56^ making the assay useful for potential on-site testing and easy distribution. Our assay is also nonenzymatic and does not require labeling of the target or the nanoswitches, making it low-cost and useful in low-resource settings.

## Conclusion

Overall, our method provides an entirely new way to detect SARS-CoV-2 RNA that is in a class of its own for the direct detection of RNA without enzymes. The COVID-19 pandemic illustrated how over-reliance on single types of tests can cause reagent shortages and workflow bottlenecks that can negatively impact disease control.^6^ The minimalist nature of our approach requires relatively few and low-cost reagents, enabling the assay to be substantially cheaper than $1/test at scale with the nanoswitch reagent itself costing less than 1 cent per reaction. We envision that this type of test could be adapted and distributed for frequent home testing or deployed for field-surveillance testing. The programmability of the nanoswitches ensures that this method can be readily applied to other RNA viruses and makes an impact on this and future disease outbreaks.

## Materials and Methods

### DNA Nanoswitch Design

Nanoswitches were designed with “detector” oligos that hybridize 30 nt to the M13 scaffold and 15–30 nt that protrude from the nanoswitch to hybridize to a portion of the target sequence. For single target nanoswitches, a pair of detectors form a sensing element and are spaced 2580 nt away from each other on the M13. The remainder of the M13 scaffold is tiled by 118 oligos of 60 nt length, 2 oligos of 30 nt length and 1 oligo of 49 nt length.

For multitargeting nanoswitches, multiple pairs of detectors were positioned along the length such that each detector pair was separated by 2610 nt but separate pairs are offset by 30 nt. In this arrangement, the first detector pair occupies positions 1921–1950 (with an overhang at the 1950 position) and 4561–4590 (with an overhang on the 4561 position) on the M13 scaffold. The second detector pair would be shifted by 30 nt pair (occupying 1951–1980 and 4591–4620), and the 24th and final detector pair would occupy 2611–2640 and 5251–5280. Similar to the single target nanoswitch, the remainder of the M13 is tiled by hybridizing oligonucleotides. For the 24 target nanoswitches, there are the 48 detector oligos which are mixed with 96 oligos of 60 nt length and 1 oligo of 49 nt length.

### Choosing SARS-CoV-2 Targets

The first target in Figure 1 was chosen based on an original CDC primer target, which was expanded to 30 nt. For the first set of multiple targets (from the Wuhan reference genome) with 30 nt length, we used the previously reported Matlab code based on minimum distance between targets and minimal secondary structure. When we redesigned for 60 nt, we simplified the selection by searching for low predicted self-folding in a 60 nt sliding window (>−8 kcal/mol) and then selected local areas of lowest predicted self-folding while maintaining at least 100 nt between adjacent targets. Our final 120 target regions have a mean intertarget distance of 250 nt with a minimum of 132 and a maximum of 418. The predicted self-folding energies have a mean of −4.9 kcal/mol with a minimum of −8 kcal/mol and a maximum of −0.6 kcal/mol.

### DNA Nanoswitch Construction

Construction of single-target DNA nanoswitches has been extensively reported elsewhere, and we followed those methods here.^83^ For multitarget nanoswitches, the construction protocol remained the same but the oligo mixes were altered to include the multiple sets of detection oligos. All of the oligos were mixed in equimolar ratios. Unless otherwise noted, all nanoswitches were LC-purified using our previously established method.^84^

### Experimental Conditions

Reactions with DNA nanoswitches were typically carried out in 5 μL reaction volumes with 2 μL of LC purified nanoswitches (at ∼1 nM), 1 μL of 5× buffer, 1 μL of H_2_O, and 1 μL of target at 5× final concentration. The typical (1×) reaction buffer unless otherwise noted was 20 mM Tris-HCl, 30 mM MgCl_2_, 0.05% SDS, 1 μM blocking oligo. Samples were incubated for 2 h at 50 °C unless otherwise noted. For nanoswitches with 15 nt detector regions (Figures 1 and 2), samples were prestained before running the gel with gels imaged immediately after. Samples were brought to 10 μL with water and added 2 μL of 6× loading dye and 1 μL of 10× GelRed (Biotium). For nanoswitches with 30 nt detector regions (Figures 3 and 4), GelRed was omitted from the sample and gels were poststained in 1× GelRed. For IVT and clinical RNAs, fragmentation at 94 °C for 4–8 min (as noted) preceded the reaction. This was carried out in a 1× fragmentation buffer of 20 mM Tris-HCl, 3 mM MgCl_2_, 0.002 N NaOH (to adjust pH to 8.5).

### Gel Electrophoresis

Gel electrophoresis was carried out using mini gel boxes (Owl Easy Cast B2) with 0.8% agarose (Sigma A9539) in 25 mL of 0.5× TBE. 5× TBE is made in house and diluted to 0.5× and purified through a 0.2 μm filter before use. Agarose solutions were brought to a boil on a hot plate and held for at least 30 s, before being removed from heat, cooled for a few minutes, poured, and cured at room temperature for at least 45 min. Gels were run for 45 min at 75 V at room temperature unless otherwise noted. Gels with prestained samples were imaged immediately using a gel documentation system (Bio-Rad GelDoc XR+ or Azure 400). Other gels were poststained in a 1× solution of GelRed in water, with gentle shaking for at least 15 min. Gels were briefly destained and rinsed with water before being imaged.

Gel analysis was performed with ImageLab for measuring the % looped or with ImageJ for quantifying weak signals. In ImageJ, images were first filtered with a 2 pixel median filter to reduce small bright specks. A fixed-size region of interest was applied to each lane to retrieve intensity profiles, which were quantified by peak analysis in OriginLab (Figure S15). For analysis of clinical samples, one replicate of a positive sample was excluded from analysis due to likely pipetting error (replicate one of sample 3). To establish the background signal value, we used three measurements of the negative control.

### Production of SARS-CoV-2 N Gene RNA

In vitro transcribed SARS-CoV-2 nucleocapsid (N) gene RNA with a T7 promoter sequence and poly-A tail was prepared by the following method:

To provide sufficient cDNA input for in vitro transcription, N gene amplicon was prepared by PCR from a SARS-CoV-2 Positive Control (N gene) plasmid (NEB catalogue no. N2117S). N gene PCR amplicons were purified with Qiagen QIAquick PCR kit following the manufacturer’s protocol with the following modifications: PCR product was eluted with 50 μL of Qiagen Buffer EB preheated to 60 °C, allowed to soak on the column for 5 min, centrifuged at max speed (>10,000*g* for 5 min.), then the eluate was reapplied and centrifuged again as above to maximize DNA recovery.

The purified N gene PCR product was quantified by a Thermo NanoDrop 2000 UV spectrophotometer and analyzed by native 0.8% 0.5× TBE agarose gel electrophoresis poststained with 1× GelRed to evaluate sizing and integrity.

IVT RNA was prepared from 100 ng of purified N gene PCR product as input using NEB HiScribe T7 Quick Yield RNA Synthesis Kit (NEB catalogu no. E2050S) following the manufacturer’s protocol with the following modifications: 1 μL of Sigma polyvinylsulfonic acid (PVSA) (900 μg/mL) was included as an RNase inhibitor in the 20 μL IVT RNA reaction. N gene IVT RNA was DNase I treated followed by lithium chloride precipitation and resuspended in nuclease-free water following NEB HiScribe T7 Quick Yield RNA Synthesis Kit protocol. Residual LiCl and enzymes were removed from IVT RNA using Zymo RNA Clean and ConcentratorTM-5 kit (Zymo Research, Cat. No. R1015) following the manufacturer’s protocol.

### Analysis of SARS-CoV-2 RNA Controls

Eight different variants of IVT RNA controls for SARS-CoV-2 were purchased (Twist Bioscience). Controls nos. #2, 14, 23, 48, 50, 62, 65, and 70 were used, representing common variants Wuhan, Alpha, Delta, BA.1, BA.2, BA.2.1, BA.5, and BQ.1, respectively. For qPCR analysis, we used the Luna SARS-CoV-2 RT-qPCR multiplex assay kit (New England Biolabs) and followed the standard protocol. All controls were diluted from their stocks to 800,000 copies/μL and fragmented for 4 min at 94̊C in our 1× fragmentation buffer. The kit protocol was followed exactly, using 2 μL of test sample for each 20 μL reaction. The reaction was carried out on a MyGo mini qPCR machine, monitoring HEX and FAM for N1 and N2 regions, respectively. Data was analyzed using autoquantification with the included software.

### Collection and RNA Isolation from Clinical Samples

Clinical samples of nasopharyngeal swabs had been previously received at the Virology Laboratory, Wadsworth Center, from numerous clinical sources throughout New York State for clinical COVID-19 testing. Processing and analysis had been performed as previously described.^85^ Briefly, extraction on the bioMerieux EMAG was followed by RT-PCR testing on the CDC 2019 nCoV Real-Time RT-PCR Diagnostic Panel. Positive and negative samples were selected, with positive samples across a range of Ct values, and samples were deidentified prior to use in the study.

The testing of residual portions of deidentified human specimens, previously submitted to the Virology Laboratory at Wadsworth, for additional experimental assays is approved by the New York State IRB under study number 07–022. The specimens used in this study fall under this category, and patient informed consent is not required.

## Supplementary Material



## Notes

Additional experimental details and analysis, oligonucleotide sequences used (PDF)

## ○

Southern University of Science and Technology, Shenzhen 518055, China

## ◆

School of Biomedical Sciences, Li Ka Shing Faculty of Medicine, The University of Hong Kong, Hong Kong, China.

## ¶

Arbovirus Laboratory, Wadsworth Center, New York State Department of Health, Slingerlands, NY 12159, United States.

## ††

Chemistry Department, State University of New York at New Paltz, New Paltz, New York 12561, United States.
