# Supplementary material for: Nonenzymatic Detection of SARS-CoV‑2 RNA Using DNA Nanoswitches
Source: ACS Nano Med. 2026 Jul 10;1(8):1974–86. doi: 10.1021/acsnanomed.6c00053 (PMC13564866; doi:10.1021/acsnanomed.6c00053)
Supplement: Supplementary file 1 [file nm6c00053_si_001.pdf]

Supporting Information:

Non-enzymatic detection of SARS-CoV-2 RNA using DNA nanoswitches

Javier Vilcapoma[1], Asmer Aliyeva[1,^], Andrew Hayden[1,^], Arun Richard Chandrasekaran[1,2], Lifeng Zhou[1], Jibin Abraham Punnoose[1], Darren Yang[3,4,5], Clinton H. Hansen[3], Simon Chi-Chin Shiu[1], Alexis Russell[6], Kirsten St. George[6,7], Wesley P. Wong[3,4,5], Ken Halvorsen[1,*]

^equal contribution

[1]The RNA Institute, University at Albany, State University of New York, Albany, NY 12222.

[2]Department of Nanoscale Science and Engineering, University at Albany, State University of New York, Albany, NY 12222.

[3]Program in Cellular and Molecular Medicine, Boston Children's Hospital, Boston, MA 02115.

[4]Wyss Institute for Biologically Inspired Engineering, Harvard University, Boston, MA 02115.

[5]Department of Biological Chemistry and Molecular Pharmacology, Blavatnik Institute, Harvard Medical School, Boston, MA 02115.

[6]Laboratory of Viral Diseases, Wadsworth Center, New York State Department of Health, Albany, NY 12208.

[7]Department of Biomedical Science, University at Albany, State University of New York, Albany, NY 12222.

Current addresses:

Lifeng Zhou: Southern University of Science and Technology, Shenzhen 518055, China

Simon Chi-Chin Shiu: School of Biomedical Sciences, Li Ka Shing Faculty of Medicine, The University of Hong Kong, Hong Kong, China

Alexis Russell: Arbovirus Laboratory, Wadsworth Center, New York State Department of Health, Slingerlands, NY 12159

*Correspondence to khalvorsen@albany.edu

Table of Contents:

Figure S1: Schematic of the single and multi-detector DNA nanoswitch

Figure S2: Looping kinetics as a function of magnesium concentration

Figure S3: Looping kinetics as a function of temperature

Figure S4: Looping kinetics as a function of target concentration

Figure S5: Nanoswitch resolution with different buffer volumes

Figure S6: Screening loop positions for multi-detector nanoswitch

Figure S7: Testing individual targets with the five detector nanoswitch

Figure S8: Single target looping kinetics using single or multi-detector nanoswitch

Figure S9: Testing different buffers for RNA fragmentation

Figure S10: Testing IVT RNA detection with different detector lengths

Figure S11: Validating individual targets for the 30nt multi-detector nanoswitches

Figure S12: Figure S12 – qPCR analysis of SARS-CoV-2 IVT controls

Figure S13: Triplicate experiments for clinical samples

Figure S14: Rapid detection of high clinical positive

Figure S15: Imaging and analysis workflow of a gel

Table S1: Target oligo sequences

Table S2: Detector oligo sequences

Table S3: Tiling oligo sequences

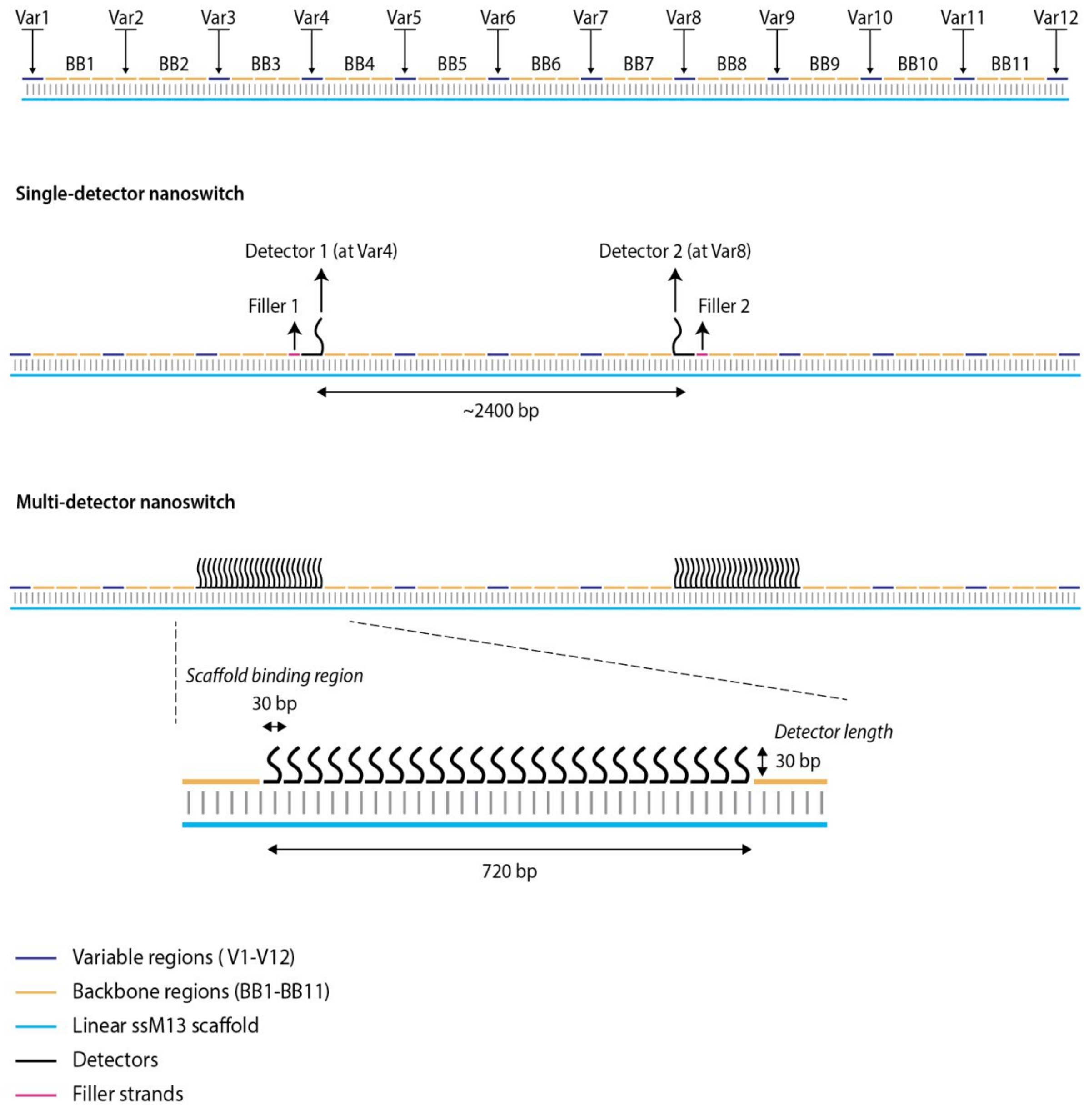

Figure S1 – Schematic of the single and multi-detector DNA nanoswitch.

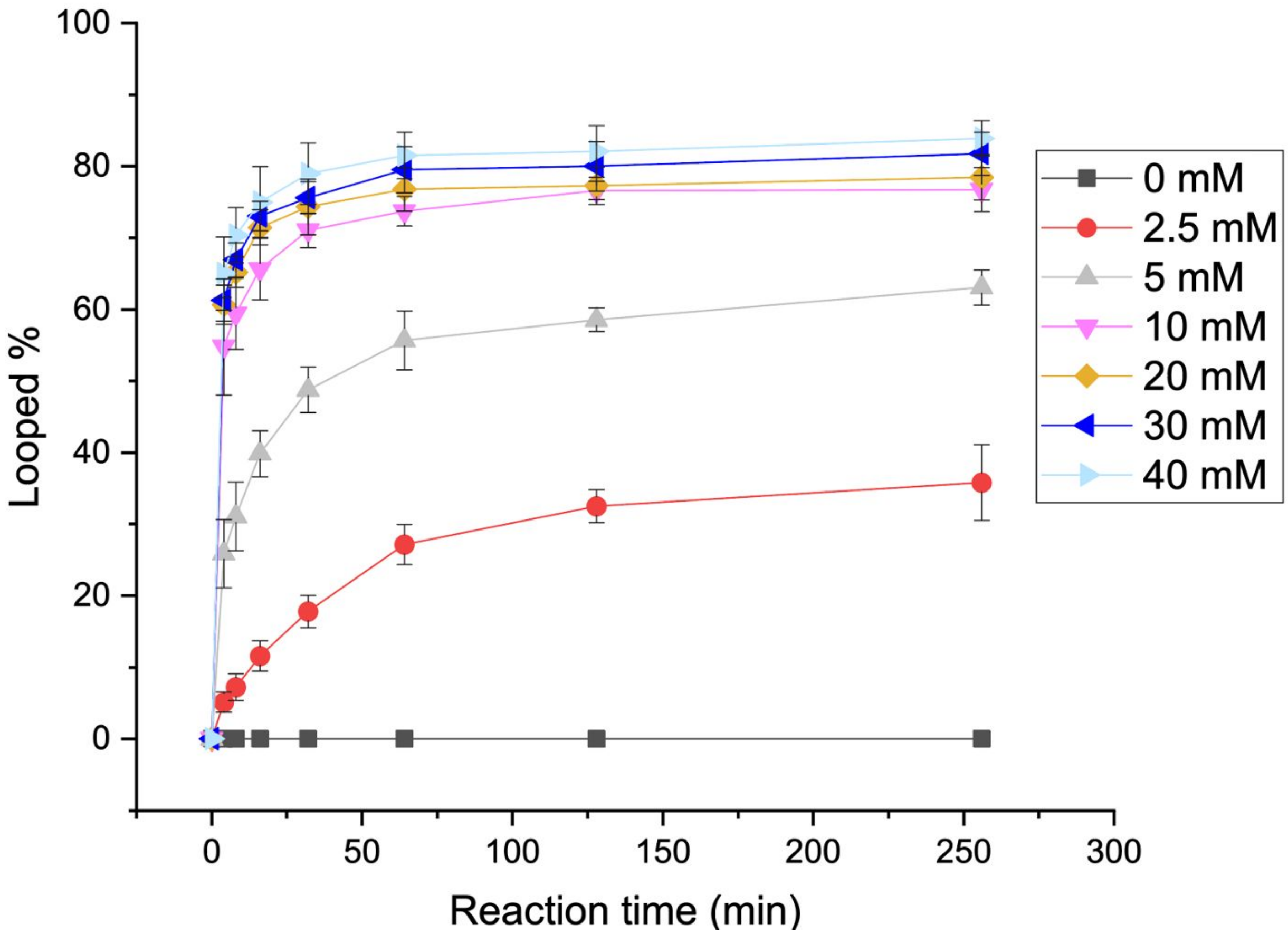

Figure S2 – Looping kinetics as a function of magnesium concentration.

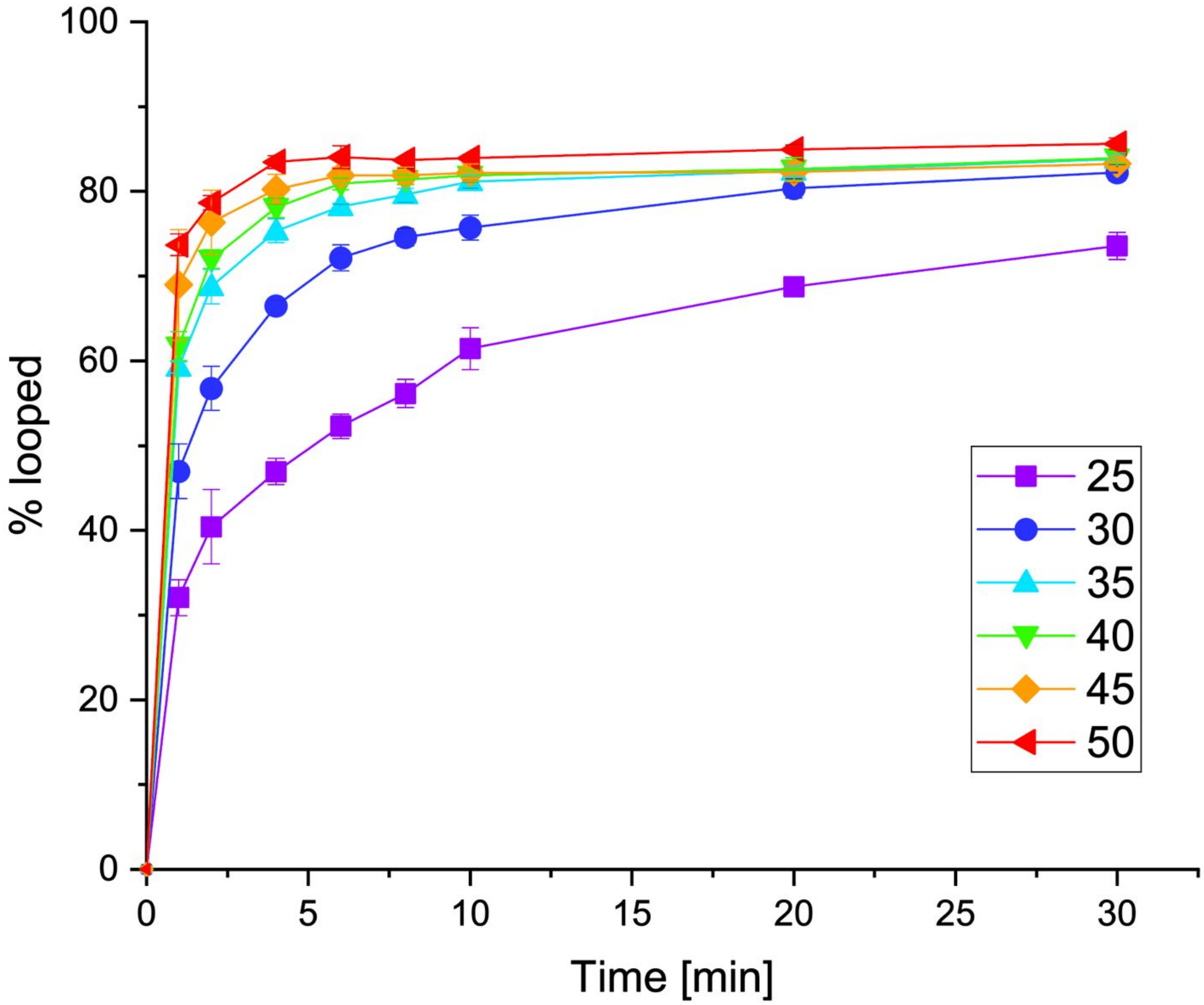

Figure S3 – Looping kinetics as a function of temperature

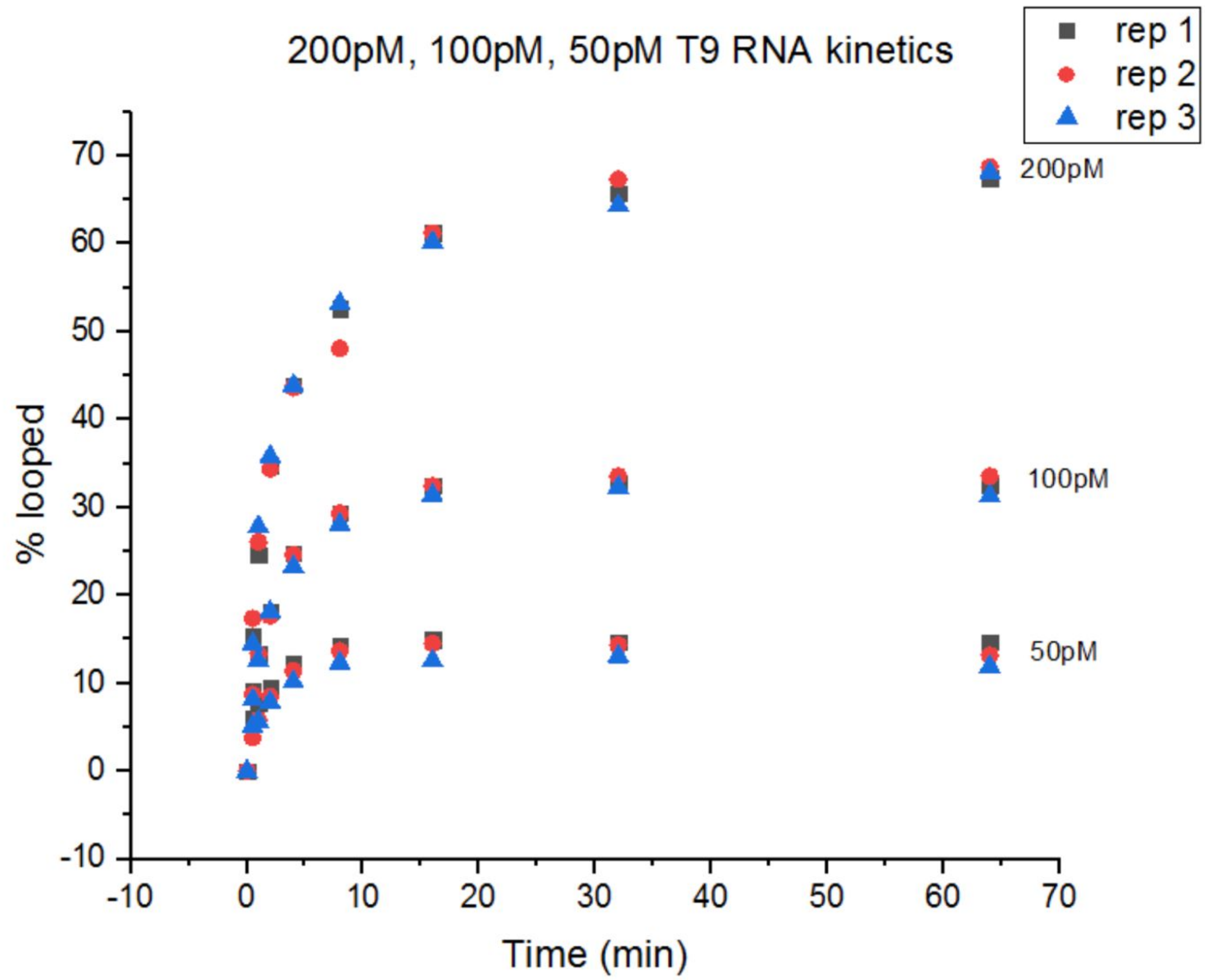

Figure S4 – Looping kinetics as a function of target concentration

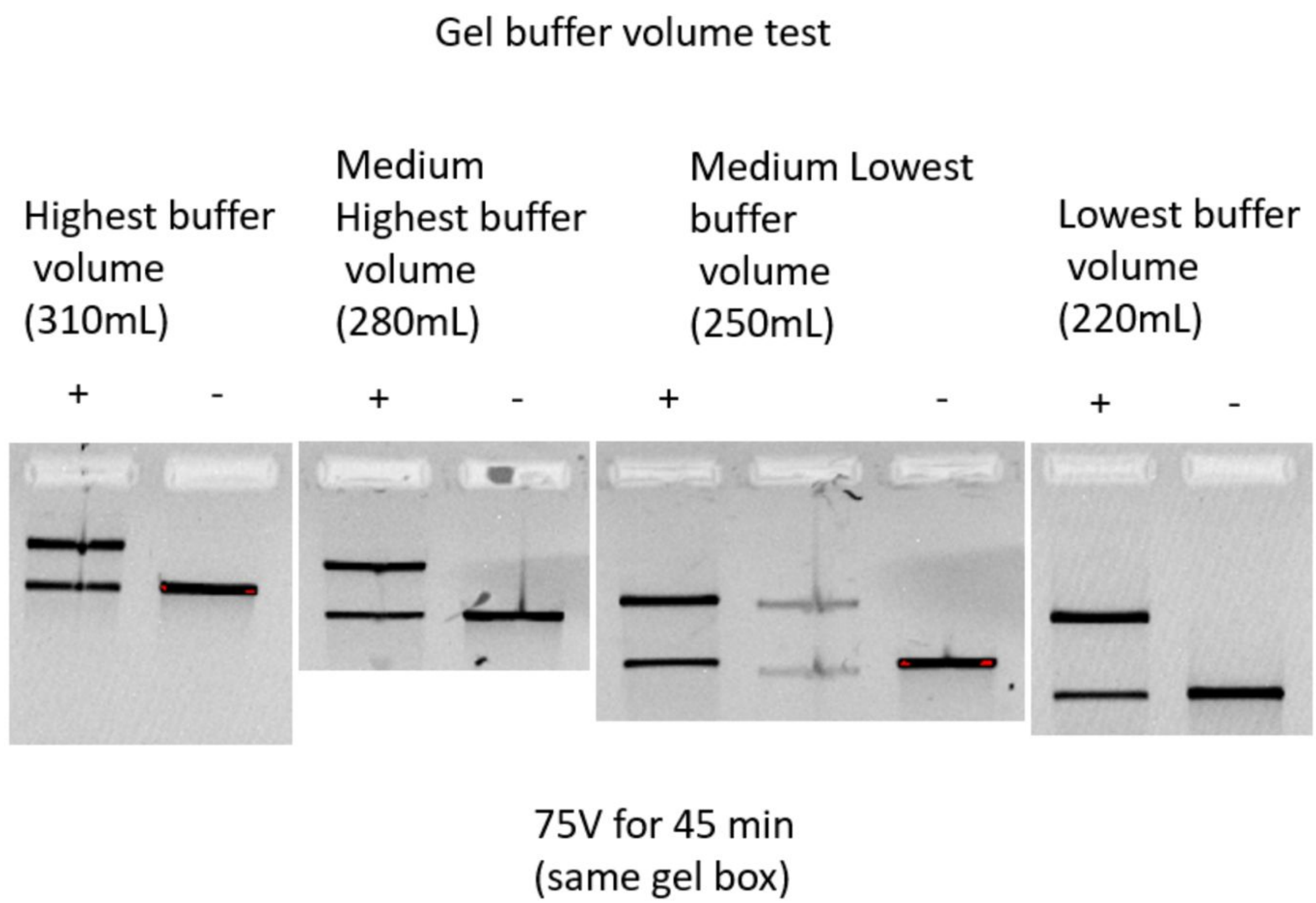

Figure S5 – Nanoswitch resolution with different buffer volumes

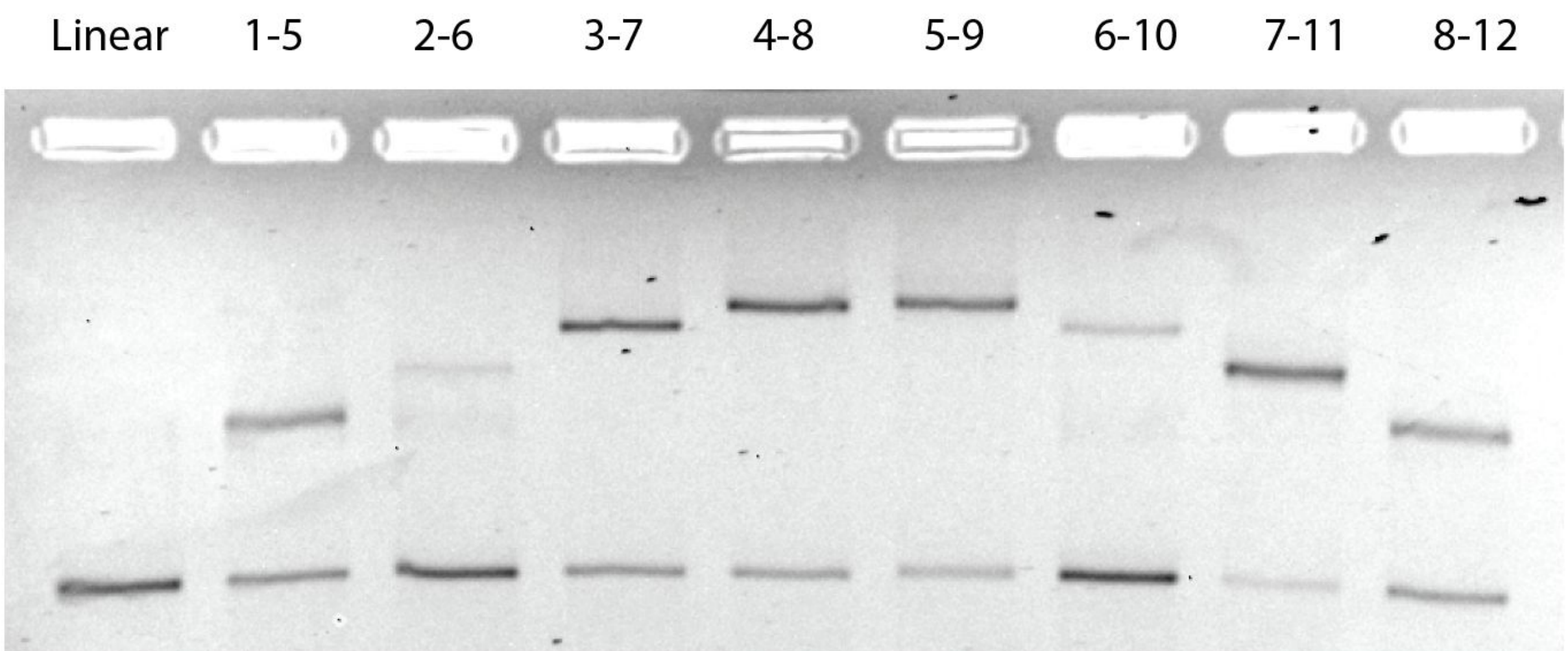

Figure S6 – Screening loop positions for multi-detector nanoswitch.

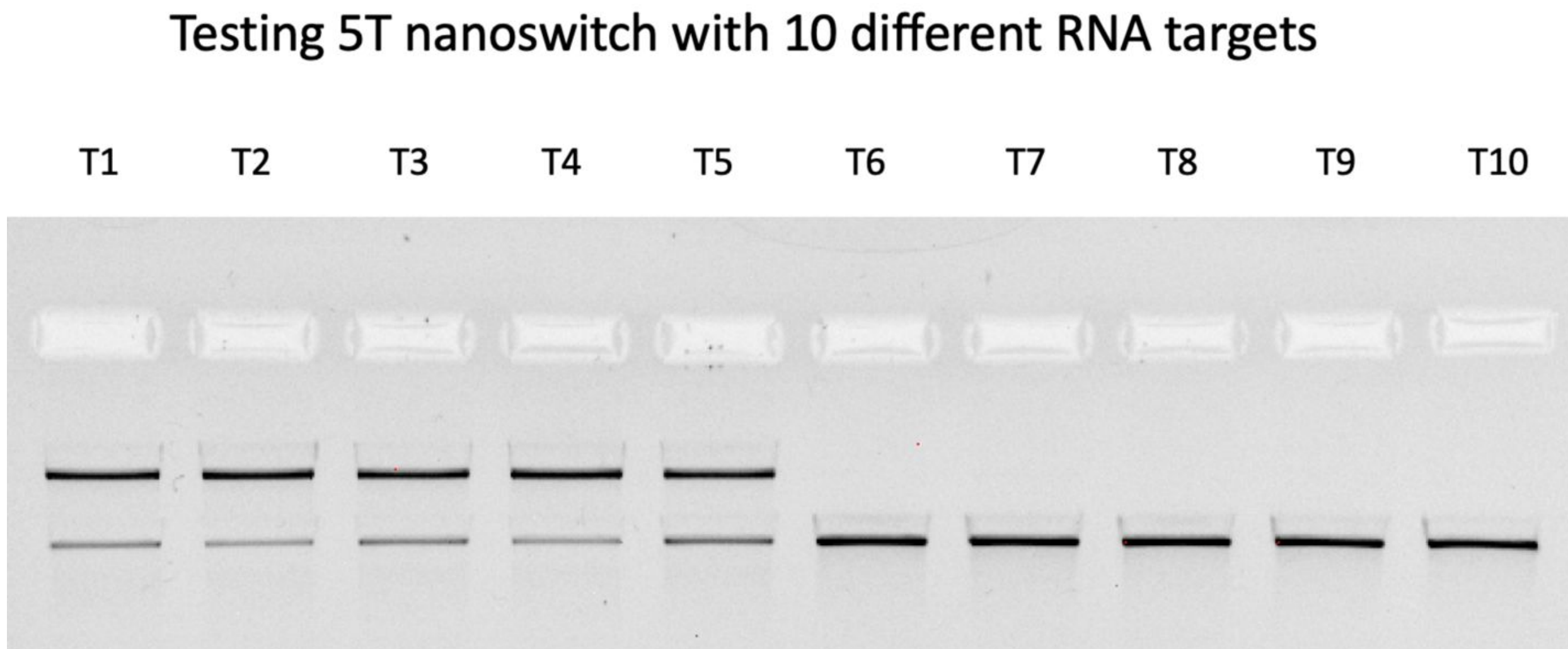

Figure S7 – Testing individual targets with the five detector nanoswitch. Targets T1 through T5 are detected, while off targets T6 through T10 are not.

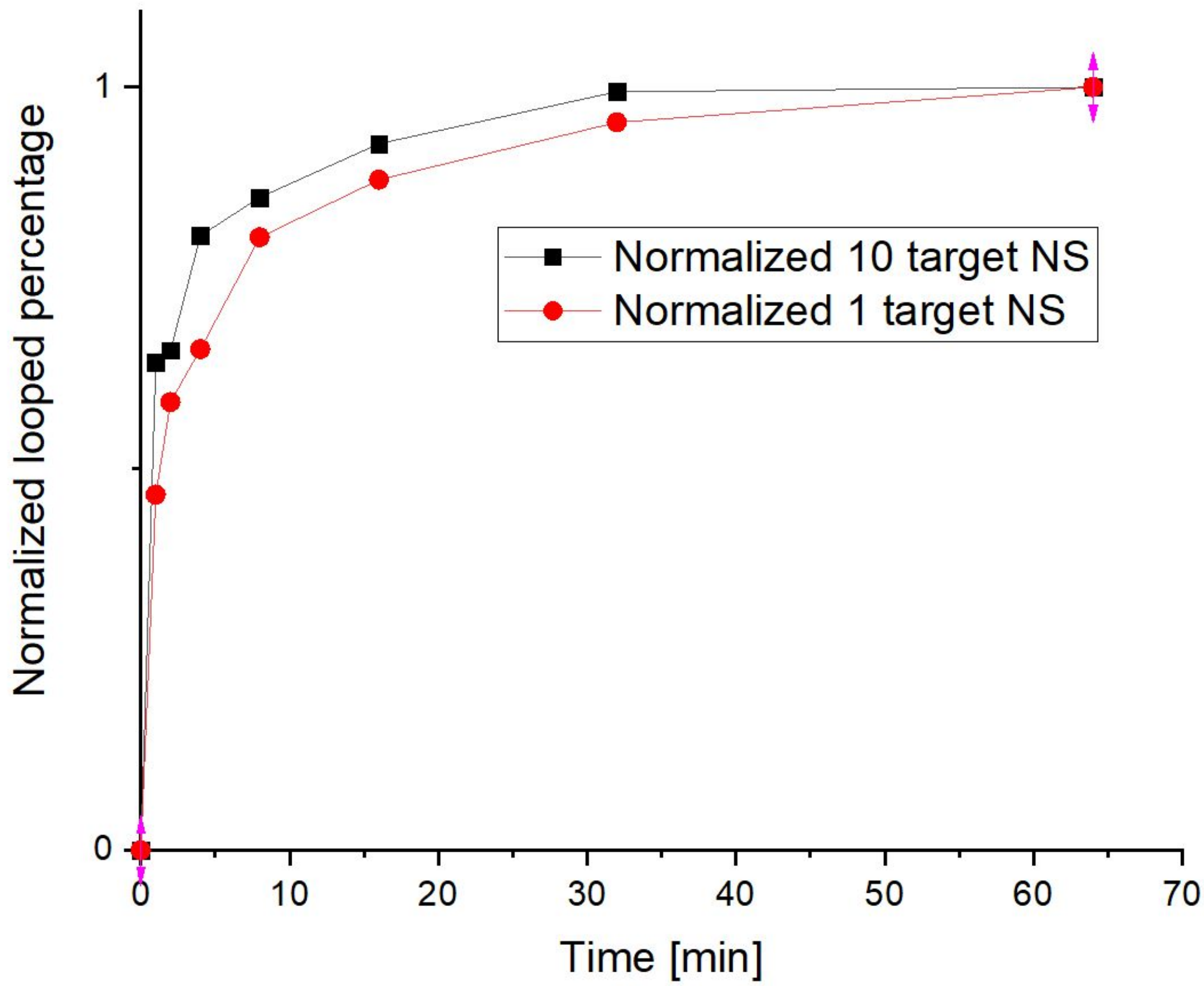

Figure S8 – Single target looping kinetics using single or multi-detector nanoswitch.

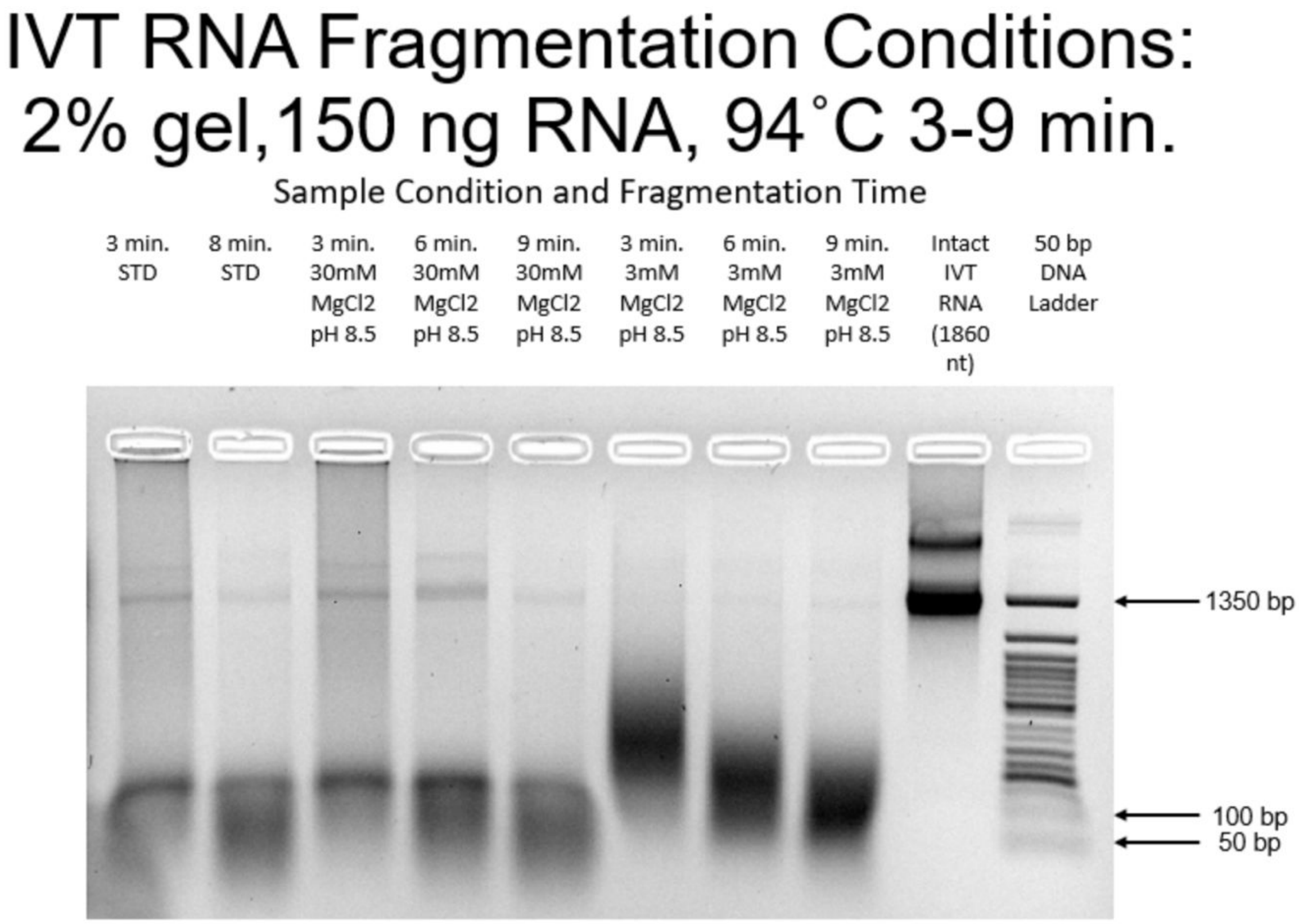

Figure S9 – Testing different buffers for RNA fragmentation

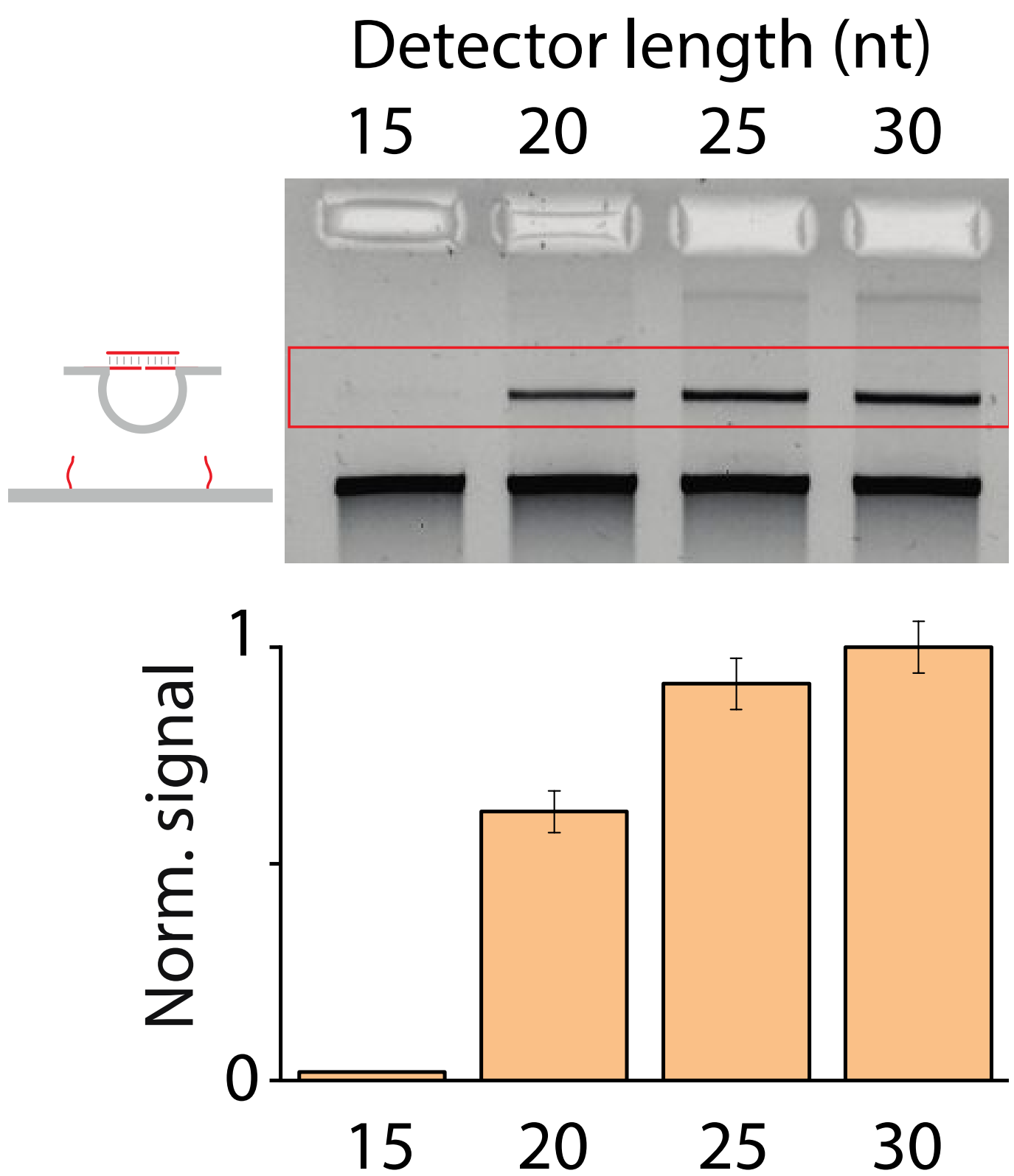

Figure S10 – Testing IVT RNA detection with different detector lengths

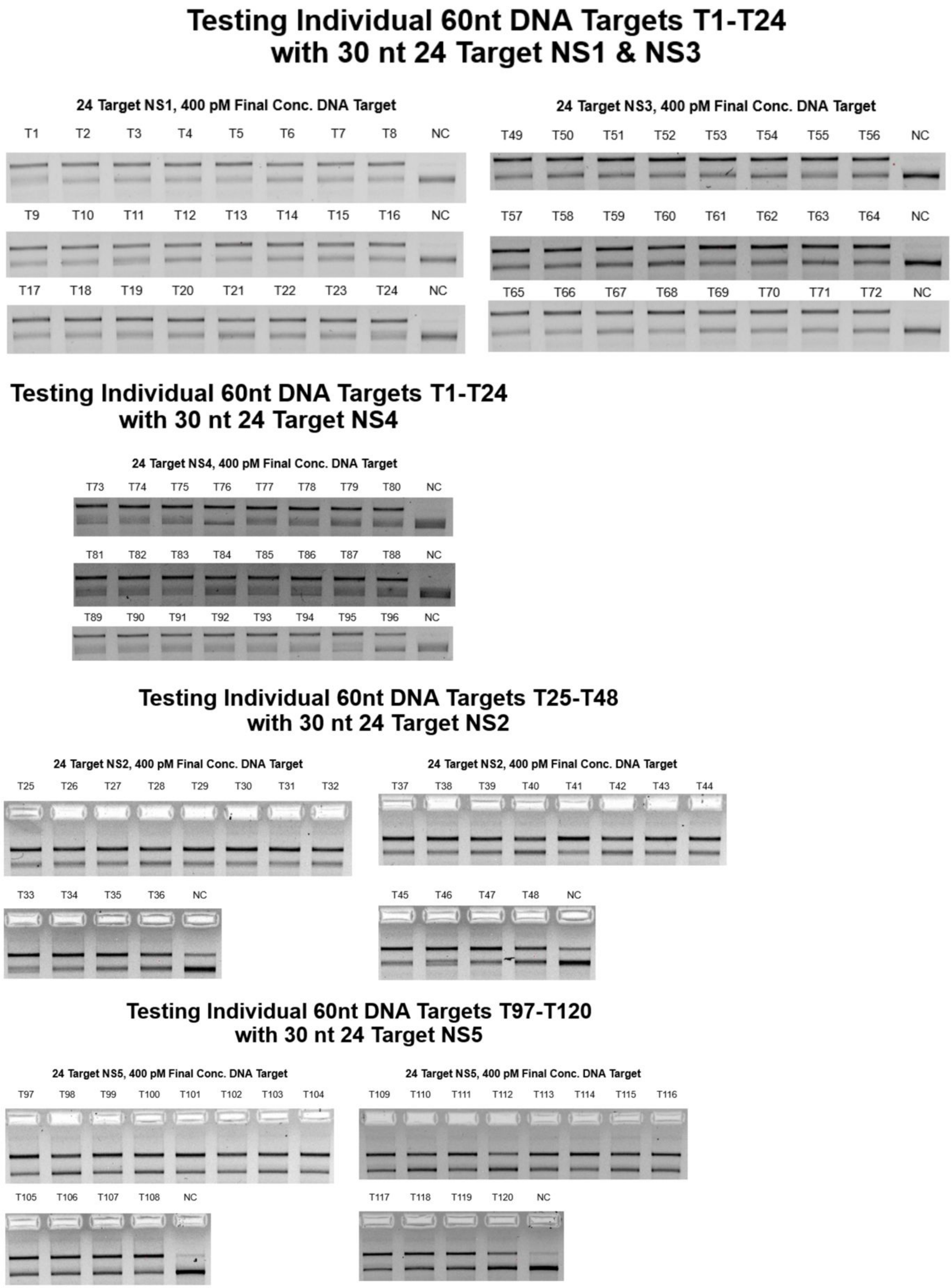

Figure S11 - Validating individual targets for the 30nt multi-detector nanoswitches

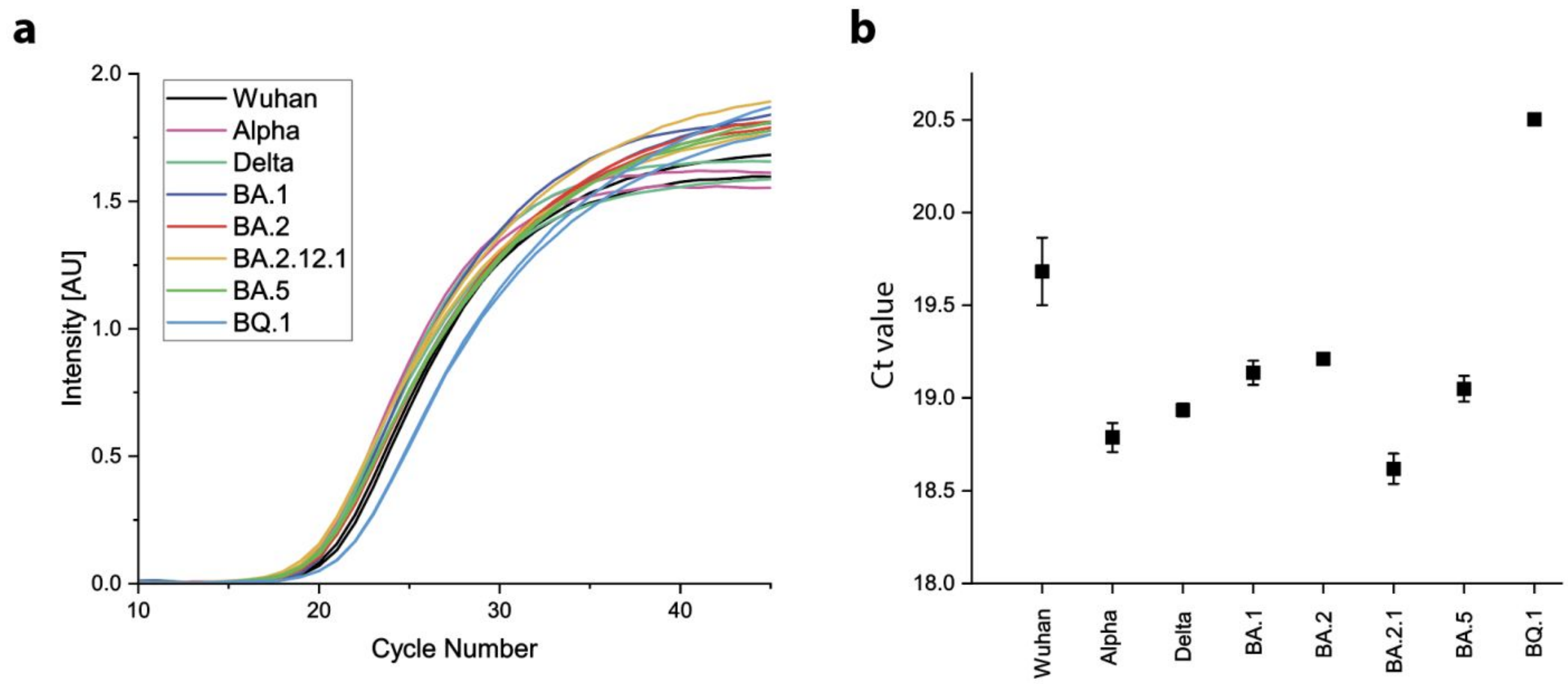

Figure S12 – qPCR analysis of SARS-CoV-2 IVT controls

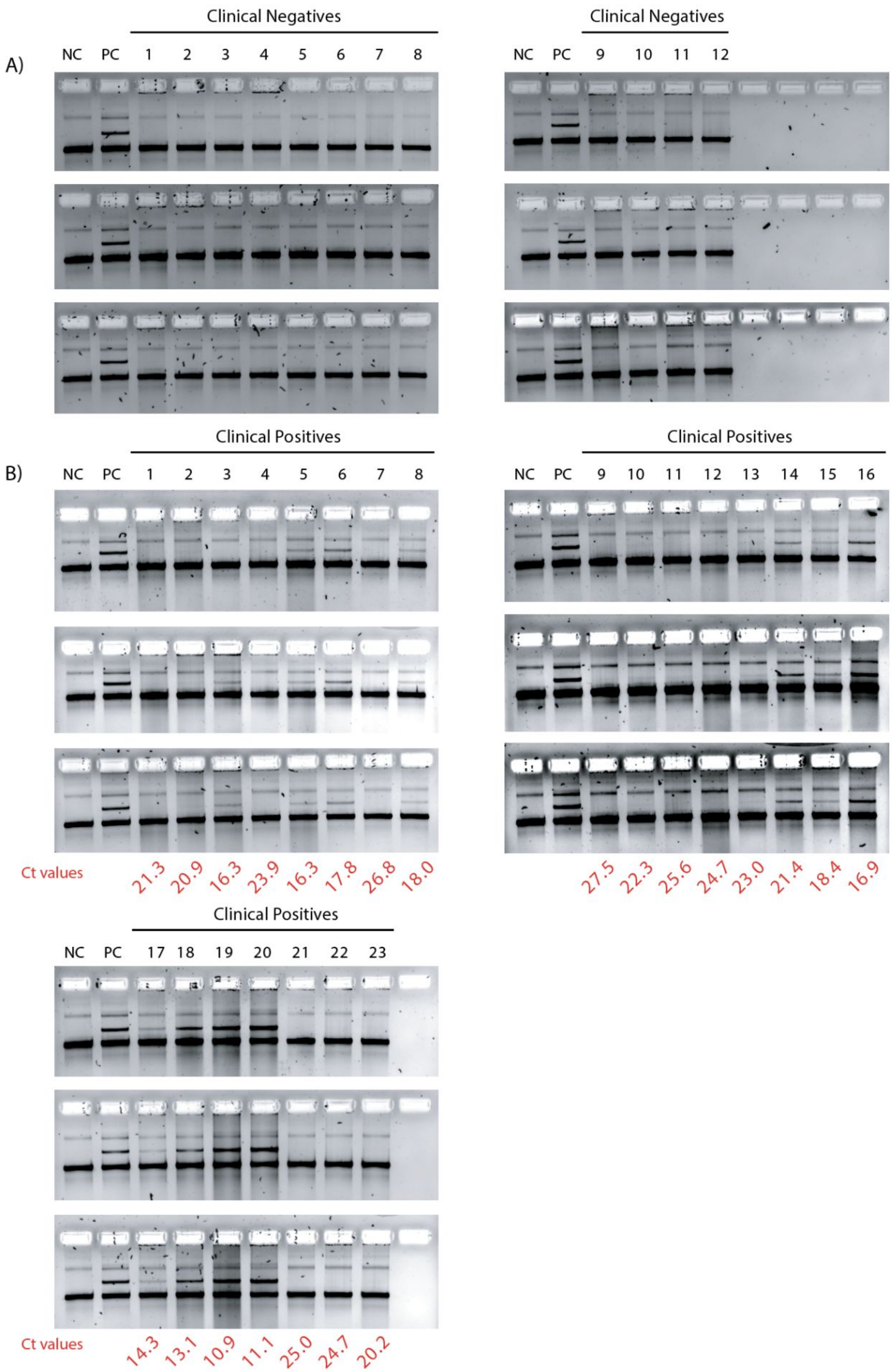

Figure S13 – Experimental triplicates of clinical samples

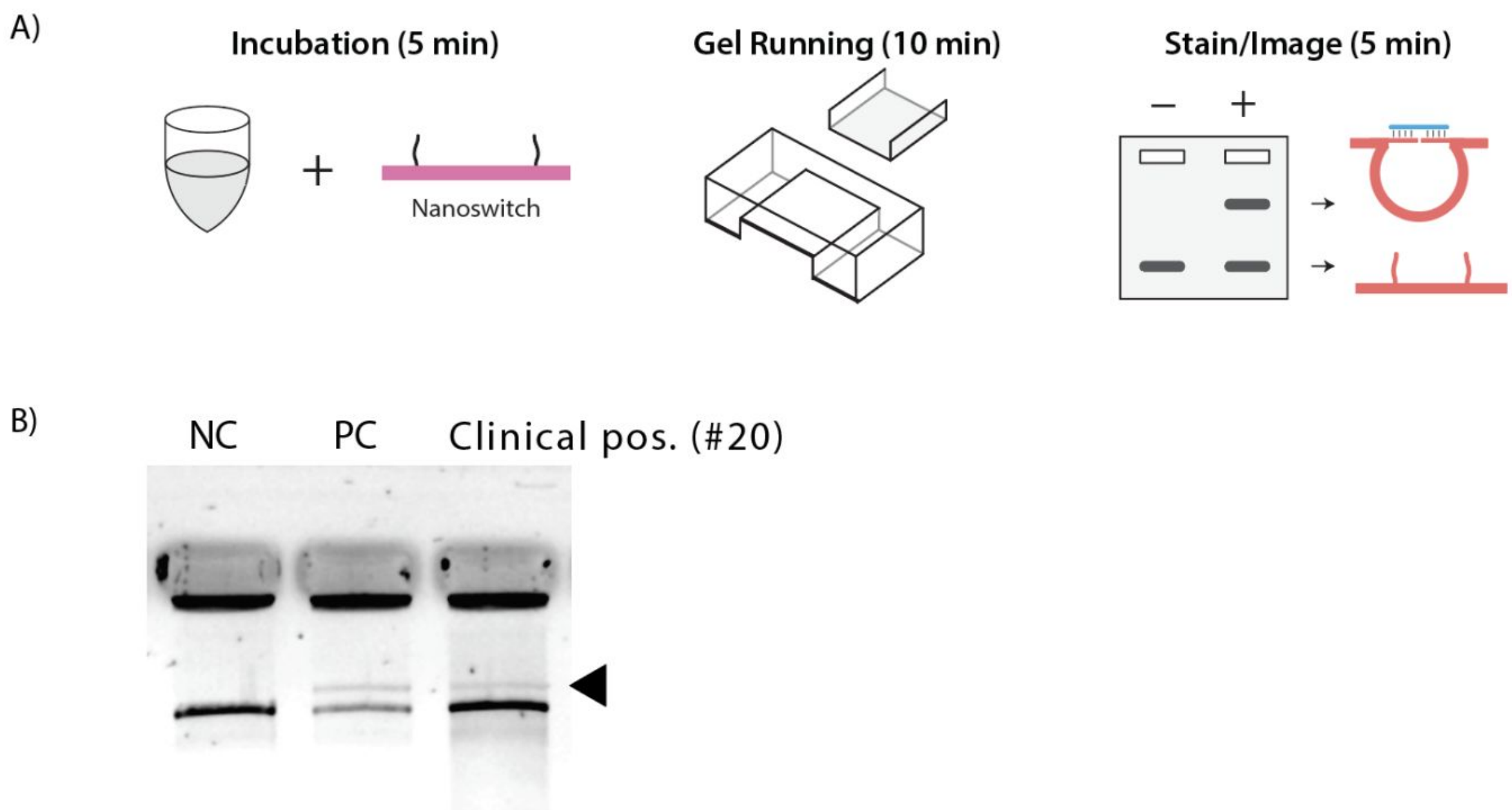

Figure S14 – Rapid detection of clinical positive

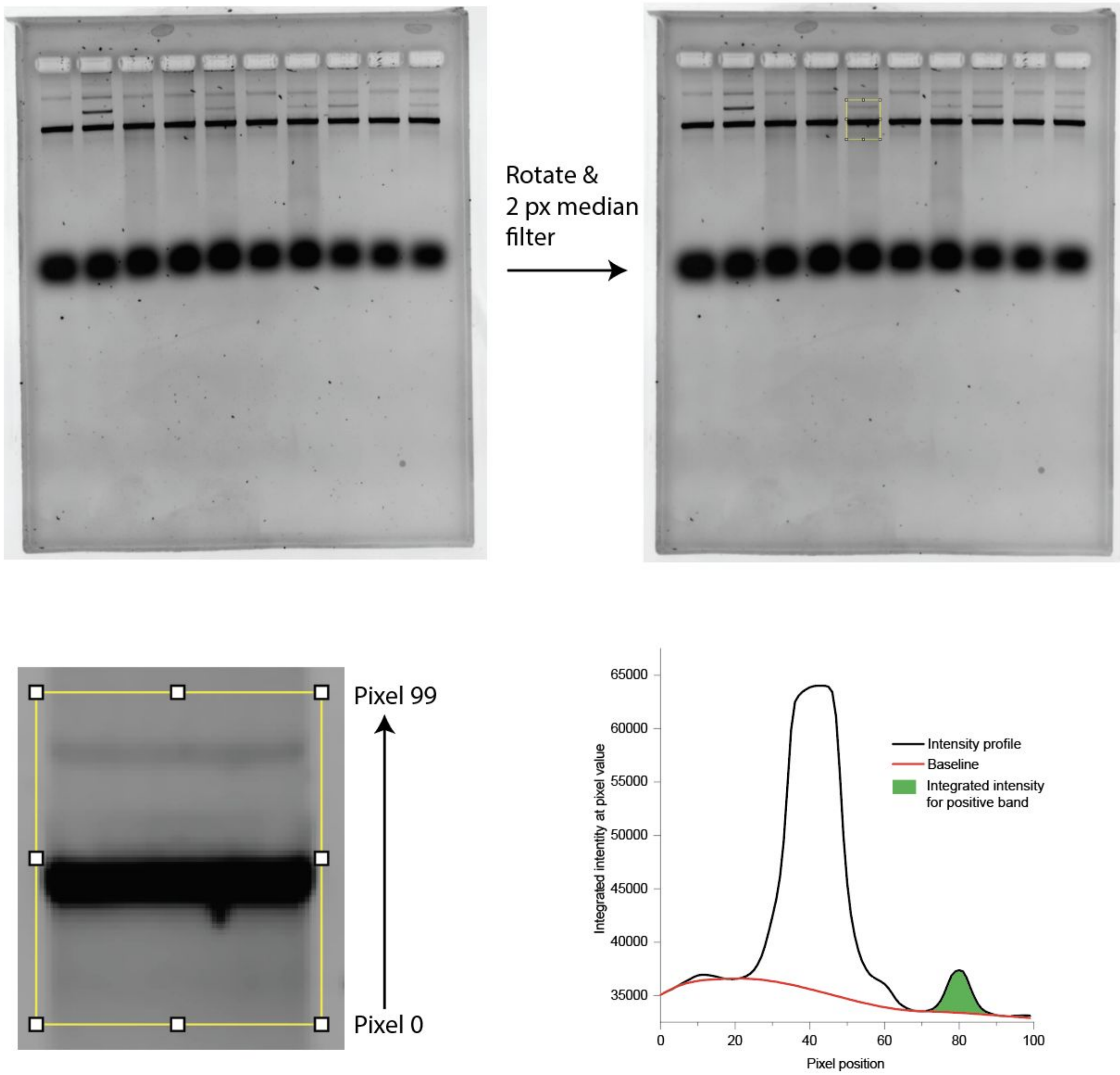

Figure S15 – Imaging and analysis workflow of a gel.

Table S1 – Target sequences

Name	Sequence	Position
Single-CDC	GACCCCAAAATCAGCGAAATGCACCCCGCA	28217
**5 Target**		
5T-1	GGATGCTCGAACTGCACCTCATGGTCATGT	487
5T-2	GCTATGTCGATAACAACTTCTGTGGCCCTG	816
5T-3	CGACAGATGTCTTGTGCTGCCGGTACTACA	12713
5T-4	ACCGTCTGCGGTATGTGGAAAGGTTATGGC	13376
5T-5	TGATTTCATACAAACCACGCCAGGTAGTGG	14100
**120 Target (30 nt)**		
**Nanoswitch 1 30 nt targets**		
NS1-30nt-T1	GGATGCTCGAACTGCACCTCATGGTCATGT	487
NS1-30nt-T2	GCTATGTCGATAACAACTTCTGTGGCCCTG	816
NS1-30nt-T3	CGACAGATGTCTTGTGCTGCCGGTACTACA	12713
NS1-30nt-T4	ACCGTCTGCGGTATGTGGAAAGGTTATGGC	13376
NS1-30nt-T5	TGATTTCATACAAACCACGCCAGGTAGTGG	14100
NS1-30nt-T6	GAGTGCTTTGTTAAGCGTGTTGACTGGACT	18889
NS1-30nt-T7	CCTCGTGAAGGTGTCTTTGTTTCAAATGGC	24830
NS1-30nt-T8	TTACACTAGCCATCCTTACTGCGCTTCGAT	26330
NS1-30nt-T9	GACCCCAAAATCAGCGAAATGCACCCCGCA	28287
NS1-30nt-T10	TCGGAATGTCGCGCATTGGCATGGAAGTCA	29217
NS1-30nt-T11	GTAACTCGTCTATCTTCTGCAGGCTGCTTA	167
NS1-30nt-T12	CCAAATGTGCCTTTCAACTCTCATGAAGTG	1204
NS1-30nt-T13	CTAGTAATCCTACCACATTCCACCTAGATG	4878
NS1-30nt-T14	TCATCTCGCAAAGGCTCTCAATGACTTCAG	9961
NS1-30nt-T15	CTGAGGACAAGAGGGCAAAAGTTACTAGTG	12318
NS1-30nt-T16	GTTCCCACCTACAAGTTTTGGACCACTAGT	14400
NS1-30nt-T17	CAGGAGATGCCACAACTGCTTATGCTAATA	15485
NS1-30nt-T18	CATCACAGGGCTCAGAATATGACTATGTCA	17840
NS1-30nt-T19	GCAACCATGATCTGTATTGTCAAGTCCATG	18803
NS1-30nt-T20	ATGTCTCTCAGCCTTTTCTTATGGACCTTG	22071
NS1-30nt-T21	TCTTATGTCCTTCCCTCAGTCAGCACCTCA	24706
NS1-30nt-T22	CGACGAGCTTGGCACTGATCCTTATGAAGA	703
NS1-30nt-T23	CTATGCACGCTGCTTCTGGTAATCTATTAC	14576
NS1-30nt-T24	TTCTTGGCACTGATAACACTCGCTACTTGT	27409
**Nanoswitch 2 30 nt targets**		
NS2-30nt-T1	CCTGGTTTCAACGAGAAAACACACGTCCAA	281
NS2-30nt-T2	CCTATGTGTTCATCAAACGTTCGGATGCTC	465
NS2-30nt-T3	ACGGTAATAAAGGAGCTGGTGGCCATAGTT	642
NS2-30nt-T4	TGGTAAAGCTTCATGCACTTTGTCCGAACA	889
NS2-30nt-T5	CTGCTGCCGTGAACATGAGCATGAAATTGC	952
NS2-30nt-T6	TGCCATAACAAGTGTGCCTATTGGGTTCCA	1511
NS2-30nt-T7	TTCGCAGTGGCTAACTAACATCTTTGGCAC	2092
NS2-30nt-T8	CCTGTGCAAAGGAAATTAAGGAGAGTGTTC	2262
NS2-30nt-T9	CACGCACTCAAAGGGATTGTACAGAAAGTG	2395
NS2-30nt-T10	GCCCTTGCACCTAATATGATGGTAACAAAC	2666
NS2-30nt-T11	TCGGTACAGAAGTAAATGAGTTCGCCTGTG	2856
NS2-30nt-T12	CAGTATCTGAATTACTTACACCACTGGGCA	2919
NS2-30nt-T13	GAATTTGGTGCCACTTCTGCTGCTCTTCAA	3146
NS2-30nt-T14	CAAGACGGCAGTGAGGACAATCAGACAACT	3236
NS2-30nt-T15	CGGACACAATCTTGCTAAACACTGTCTTCA	3583
NS2-30nt-T16	ACTGTTCGCACAAATGTCTACTTAGCTGTC	3767
NS2-30nt-T17	GCAATCTTCATCCAGATTCTGCCACTCTTG	4047
NS2-30nt-T18	TTGGAAGAAGCTGCTCGGTATATGAGATCT	4640
NS2-30nt-T19	GAAGTTTAATCCACCTGCTCTACAAGATGC	5329
NS2-30nt-T20	GTCAGAGGACGCGCAGGGAATGGATAATCT	6352
NS2-30nt-T21	CCTTGCTACTCATGGTTTAGCTGCTGTTAA	6628
NS2-30nt-T22	TCTTGTACAAATGGCCCCGATTTCAGCTAT	7375
NS2-30nt-T23	TTGCCTGGCACGATATTACGCACAACTAAT	8870
NS2-30nt-T24	TGAAAGTTTACGCCCTGACACACGTTATGT	9100
**Nanoswitch 3 30 nt targets**		
NS3-30nt-T1	TACTGTAGGCACGGCACTTGTGAAAGATCA	9212
NS3-30nt-T2	TCAACCTATTGGTGCTTTGGACATATCAGC	9370
NS3-30nt-T3	GTACTTTTGAAGAAGCTGCGCTGTGCACCT	9783

NS3-30nt-T4	CGGTCTTTGGCTTGATGACGTAGTTTACTG	10138
NS3-30nt-T5	GGTACAGGCTGGTAATGTTCAACTCAGGGT	10255
NS3-30nt-T6	TGACATACTAGGACCTCTTTCTGCTCAAAC	10795
NS3-30nt-T7	GCAAGAACTGTGTATGATGATGGTGCTAGG	11354
NS3-30nt-T8	ACTCAACCGCTACTTTAGACTGACTCTTGG	11662
NS3-30nt-T9	CAAGCTATAGCCTCAGAGTTTAGTTCCCTT	12089
NS3-30nt-T10	TGGCTAAATCTGAATTTGACCGTGATGCAG	12222
NS3-30nt-T11	GGACAATTCACCTAATTTAGCATGGCCTCT	12613
NS3-30nt-T12	AGTTTAGCTGCCACAGTACGTCTACAAGCT	12998
NS3-30nt-T13	TAACAGTTACACCGGAAGCCAATATGGATC	13188
NS3-30nt-T14	CAACTCCGCGAACCCATGCTTCAGTCAGCT	13418
NS3-30nt-T15	ATACACAATGGCAGACCTCGTCTATGCTTT	13803
NS3-30nt-T16	AATTCTGTGATGCCATGCGAAATGCTGGTA	14012
NS3-30nt-T17	CCAAGTCATCGTCAACAACCTAGACAAATC	14913
NS3-30nt-T18	ATGCCATTAGTGCAAAGAATAGAGCTCGCA	15077
NS3-30nt-T19	CCTTACCCAGATCCATCAAGAATCCTAGGG	15928
NS3-30nt-T20	CTATGTACACACCGCATACAGTCTTACAGG	16208
NS3-30nt-T21	GCAGCAGAAACGCTCAAAGCTACTGAGGAG	16636
NS3-30nt-T22	TCTTTCATGGGAAGTTGGTAAACCTAGACC	16728
NS3-30nt-T23	AGCTCTCTACTACCCTTCTGCTCGCATAGT	17121
NS3-30nt-T24	CTGTAAATGCATTGCCTGAGACGACAGCAG	17312
**Nanoswitch 4 30 nt targets**		
NS4-30nt-T1	CACTATGTGTACATTGGCGACCCTGCTCAA	17419
NS4-30nt-T2	GAAAACTATAGGTCCAGACATGTTCCTCGG	17523
NS4-30nt-T3	TCCTTACACGTAACCCTGCTTGGAGAAAAG	17732
NS4-30nt-T4	ATCCTACACAGGCACCTACACACCTCAGTG	18095
NS4-30nt-T5	GTTGACATACCTGGCATACCTAAGGACATG	18157
NS4-30nt-T6	GGTTACCCTAACATGTTTATCACCCGCGAA	18241
NS4-30nt-T7	GTGTTAACCTAGTTGCTGTACCTACAGGTT	18380
NS4-30nt-T8	GTGCTAAACCACCGCCTGGAGATCAATTTA	18449
NS4-30nt-T9	ATAGGACCTGAGCGCACCTGTTGTCTATGT	18640
NS4-30nt-T10	ATTACTCTGACAGTCCATGTGAGTCTCATG	19376
NS4-30nt-T11	GATGCTTATAACATGATGATCTCAGCTGGC	19525
NS4-30nt-T12	CTTTGATGGACAACAGGGTGAAGTACCAGT	19662
NS4-30nt-T13	ACTGTGATCTGGGACTACAAAAGAGATGCT	19867
NS4-30nt-T14	ATTACAATCTAGTCAAGCGTGGCAACCGGG	20652
NS4-30nt-T15	GTACGCTGCTTGTCGATTCAGATCTTAATG	20933
NS4-30nt-T16	CAACAAAAGCTAGCTCTTGGAGGTTCCGTG	21130
NS4-30nt-T17	CCCCCTGCATACACTAATTCTTTCACACGT	21635
NS4-30nt-T18	GGACCAATGGTACTAAGAGGTTTGATAACC	21777
NS4-30nt-T19	GATTCGAAGACCCAGTCCCTACTTATTGTT	21893
NS4-30nt-T20	CTGTAGACTGTGCACTTGACCCTCTCTCAG	22425
NS4-30nt-T21	CTATCAGGCCGGTAGCACACCTTGTAATGG	22978
NS4-30nt-T22	CTTTTGAACTTCTACATGCACCAGCAACTG	23103
NS4-30nt-T23	CCAGGAACAAATACTTCTAACCAGGTTGCT	23360
NS4-30nt-T24	CATGCAGATCAACTTACTCCTACTTGGCGT	23435
**Nanoswitch 5 30 nt targets**		
NS5-30nt-T1	GGTATATGCGCTAGTTATCAGACTCAGACT	23567
NS5-30nt-T2	GTCAATCCATCATTGCCTACACTATGTCAC	23628
NS5-30nt-T3	TTACCAGATCCATCAAAACCAAGCAAGAGG	23978
NS5-30nt-T4	TCAAGATGTGGTCAACCAAAATGCACAAGC	24406
NS5-30nt-T5	CAACCTGAATTAGACTCATTCAAGGAGGAG	24986
NS5-30nt-T6	ACTGCAACGATACCGATACAAGCCTCACTC	25486
NS5-30nt-T7	GTTTACTCACACCTTTTGCTCGTTGCTGCT	25660
NS5-30nt-T8	GATGAACCGACGACGACTACTAGCGTGCCT	26185
NS5-30nt-T9	GGCACTATTCTGACCAGACCGCTTCTAGAA	26898
NS5-30nt-T10	CCTTCGTGGACATCTTCGTATTGCTGGACA	26954
NS5-30nt-T11	AGGACCTGCCTAAAGAAATCACTGTTGCTA	27007
NS5-30nt-T12	GCACTCAATTTGCTTTTGCTTGTCCTGACG	27572
NS5-30nt-T13	ATGTAGTTGATGACCCGTGTCCTATTCACT	27985
NS5-30nt-T14	GCGCGATCAAAACAACGTCGGCCCCAAGGT	28376
NS5-30nt-T15	TCACCGCTCTCACTCAACATGGCAAGGAAG	28431
NS5-30nt-T16	GCAGTCCAGATGACCAAATTGGCTACTACC	28506
NS5-30nt-T17	CAGAAGCTGGACTTCCCTATGGTGCTAACA	28623
NS5-30nt-T18	CAAAAGATCACATTGGCACCCGCAATCCTG	28698
NS5-30nt-T19	CTCTTCTCGTTCCTCATCACGTAGTCGCAA	28819

NS5-30nt-T20	CGGCAGACGTGGTCCAGAACAAACCCAAGG	29095
NS5-30nt-T21	TGGGGACCAGGAACTAATCAGACAAGGAAC	29131
NS5-30nt-T22	CTCAAGCCTTACCGCAGAGACAGAAGAAAC	29409
NS5-30nt-T23	GACTCAACTCAGGCCTAAACTCATGCAGAC	29516
NS5-30nt-T24	TACAGTGAACAATGCTAGGGAGAGCTGCCT	29760

120 Target (60 nt)

Nanoswitch 1 60 nt targets

NS1-60nt-T1	CAAACCAACCAACTTTCGATCTCTTGTAGATCTGTTCTCTAAACGAACTTTAAAATCTGT	28
NS1-60nt-T2	GAGCCTTGTCCCTGGTTTCAACGAGAAAACACACGTCCAACTCAGTTTGCCTGTTTTACA	271
NS1-60nt-T3	TCATTTGACTTAGGCGACGAGCTTGGCACTGATCCTTATGAAGATTTTCAAGAAAACTGG	689
NS1-60nt-T4	AATGTCCAAATTTTGTATTTCCCTTAAATTCCATAATCAAGACTATTCAACCAAGGGTTG	1071
NS1-60nt-T5	TTACTTACCCCAAAATGCTGTTGTTAAAATTTATTGTCCAGCATGTCACAATTCAGAAGT	1339
NS1-60nt-T6	GGTCTTAATGACAACCTTCTTGAAATACTCCAAAAAGAGAAAGTCAACATCAATATTGTT	1598
NS1-60nt-T7	AATTTTAAAGTTACAAAAGGAAAAGCTAAAAAAGGTGCCTGGAATATTGGTGAACAGAAA	1787
NS1-60nt-T8	TAACTAACATCTTTGGCACTGTTTATGAAAAACTCAAACCCGTCCTTGATTGGCTTGAAG	2103
NS1-60nt-T9	GTTAAATCCAGAGAAGAAACTGGCCTACTCATGCCTCTAAAAGCCCCAAAAGAAATTATC	2426
NS1-60nt-T10	AGAGTGTGAATATCACTTTTGAACTTGATGAAAGGATTGATAAAGTACTTAATGAGAAGT	2775
NS1-60nt-T11	GAGGATGAAGAAGAAGGTGATTGTGAAGAAGAAGAGTTTGAGCCATCAACTCAATATGAG	3050
NS1-60nt-T12	ACAAGACGGCAGTGAGGACAATCAGACAACTACTATTCAAACAATTGTTGAGGTTCAACC	3235
NS1-60nt-T13	ACTAACAATGCCATGCAAGTTGAATCTGATGATTACATAGCTACTAATGGACCACTTAAA	3500
NS1-60nt-T14	GCTTATGAAAATTTTAATCAGCACGAAGTTCTACTTGCACCATTATTATCAGCTGGTATT	3665
NS1-60nt-T15	TTTTGGAAATGAAGAGTGAAAAGCAAGTTGAACAAAAGATCGCTGAGATTCCTAAAGAGG	3834
NS1-60nt-T16	AAGTTCCTCACAGAAAACTTGTTACTTTATATTGACATTAATGGCAATCTTCATCCAGAT	4004
NS1-60nt-T17	GAAATGCTAGCGAAAGCTTTGAGAAAAGTGCCAACAGACAATTATATAACCACTTACCCG	4196
NS1-60nt-T18	AAACTAAAGCCATAGTTTCAACTATACAGCGTAAATATAAGGGTATTAAAATACAAGAGG	4446
NS1-60nt-T19	TATCTTACTTCTTCTTCTAAAACACCTGAAGAACATTTTATTGAAACCATCTCACTTGCT	4730
NS1-60nt-T20	GATGGAGCTGATGTTACTAAAATAAAACCTCATAATTCACATGAAGGTAAAACATTTTAT	5063
NS1-60nt-T21	TAACACTCCAACAAATAGAGTTGAAGTTTAATCCACCTGCTCTACAAGATGCTTATTACA	5307
NS1-60nt-T22	TGCAAAAGAGTCTTGAACGTGGTGTGTAAAACTTGTGGACAACAGCAGACAACCCTTAAG	5495
NS1-60nt-T23	TTACCAGTGTGGTCACTATAAACATATAACTTCTAAAGAAACTTTGTATTGCATAGACGG	5755
NS1-60nt-T24	ATTCTTATTTCACAGAGCAACCAATTGATCTTGTACCAAACCAACCATATCCAAACGCAA	5976

Nanoswitch 2 60 nt targets

NS2-60nt-T1	TGATTATAAACACTACACACCCTCTTTTAAGAAAGGAGCTAAATTGTTACATAAACCTAT	6175
NS2-60nt-T2	ACTTAAACCAGCAAATAATAGTTTAAAAATTACAGAAGAGGTTGGCCACACAGATCTAAT	6493
NS2-60nt-T3	AAACCGTGTTTGTACTAATTATATGCCTTATTTCTTTACTTTATTGCTACAATTGTGTAC	6748
NS2-60nt-T4	AAACTGATAAATATTATAATTTGGTTTTTACTATTAAGTGTTTGCCTAGGTTCTTTAATC	6938
NS2-60nt-T5	TTCTTTAGACACCTATCCTTCTTTAGAAACTATACAAATTACCATTTCATCTTTTAAATG	7153
NS2-60nt-T6	GGCCCCGATTTCAGCTATGGTTAGAATGTACATCTTCTTTGCATCATTTTATTATGTATG	7387
NS2-60nt-T7	AGTTTAAAAGACCAATAAATCCTACTGACCAGTCTTCTTACATCGTTGATAGTGTTACAG	7692
NS2-60nt-T8	TATTAATGTTATAGTTTTTGATGGTAAATCAAAATGTGAAGAATCATCTGCAAAATCAGC	7876
NS2-60nt-T9	AATACGTTTTCATCAACTTTTAACGTACCAATGGAAAAACTCAAAACACTAGTTGCAACT	8051
NS2-60nt-T10	TTGTAATAACTATATGCTCACCTATAACAAAGTTGAAAACATGACACCCCGTGACCTTGG	8272
NS2-60nt-T11	GTTCCTTTTTGTTGCTGCTATTTTCTATTTAATAACACCTGTTCATGTCATGTCTAAACA	8602
NS2-60nt-T12	AACATCTGTTACACACCATCAAAACTTATAGAGTACACTGACTTTGCAACATCAGCTTGT	8945
NS2-60nt-T13	TGACACACGTTATGTGCTCATGGATGGCTCTATTATTCAATTTCCTAACACCTACCTTGA	9115
NS2-60nt-T14	TCATGTAGTTGCCTTTAATACTTTACTATTCCTTATGTCATTCACTGTACTCTGTTTAAC	9490
NS2-60nt-T15	GTTATGTTCACACCTTTAGTACCTTTCTGGATAACAATTGCTTATATCATTTGTATTTCC	9653
NS2-60nt-T16	ACTTCAGTAACTCAGGTTCTGATGTTCTTTACCAACCACCACAAACCTCTATCACCTCAG	9984
NS2-60nt-T17	TTGATACAGCCAATCCTAAGACACCTAAGTATAAGTTTGTTCGCATTCAACCAGGACAGA	10326
NS2-60nt-T18	AATGGTTCATGTGGTAGTGTTGGTTTTAACATAGATTATGACTGTGTCTCTTTTTGTTAC	10478
NS2-60nt-T19	CTATGAAGTACAATTATGAACCTCTAACACAAGACCATGTTGACATACTAGGACCTCTTT	10755
NS2-60nt-T20	GTACTCAATGGTCTTTGTTCTTTTTTTTGTATGAAAATGCCTTTTTACCTTTTGCTATGG	11055
NS2-60nt-T21	GTTTTAAGCTAAAAGACTGTGTTATGTATGCATCAGCTGTAGTGTTACTAATCCTTATGA	11292
NS2-60nt-T22	TCATGTTTTTGGCCAGAGGTATTGTTTTTATGTGTGTTGAGTATTGCCCTATTTTCTTCA	11517
NS2-60nt-T23	TACAGTCTAAAATGTCAGATGTAAAGTGCACATCAGTAGTCTTACTCTCAGTTTTGCAAC	11838
NS2-60nt-T24	GGCAACCTTACAAGCTATAGCCTCAGAGTTTAGTTCCCTTCCATCATATGCAGCTTTTGC	12079

Nanoswitch 3 60 nt targets

NS3-60nt-T1	TATGCAGACAATGCTTTTCACTATGCTTAGAAAGTTGGATAATGATGCACTCAACAACAT	12349
NS3-60nt-T2	TGAAATTAGTATGGACAATTCACCTAATTTAGCATGGCCTCTTATTGTAACAGCTTTAAG	12601
NS3-60nt-T3	TTTACAGGATTTGAAATGGGCTAGATTCCCTAAGAGTGATGGAACTGGTACTATCTATAC	12826
NS3-60nt-T4	GCCTGCCAATTCAACTGTATTATCTTTCTGTGCTTTTGCTGTAGATGCTGCTAAAGCTTA	13045
NS3-60nt-T5	ACAAATACCTACAACTTGTGCTAATGACCCTGTGGGTTTTACACTTAAAAACACAGTCTG	13315
NS3-60nt-T6	ATAAAGTAGCTGGTTTTGCTAAATTCCTAAAAACTAATTGTTGTCGCTTCCAAGAAAAGG	13559
NS3-60nt-T7	CCACATATATCACGTCAACGTCTTACTAAATACACAATGGCAGACCTCGTCTATGCTTTA	13774
NS3-60nt-T8	TATTTCAATAAAAAGGACTGGTATGATTTTGTAGAAAACCCAGATATATTACGCGTATAC	13906

NS3-60nt-T9	GGTAGTGGAGTTCCTGTTGTAGATTCTTATTATTCATTGTTAATGCCTATATTAACCTTG	14122
NS3-60nt-T10	CATTGTGCAAACTTTAATGTTTTATTCTCTACAGTGTTCCCACCTACAAGTTTTGGACCA	14365
NS3-60nt-T11	TTACTAACAATGTTGCTTTTCAAACTGTCAAACCCGGTAATTTTAACAAAGACTTCTATG	14642
NS3-60nt-T12	CTGTATTAATGCTAACCAAGTCATCGTCAACAACCTAGACAAATCAGCTGGTTTTCCATT	14898
NS3-60nt-T13	CATCCCTACTATAACTCAAATGAATCTTAAGTATGCCATTAGTGCAAAGAATAGAGCTCG	15045
NS3-60nt-T14	CATGCCTAACATGCTTAGAATTATGGCCTCACTTGTTCTTGCTCGCAAACATACAACGTG	15315
NS3-60nt-T15	ATTTGCGTAAACATTTCTCAATGATGATACTCTCTGACGATGCTGTTGTGTGTTTCAATA	15683
NS3-60nt-T16	TGATTATGTGTACCTTCCTTACCCAGATCCATCAAGAATCCTAGGGGCCGGCTGTTTTGT	15912
NS3-60nt-T17	ATTTGTACTTACAATACATAAGAAAGCTACATGATGAGTTAACAGGACACATGTTAGACA	16085
NS3-60nt-T18	AAATGCTGTTACGACCATGTCATATCAACATCACATAAATTAGTCTTGTCTGTTAATCCG	16318
NS3-60nt-T19	GTGACTGGACAAATGCTGGTGATTACATTTTAGCTAACACCTGTACTGAAAGACTCAAGC	16571
NS3-60nt-T20	AACCTAGACCACCACTTAACCGAAATTATGTCTTTACTGGTTATCGTGTAACTAAAAACA	16748
NS3-60nt-T21	TCATTTTGCTATTGGCCTAGCTCTCTACTACCCTTCTGCTCGCATAGTGTATACAGCTTG	17103
NS3-60nt-T22	CAATTACCTGCACCACGCACATTGCTAACTAAGGGCACACTAGAACCAGAATATTTCAAT	17446
NS3-60nt-T23	AATTCCTTACACGTAACCCTGCTTGGAGAAAAGCTGTCTTTATTTCACCTTATAATTCAC	17729
NS3-60nt-T24	GTAGGAATGTGGCAACTTTACAAGCTGAAAATGTAACAGGACTCTTTAAAGATTGTAGTA	18017
**Nanoswitch 4 60 nt targets**		
NS4-60nt-T1	TTTTAAAATGAATTATCAAGTTAATGGTTACCCTAACATGTTTATCACCCGCGAAGAAGC	18216
NS4-60nt-T2	GCTAAACCACCGCCTGGAGATCAATTTAAACACCTCATACCACTTATGTACAAAGGACTT	18451
NS4-60nt-T3	GTTGACATCTATGAAGTATTTTGTGAAAATAGGACCTGAGCGCACCTGTTGTCTATGTGA	18612
NS4-60nt-T4	TGTTAAGCGTGTTGACTGGACTATTGAATATCCTATAATTGGTGATGAACTGAAGATTAA	18897
NS4-60nt-T5	ACAAAGCTTATAAAATAGAAGAATTATTCTATTCTTATGCCACACATTCTGACAAATTCA	19112
NS4-60nt-T6	AAGTGCTTTTGTTAATTTAAAACAATTACCATTTTTCTATTACTCTGACAGTCCATGTGA	19338
NS4-60nt-T7	TTAGCTTGTGGGTTTACAAACAATTTGATACTTATAACCTCTGGAACACTTTTACAAGAC	19556
NS4-60nt-T8	TGACTGACATAGCCAAGAAACCAACTGAAACGATTTGTGCACCACTCACTGTCTTTTTTG	19931
NS4-60nt-T9	TTGTCCAACAATTACCTGAAACTTACTTTACTCAGAGTAGAAATTTACAAGAATTTAAAC	20174
NS4-60nt-T10	AATTAGAAGATTTTATTCCTATGGACAGTACAGTTAAAAACTATTTCATAACAGATGCGC	20411
NS4-60nt-T11	TGGTGTAAAGATGGCCATGTAGAAACATTTTACCCAAAATTACAATCTAGTCAAGCGTGG	20614
NS4-60nt-T12	TCAATATTTAAACACATTAACATTAGCTGTACCCTATAATATGAGAGTTATACATTTTGG	20811
NS4-60nt-T13	TTATTAGTGATATGTACGACCCTAAGACTAAAAATGTTACAAAAGAAAATGACTCTAAAG	21038
NS4-60nt-T14	ACGCGAACAAATAGATGGTTATGTCATGCATGCAAATTACATATTTTGGAGGAATACAAA	21303
NS4-60nt-T15	TGTTAATCTTACAACCAGAACTCAATTACCCCCTGCATACACTAATTCTTTCACACGTGG	21607
NS4-60nt-T16	GTACTACTTTAGATTCGAAGACCCAGTCCCTACTTATTGTTAATAACGCTACTAATGTTG	21882
NS4-60nt-T17	TTTAAGAATATTGATGGTTATTTTAAAATATATTCTAAGCACACGCCTATTAATTTAGTG	22142
NS4-60nt-T18	TCTTCAACCTAGGACTTTTCTATTAAAATATAATGAAAATGGAACCATTACAGATGCTGT	22369
NS4-60nt-T19	TAAGTGTTATGGAGTGTCTCCTACTAAATTAAATGATCTCTGCTTTACTAATGTCTATGC	22693
NS4-60nt-T20	AGGTTTTAATTGTTACTTTCCTTTACAATCATATGGTTTCCAACCCACTAATGGTGTTGG	23014
NS4-60nt-T21	CTTTCCAACAATTTGGCAGAGACATTGCTGACACTACTGATGCTGTCCGTGATCCACAGA	23244
NS4-60nt-T22	TGCAGATCAACTTACTCCTACTTGGCGTGTTTATTCTACAGGTTCTAATGTTTTTCAAAC	23437
NS4-60nt-T23	TAATAACTCTATTGCCATACCCACAAATTTTACTATTAGTGTTACCACAGAAATTCTACC	23686
NS4-60nt-T24	AACACCCAAGAAGTTTTTGCACAAGTCAAACAAATTTACAAAACACCACCAATTAAAGAT	23891
**Nanoswitch 5 60 nt targets**		
NS5-60nt-T1	TAACGGCCTTACTGTTTTGCCACCTTTGCTCACAGATGAAATGATTGCTCAATACACTTC	24127
NS5-60nt-T2	CTCCAATTTTGGTGCAATTTCAAGTGTTTTAAATGATATCCTTTCACGTCTTGACAAAGT	24463
NS5-60nt-T3	TATGTCCCTGCACAAGAAAAGAACTTCACAACTGCTCCTGCCATTTGTCATGATGGAAAA	24761
NS5-60nt-T4	TAATAGGAATTGTCAACAACACAGTTTATGATCCTTTGCAACCTGAATTAGACTCATTCA	24948
NS5-60nt-T5	GACCGCCTCAATGAGGTTGCCAAGAATTTAAATGAATCTCTCATCGATCTCCAAGAACTT	25112
NS5-60nt-T6	TTTGTTTATGAGAATCTTCACAATTGGAACTGTAACTTTGAAGCAAGGTGAAATCAAGGA	25398
NS5-60nt-T7	TTTACTCACACCTTTTGCTCGTTGCTGCTGGCCTTGAAGCCCCTTTTCTCTATCTTTATG	25661
NS5-60nt-T8	CCCATTACTTTATGATGCCAACTATTTTCTTTGCTGGCATACTAATTGTTACGACTATTG	25803
NS5-60nt-T9	TCAATTGAGTACAGACACTGGTGTTGAACATGTTACCTTCTTCATCTACAATAAAATTGT	26043
NS5-60nt-T10	TTAATAGTTAATAGCGTACTTCTTTTTCTTGCTTTCGTGGTATTCTTGCTAGTTACACTA	26278
NS5-60nt-T11	TAAATATTATATTAGTTTTTCTGTTTGGAACTTTAATTTTAGCCATGGCAGATTCCAACG	26479
NS5-60nt-T12	CGCGTTCCATGTGGTCATTCAATCCAGAAACTAACATTCTTCTCAACGTGCCACTCCATG	26839
NS5-60nt-T13	GGACCTGCCTAAAGAAATCACTGTTGCTACATCACGAACGCTTTCTTATTACAAATTGGG	27008
NS5-60nt-T14	TGGAATCTTGATTACATCATAAACCTCATAATTAAAAATTTATCTAAGTCACTAACTGAG	27280
NS5-60nt-T15	TTCACCATTTCATCCTCTAGCTGATAACAAATTTGCACTGACTTGCTTTAGCACTCAATT	27522
NS5-60nt-T16	CTTCTATTTGTGCTTTTTAGCCTTTCTGCTATTCCTTGTTTTAATTATGCTTATTATCTT	27779
NS5-60nt-T17	CTAAATCACCCATTCAGTACATCGATATCGGTAATTATACAGTTTCCTGTTTACCTTTTA	28093
NS5-60nt-T18	CCCCAAGGTTTACCCAATAATACTGCGTCTTGGTTCACCGCTCTCACTCAACATGGCAAG	28397
NS5-60nt-T19	CAGACGAATTCGTGGTGGTGACGGTAAAATGAAAGATCTCAGTCCAAGATGGTATTTCTA	28546
NS5-60nt-T20	TCAAGCCTCTTCTCGTTCCTCATCACGTAGTCGCAACAGTTCAAGAAATTCAACTCCAGG	28813
NS5-60nt-T21	CATACAATGTAACACAAGCTTTCGGCAGACGTGGTCCAGAACAAACCCAAGGAAATTTTG	29073
NS5-60nt-T22	CATATTGACGCATACAAAACATTCCCACCAACAGAGCCTAAAAAGGACAAAAAGAAGAAG	29339
NS5-60nt-T23	TAAACGTTTTCGCTTTTCCGTTTACGATATATAGTCTACTCTTGTGCAGAATGAATTCTC	29568
NS5-60nt-T24	GAAGAGCCCTAATGTGTAAAATTAATTTTAGTAGTGCTATCCCCATGTGATTTTAATAGC	29795

Table S2 – detector oligo sequences

Target	Detector 1 sequence (nt 1-30 hybridize M13, 31-45 with target)	Detector 2 sequence (nt 1-15 hybridize with target, 16-45 with M13)
Single-CDC	CAATACTTCTTTGATTAGTAATAACATCACACATGACCATGAGGT	GCAGTTCGAGCATCCTCAACCGATTGAGGGAGGGAAGGTAAATAT
**5 Target**		
5T-1	TTGCCTGAGTAGAAGAACTCAAACTATCGGACATGACCATGAGGT	GCAGTTCGAGCATCCTCACCGTCACCGACTTGAGCCATTTGGGAA
5T-2	CCTTGCTGGTAATATCCAGAACAATATTACCAGGGCCACAGAAGT	TGTTATCGACATAGCTTAGAGCCAGCAAAATCACCAGTAGCACCA
5T-3	CGCCAGCCATTGCAACAGGAAAAACGCTCATGTAGTACCGGCAGC	ACAAGACATCTGTCGTTACCATTAGCAAGGCCGGAAACGTCACCA
5T-4	TGGAAATACCTACATTTTGACGCTCAATCGGCCATAACCTTTCCA	CATACCGCAGACGGTATGAAACCATCGATAGCAGCACCGTAATCA
5T-5	TCTGAAATGGATTATTTACATTGGCAGATTCCACTACCTGGCGTG	GTTTGTATGAAATCAGTAGCGACAGAATCAAGTTTGCCTTTAGCG
**120 Target (30 nt)**		
**Nanoswitch 1 30 nt targets**		
NS1-30nt-T1	TTGCCTGAGTAGAAGAACTCAAACTATCGGACATGACCATGAGGT	GCAGTTCGAGCATCCTCACCGTCACCGACTTGAGCCATTTGGGAA
NS1-30nt-T2	CCTTGCTGGTAATATCCAGAACAATATTACCAGGGCCACAGAAGT	TGTTATCGACATAGCTTAGAGCCAGCAAAATCACCAGTAGCACCA
NS1-30nt-T3	CGCCAGCCATTGCAACAGGAAAAACGCTCATGTAGTACCGGCAGC	ACAAGACATCTGTCGTTACCATTAGCAAGGCCGGAAACGTCACCA
NS1-30nt-T4	TGGAAATACCTACATTTTGACGCTCAATCGGCCATAACCTTTCCA	CATACCGCAGACGGTATGAAACCATCGATAGCAGCACCGTAATCA
NS1-30nt-T5	TCTGAAATGGATTATTTACATTGGCAGATTCCACTACCTGGCGTG	GTTTGTATGAAATCAGTAGCGACAGAATCAAGTTTGCCTTTAGCG
NS1-30nt-T6	CACCAGTCACACGACCAGTAATAAAAGGGAAGTCCAGTCAACACG	CTTAACAAAGCACTCTCAGACTGTAGCGCGTTTTCATCGGCATTT
NS1-30nt-T7	CATTCTGGCCAACAGAGATAGAACCCTTCTGCCATTTGAAACAAA	GACACCTTCACGAGGTCGGTCATAGCCCCCTTATTAGCGTTTGCC
NS1-30nt-T8	GACCTGAAAGCGTAAGAATACGTGGCACAGATCGAAGCGCAGTAA	GGATGGCTAGTGTAAATCTTTTCATAATCAAAATCACCGGAACCA
NS1-30nt-T9	ACAATATTTTTGAATGGCTATTAGTCTTTATGCGGGGTGCATTTC	GCTGATTTTGGGGTCGAGCCACCACCGGAACCGCCTCCCTCAGAG
NS1-30nt-T10	ATGCGCGAACTGATAGCCCTAAAACATCGCTGACTTCCATGCCAA	TGCGCGACATTCCGACCGCCACCCTCAGAACCGCCACCCTCAGAG
NS1-30nt-T11	CATTAAAAATACCGAACGAACCACCAGCAGTAAGCAGCCTGCAGA	AGATAGACGAGTTACCCACCACCCTCAGAGCCGCCACCAGAACCA
NS1-30nt-T12	AAGATAAAACAGAGGTGAGGCGGTCAGTATCACTTCATGAGAGTT	GAAAGGCACATTTGGCCACCAGAGCCGCCGCCAGCATTGACAGGA
NS1-30nt-T13	TAACACCGCCTGCAACAGTGCCACGCTGAGCATCTAGGTGGAATG	TGGTAGGATTACTAGGGTTGAGGCAGGTCAGACGATTGGCCTTGA
NS1-30nt-T14	AGCCAGCAGCAAATGAAAAATCTAAAGCATCTGAAGTCATTGAGA	GCCTTTGCGAGATGATATTCACAAACAAATAAATCCTCATTAAAG
NS1-30nt-T15	CACCTTGCTGAACCTCAAATATCAAACCCTCACTAGTAACTTTTG	CCCTCTTGTCCTCAGCCAGAATGGAAAGCGCAGTCTCTGAATTTA
NS1-30nt-T16	CAATCAATATCTGGTCAGTTGGCAAATCAAACTAGTGGTCCAAAA	CTTGTAGGTGGGAACCCGTTCCAGTAAGCGTCATACATGGCTTTT
NS1-30nt-T17	CAGTTGAAAGGAATTGAGGAAGGTTATCTATATTAGCATAAGCAG	TTGTGGCATCTCCTGGATGATACAGGAGTGTACTGGTAATAAGTT
NS1-30nt-T18	AAATATCTTTAGGAGCACTAACAACTAATATGACATAGTCATATT	CTGAGCCCTGTGATGTTAACGGGGTCAGTGCCTTGAGTAACAGTG
NS1-30nt-T19	GATTAGAGCCGTCAATAGATAATACATTTGCATGGACTTGACAAT	ACAGATCATGGTTGCCCCGTATAAACAGTTAATGCCCCCTGCCTA
NS1-30nt-T20	AGGATTTAGAAGTATTAGACTTTACAAACACAAGGTCCATAAGAA	AAGGCTGAGAGACATTTTCGGAACCTATTATTCTGAAACATGAAA
NS1-30nt-T21	TCTGTCCATCACGCAAATTAACCGTTGTAGTGAGGTGCTGACTGA	GGGAAGGACATAAGATCAACCGATTGAGGGAGGGAAGGTAAATAT
NS1-30nt-T22	CAATACTTCTTTGATTAGTAATAACATCACTCTTCATAAGGATCA	GTGCCAAGCTCGTCGTGACGGAAATTATTCATTAAAGGTGAATTA
NS1-30nt-T23	ATTCGACAACTCGTATTAAATCCTTTGCCCGTAATAGATTACCAG	AAGCAGCGTGCATAGGTATTAAGAGGCTGAGACTCCTCAAGAGAA
NS1-30nt-T24	GAACGTTATTAATTTTAAAAGTTTGAGTAAACAAGTAGCGAGTGT	TATCAGTGCCAAGAAGGATTAGGATTAGCGGGGTTTTGCTCAGTA
**Nanoswitch 2 30 nt targets**		
NS2-30nt-T1	TCTGTCCATCACGCAAATTAACCGTTGTAGTTGGACGTGTGTTTT	CTCGTTGAAACCAGGTCAACCGATTGAGGGAGGGAAGGTAAATAT
NS2-30nt-T2	CAATACTTCTTTGATTAGTAATAACATCACGAGCATCCGAACGTT	TGATGAACACATAGGTGACGGAAATTATTCATTAAAGGTGAATTA
NS2-30nt-T3	TTGCCTGAGTAGAAGAACTCAAACTATCGGAACTATGGCCACCAG	CTCCTTTATTACCGTTCACCGTCACCGACTTGAGCCATTTGGGAA
NS2-30nt-T4	CCTTGCTGGTAATATCCAGAACAATATTACTGTTCGGACAAAGTG	CATGAAGCTTTACCATTAGAGCCAGCAAAATCACCAGTAGCACCA
NS2-30nt-T5	CGCCAGCCATTGCAACAGGAAAAACGCTCAGCAATTTCATGCTCA	TGTTCACGGCAGCAGTTACCATTAGCAAGGCCGGAAACGTCACCA
NS2-30nt-T6	TGGAAATACCTACATTTTGACGCTCAATCGTGGAACCCAATAGGC	ACACTTGTTATGGCAATGAAACCATCGATAGCAGCACCGTAATCA
NS2-30nt-T7	TCTGAAATGGATTATTTACATTGGCAGATTGTGCCAAAGATGTTA	GTTAGCCACTGCGAAGTAGCGACAGAATCAAGTTTGCCTTTAGCG
NS2-30nt-T8	CACCAGTCACACGACCAGTAATAAAAGGGAGAACACTCTCCTTAA	TTTCCTTTGCACAGGTCAGACTGTAGCGCGTTTTCATCGGCATTT
NS2-30nt-T9	CATTCTGGCCAACAGAGATAGAACCCTTCTCACTTTCTGTACAAT	CCCTTTGAGTGCGTGTCGGTCATAGCCCCCTTATTAGCGTTTGCC
NS2-30nt-T10	GACCTGAAAGCGTAAGAATACGTGGCACAGGTTTGTTACCATCAT	ATTAGGTGCAAGGGCATCTTTTCATAATCAAAATCACCGGAACCA
NS2-30nt-T11	ACAATATTTTTGAATGGCTATTAGTCTTTACACAGGCGAACTCAT	TTACTTCTGTACCGAGAGCCACCACCGGAACCGCCTCCCTCAGAG
NS2-30nt-T12	ATGCGCGAACTGATAGCCCTAAAACATCGCTGCCCAGTGGTGTAA	GTAATTCAGATACTGCCGCCACCCTCAGAACCGCCACCCTCAGAG
NS2-30nt-T13	CATTAAAAATACCGAACGAACCACCAGCAGTTGAAGAGCAGCAGA	AGTGGCACCAAATTCCCACCACCCTCAGAGCCGCCACCAGAACCA
NS2-30nt-T14	AAGATAAAACAGAGGTGAGGCGGTCAGTATAGTTGTCTGATTGTC	CTCACTGCCGTCTTGCCACCAGAGCCGCCGCCAGCATTGACAGGA
NS2-30nt-T15	TAACACCGCCTGCAACAGTGCCACGCTGAGTGAAGACAGTGTTTA	GCAAGATTGTGTCCGGGTTGAGGCAGGTCAGACGATTGGCCTTGA
NS2-30nt-T16	AGCCAGCAGCAAATGAAAAATCTAAAGCATGACAGCTAAGTAGAC	ATTTGTGCGAACAGTTATTCACAAACAAATAAATCCTCATTAAAG
NS2-30nt-T17	CACCTTGCTGAACCTCAAATATCAAACCCTCAAGAGTGGCAGAAT	CTGGATGAAGATTGCCCAGAATGGAAAGCGCAGTCTCTGAATTTA
NS2-30nt-T18	CAATCAATATCTGGTCAGTTGGCAAATCAAAGATCTCATATACCG	AGCAGCTTCTTCCAACCGTTCCAGTAAGCGTCATACATGGCTTTT
NS2-30nt-T19	CAGTTGAAAGGAATTGAGGAAGGTTATCTAGCATCTTGTAGAGCA	GGTGGATTAAACTTCGATGATACAGGAGTGTACTGGTAATAAGTT
NS2-30nt-T20	AAATATCTTTAGGAGCACTAACAACTAATAAGATTATCCATTCCC	TGCGCGTCCTCTGACTTAACGGGGTCAGTGCCTTGAGTAACAGTG
NS2-30nt-T21	GATTAGAGCCGTCAATAGATAATACATTTGTTAACAGCAGCTAAA	CCATGAGTAGCAAGGCCCGTATAAACAGTTAATGCCCCCTGCCTA
NS2-30nt-T22	AGGATTTAGAAGTATTAGACTTTACAAACAATAGCTGAAATCGGG	GCCATTTGTACAAGATTTCGGAACCTATTATTCTGAAACATGAAA

NS2-30nt-T23	ATTCGACAACTCGTATTAAATCCTTTGCCCATTAGTTGTGCGTAA	TATCGTGCCAGGCAAGTATTAAGAGGCTGAGACTCCTCAAGAGAA
NS2-30nt-T24	GAACGTTATTAATTTTAAAAGTTTGAGTAAACATAACGTGTGTCA	GGGCGTAAACTTTCAGGATTAGGATTAGCGGGGTTTTGCTCAGTA
**Nanoswitch 3 30 nt targets**		
NS3-30nt-T1	TCTGTCCATCACGCAAATTAACCGTTGTAGTGATCTTTCACAAGT	GCCGTGCCTACAGTATCAACCGATTGAGGGAGGGAAGGTAAATAT
NS3-30nt-T2	CAATACTTCTTTGATTAGTAATAACATCACGCTGATATGTCCAAA	GCACCAATAGGTTGATGACGGAAATTATTCATTAAAGGTGAATTA
NS3-30nt-T3	TTGCCTGAGTAGAAGAACTCAAACTATCGGAGGTGCACAGCGCAG	CTTCTTCAAAAGTACTCACCGTCACCGACTTGAGCCATTTGGGAA
NS3-30nt-T4	CCTTGCTGGTAATATCCAGAACAATATTACCAGTAAACTACGTCA	TCAAGCCAAAGACCGTTAGAGCCAGCAAAATCACCAGTAGCACCA
NS3-30nt-T5	CGCCAGCCATTGCAACAGGAAAAACGCTCAACCCTGAGTTGAACA	TTACCAGCCTGTACCTTACCATTAGCAAGGCCGGAAACGTCACCA
NS3-30nt-T6	TGGAAATACCTACATTTTGACGCTCAATCGGTTTGAGCAGAAAGA	GGTCCTAGTATGTCAATGAAACCATCGATAGCAGCACCGTAATCA
NS3-30nt-T7	TCTGAAATGGATTATTTACATTGGCAGATTCCTAGCACCATCATC	ATACACAGTTCTTGCGTAGCGACAGAATCAAGTTTGCCTTTAGCG
NS3-30nt-T8	CACCAGTCACACGACCAGTAATAAAAGGGACCAAGAGTCAGTCTA	AAGTAGCGGTTGAGTTCAGACTGTAGCGCGTTTTCATCGGCATTT
NS3-30nt-T9	CATTCTGGCCAACAGAGATAGAACCCTTCTAAGGGAACTAAACTC	TGAGGCTATAGCTTGTCGGTCATAGCCCCCTTATTAGCGTTTGCC
NS3-30nt-T10	GACCTGAAAGCGTAAGAATACGTGGCACAGCTGCATCACGGTCAA	ATTCAGATTTAGCCAATCTTTTCATAATCAAAATCACCGGAACCA
NS3-30nt-T11	ACAATATTTTTGAATGGCTATTAGTCTTTAAGAGGCCATGCTAAA	TTAGGTGAATTGTCCGAGCCACCACCGGAACCGCCTCCCTCAGAG
NS3-30nt-T12	ATGCGCGAACTGATAGCCCTAAAACATCGCAGCTTGTAGACGTAC	TGTGGCAGCTAAACTCCGCCACCCTCAGAACCGCCACCCTCAGAG
NS3-30nt-T13	CATTAAAAATACCGAACGAACCACCAGCAGGATCCATATTGGCTT	CCGGTGTAACTGTTACCACCACCCTCAGAGCCGCCACCAGAACCA
NS3-30nt-T14	AAGATAAAACAGAGGTGAGGCGGTCAGTATAGCTGACTGAAGCAT	GGGTTCGCGGAGTTGCCACCAGAGCCGCCGCCAGCATTGACAGGA
NS3-30nt-T15	TAACACCGCCTGCAACAGTGCCACGCTGAGAAAGCATAGACGAGG	TCTGCCATTGTGTATGGTTGAGGCAGGTCAGACGATTGGCCTTGA
NS3-30nt-T16	AGCCAGCAGCAAATGAAAAATCTAAAGCATTACCAGCATTTCGCA	TGGCATCACAGAATTTATTCACAAACAAATAAATCCTCATTAAAG
NS3-30nt-T17	CACCTTGCTGAACCTCAAATATCAAACCCTGATTTGTCTAGGTTG	TTGACGATGACTTGGCCAGAATGGAAAGCGCAGTCTCTGAATTTA
NS3-30nt-T18	CAATCAATATCTGGTCAGTTGGCAAATCAATGCGAGCTCTATTCT	TTGCACTAATGGCATCCGTTCCAGTAAGCGTCATACATGGCTTTT
NS3-30nt-T19	CAGTTGAAAGGAATTGAGGAAGGTTATCTACCCTAGGATTCTTGA	TGGATCTGGGTAAGGGATGATACAGGAGTGTACTGGTAATAAGTT
NS3-30nt-T20	AAATATCTTTAGGAGCACTAACAACTAATACCTGTAAGACTGTAT	GCGGTGTGTACATAGTTAACGGGGTCAGTGCCTTGAGTAACAGTG
NS3-30nt-T21	GATTAGAGCCGTCAATAGATAATACATTTGCTCCTCAGTAGCTTT	GAGCGTTTCTGCTGCCCCGTATAAACAGTTAATGCCCCCTGCCTA
NS3-30nt-T22	AGGATTTAGAAGTATTAGACTTTACAAACAGGTCTAGGTTTACCA	ACTTCCCATGAAAGATTTCGGAACCTATTATTCTGAAACATGAAA
NS3-30nt-T23	ATTCGACAACTCGTATTAAATCCTTTGCCCACTATGCGAGCAGAA	GGGTAGTAGAGAGCTGTATTAAGAGGCTGAGACTCCTCAAGAGAA
NS3-30nt-T24	GAACGTTATTAATTTTAAAAGTTTGAGTAACTGCTGTCGTCTCAG	GCAATGCATTTACAGGGATTAGGATTAGCGGGGTTTTGCTCAGTA
**Nanoswitch 4 30 nt targets**		
NS4-30nt-T1	TCTGTCCATCACGCAAATTAACCGTTGTAGTTGAGCAGGGTCGCC	AATGTACACATAGTGTCAACCGATTGAGGGAGGGAAGGTAAATAT
NS4-30nt-T2	CAATACTTCTTTGATTAGTAATAACATCACCCGAGGAACATGTCT	GGACCTATAGTTTTCTGACGGAAATTATTCATTAAAGGTGAATTA
NS4-30nt-T3	TTGCCTGAGTAGAAGAACTCAAACTATCGGCTTTTCTCCAAGCAG	GGTTACGTGTAAGGATCACCGTCACCGACTTGAGCCATTTGGGAA
NS4-30nt-T4	CCTTGCTGGTAATATCCAGAACAATATTACCACTGAGGTGTGTAG	GTGCCTGTGTAGGATTTAGAGCCAGCAAAATCACCAGTAGCACCA
NS4-30nt-T5	CGCCAGCCATTGCAACAGGAAAAACGCTCACATGTCCTTAGGTAT	GCCAGGTATGTCAACTTACCATTAGCAAGGCCGGAAACGTCACCA
NS4-30nt-T6	TGGAAATACCTACATTTTGACGCTCAATCGTTCGCGGGTGATAAA	CATGTTAGGGTAACCATGAAACCATCGATAGCAGCACCGTAATCA
NS4-30nt-T7	TCTGAAATGGATTATTTACATTGGCAGATTAACCTGTAGGTACAG	CAACTAGGTTAACACGTAGCGACAGAATCAAGTTTGCCTTTAGCG
NS4-30nt-T8	CACCAGTCACACGACCAGTAATAAAAGGGATAAATTGATCTCCAG	GCGGTGGTTTAGCACTCAGACTGTAGCGCGTTTTCATCGGCATTT
NS4-30nt-T9	CATTCTGGCCAACAGAGATAGAACCCTTCTACATAGACAACAGGT	GCGCTCAGGTCCTATTCGGTCATAGCCCCCTTATTAGCGTTTGCC
NS4-30nt-T10	GACCTGAAAGCGTAAGAATACGTGGCACAGCATGAGACTCACATG	GACTGTCAGAGTAATATCTTTTCATAATCAAAATCACCGGAACCA
NS4-30nt-T11	ACAATATTTTTGAATGGCTATTAGTCTTTAGCCAGCTGAGATCAT	CATGTTATAAGCATCGAGCCACCACCGGAACCGCCTCCCTCAGAG
NS4-30nt-T12	ATGCGCGAACTGATAGCCCTAAAACATCGCACTGGTACTTCACCC	TGTTGTCCATCAAAGCCGCCACCCTCAGAACCGCCACCCTCAGAG
NS4-30nt-T13	CATTAAAAATACCGAACGAACCACCAGCAGAGCATCTCTTTTGTA	GTCCCAGATCACAGTCCACCACCCTCAGAGCCGCCACCAGAACCA
NS4-30nt-T14	AAGATAAAACAGAGGTGAGGCGGTCAGTATCCCGGTTGCCACGCT	TGACTAGATTGTAATCCACCAGAGCCGCCGCCAGCATTGACAGGA
NS4-30nt-T15	TAACACCGCCTGCAACAGTGCCACGCTGAGCATTAAGATCTGAAT	CGACAAGCAGCGTACGGTTGAGGCAGGTCAGACGATTGGCCTTGA
NS4-30nt-T16	AGCCAGCAGCAAATGAAAAATCTAAAGCATCACGGAACCTCCAAG	AGCTAGCTTTTGTTGTATTCACAAACAAATAAATCCTCATTAAAG
NS4-30nt-T17	CACCTTGCTGAACCTCAAATATCAAACCCTACGTGTGAAAGAATT	AGTGTATGCAGGGGGCCAGAATGGAAAGCGCAGTCTCTGAATTTA
NS4-30nt-T18	CAATCAATATCTGGTCAGTTGGCAAATCAAGGTTATCAAACCTCT	TAGTACCATTGGTCCCCGTTCCAGTAAGCGTCATACATGGCTTTT
NS4-30nt-T19	CAGTTGAAAGGAATTGAGGAAGGTTATCTAAACAATAAGTAGGGA	CTGGGTCTTCGAATCGATGATACAGGAGTGTACTGGTAATAAGTT
NS4-30nt-T20	AAATATCTTTAGGAGCACTAACAACTAATACTGAGAGAGGGTCAA	GTGCACAGTCTACAGTTAACGGGGTCAGTGCCTTGAGTAACAGTG
NS4-30nt-T21	GATTAGAGCCGTCAATAGATAATACATTTGCCATTACAAGGTGTG	CTACCGGCCTGATAGCCCGTATAAACAGTTAATGCCCCCTGCCTA
NS4-30nt-T22	AGGATTTAGAAGTATTAGACTTTACAAACACAGTTGCTGGTGCAT	GTAGAAGTTCAAAAGTTTCGGAACCTATTATTCTGAAACATGAAA
NS4-30nt-T23	ATTCGACAACTCGTATTAAATCCTTTGCCCAGCAACCTGGTTAGA	AGTATTTGTTCCTGGGTATTAAGAGGCTGAGACTCCTCAAGAGAA
NS4-30nt-T24	GAACGTTATTAATTTTAAAAGTTTGAGTAAACGCCAAGTAGGAGT	AAGTTGATCTGCATGGGATTAGGATTAGCGGGGTTTTGCTCAGTA
**Nanoswitch 5 30 nt targets**		
NS5-30nt-T1	TCTGTCCATCACGCAAATTAACCGTTGTAGAGTCTGAGTCTGATA	ACTAGCGCATATACCTCAACCGATTGAGGGAGGGAAGGTAAATAT
NS5-30nt-T2	CAATACTTCTTTGATTAGTAATAACATCACGTGACATAGTGTAGG	CAATGATGGATTGACTGACGGAAATTATTCATTAAAGGTGAATTA
NS5-30nt-T3	TTGCCTGAGTAGAAGAACTCAAACTATCGGCCTCTTGCTTGGTTT	TGATGGATCTGGTAATCACCGTCACCGACTTGAGCCATTTGGGAA
NS5-30nt-T4	CCTTGCTGGTAATATCCAGAACAATATTACGCTTGTGCATTTTGG	TTGACCACATCTTGATTAGAGCCAGCAAAATCACCAGTAGCACCA
NS5-30nt-T5	CGCCAGCCATTGCAACAGGAAAAACGCTCACTCCTCCTTGAATGA	GTCTAATTCAGGTTGTTACCATTAGCAAGGCCGGAAACGTCACCA
NS5-30nt-T6	TGGAAATACCTACATTTTGACGCTCAATCGGAGTGAGGCTTGTAT	CGGTATCGTTGCAGTATGAAACCATCGATAGCAGCACCGTAATCA
NS5-30nt-T7	TCTGAAATGGATTATTTACATTGGCAGATTAGCAGCAACGAGCAA	AAGGTGTGAGTAAACGTAGCGACAGAATCAAGTTTGCCTTTAGCG
NS5-30nt-T8	CACCAGTCACACGACCAGTAATAAAAGGGAAGGCACGCTAGTAGT	CGTCGTCGGTTCATCTCAGACTGTAGCGCGTTTTCATCGGCATTT

NS5-30nt-T9	CATTCTGGCCAACAGAGATAGAACCCTTCTTTCTAGAAGCGGTCT	GGTCAGAATAGTGCCTCGGTCATAGCCCCCTTATTAGCGTTTGCC
NS5-30nt-T10	GACCTGAAAGCGTAAGAATACGTGGCACAGTGTCCAGCAATACGA	AGATGTCCACGAAGGATCTTTTCATAATCAAAATCACCGGAACCA
NS5-30nt-T11	ACAATATTTTTGAATGGCTATTAGTCTTTATAGCAACAGTGATTT	CTTTAGGCAGGTCCTGAGCCACCACCGGAACCGCCTCCCTCAGAG
NS5-30nt-T12	ATGCGCGAACTGATAGCCCTAAAACATCGCCGTCAGGACAAGCAA	AAGCAAATTGAGTGCCCGCCACCCTCAGAACCGCCACCCTCAGAG
NS5-30nt-T13	CATTAAAAATACCGAACGAACCACCAGCAGAGTGAATAGGACACG	GGTCATCAACTACATCCACCACCCTCAGAGCCGCCACCAGAACCA
NS5-30nt-T14	AAGATAAAACAGAGGTGAGGCGGTCAGTATACCTTGGGGCCGACG	TTGTTTTGATCGCGCCCACCAGAGCCGCCGCCAGCATTGACAGGA
NS5-30nt-T15	TAACACCGCCTGCAACAGTGCCACGCTGAGCTTCCTTGCCATGTT	GAGTGAGAGCGGTGAGGTTGAGGCAGGTCAGACGATTGGCCTTGA
NS5-30nt-T16	AGCCAGCAGCAAATGAAAAATCTAAAGCATGGTAGTAGCCAATTT	GGTCATCTGGACTGCTATTCACAAACAAATAAATCCTCATTAAAG
NS5-30nt-T17	CACCTTGCTGAACCTCAAATATCAAACCCTTGTTAGCACCATAGG	GAAGTCCAGCTTCTGCCAGAATGGAAAGCGCAGTCTCTGAATTTA
NS5-30nt-T18	CAATCAATATCTGGTCAGTTGGCAAATCAACAGGATTGCGGGTGC	CAATGTGATCTTTTGCCGTTCCAGTAAGCGTCATACATGGCTTTT
NS5-30nt-T19	CAGTTGAAAGGAATTGAGGAAGGTTATCTATTGCGACTACGTGAT	GAGGAACGAGAAGAGGATGATACAGGAGTGTACTGGTAATAAGTT
NS5-30nt-T20	AAATATCTTTAGGAGCACTAACAACTAATACCTTGGGTTTGTTCT	GGACCACGTCTGCCGTTAACGGGGTCAGTGCCTTGAGTAACAGTG
NS5-30nt-T21	GATTAGAGCCGTCAATAGATAATACATTTGGTTCCTTGTCTGATT	AGTTCCTGGTCCCCACCCGTATAAACAGTTAATGCCCCCTGCCTA
NS5-30nt-T22	AGGATTTAGAAGTATTAGACTTTACAAACAGTTTCTTCTGTCTCT	GCGGTAAGGCTTGAGTTTCGGAACCTATTATTCTGAAACATGAAA
NS5-30nt-T23	ATTCGACAACTCGTATTAAATCCTTTGCCCGTCTGCATGAGTTTA	GGCCTGAGTTGAGTCGTATTAAGAGGCTGAGACTCCTCAAGAGAA
NS5-30nt-T24	GAACGTTATTAATTTTAAAAGTTTGAGTAAAGGCAGCTCTCCCTA	GCATTGTTCACTGTAGGATTAGGATTAGCGGGGTTTTGCTCAGTA

Detector 1 sequence (nt 1-30 hybridize M13, 31-60 hybridize with target)

**Nanoswitch 1 60 nt targets**	
NS1-60nt-T1	TCTGTCCATCACGCAAATTAACCGTTGTAGACAGATTTTAAAGTTCGTTTAGAGAACAGA
NS1-60nt-T2	CAATACTTCTTTGATTAGTAATAACATCACTGTAAAACAGGCAAACTGAGTTGGACGTGT
NS1-60nt-T3	TTGCCTGAGTAGAAGAACTCAAACTATCGGCCAGTTTTCTTGAAAATCTTCATAAGGATC
NS1-60nt-T4	CCTTGCTGGTAATATCCAGAACAATATTACCAACCCTTGGTTGAATAGTCTTGATTATGG
NS1-60nt-T5	CGCCAGCCATTGCAACAGGAAAAACGCTCAACTTCTGAATTGTGACATGCTGGACAATAA
NS1-60nt-T6	TGGAAATACCTACATTTTGACGCTCAATCGAACAATATTGATGTTGACTTTCTCTTTTTG
NS1-60nt-T7	TCTGAAATGGATTATTTACATTGGCAGATTTTTCTGTTCACCAATATTCCAGGCACCTTT
NS1-60nt-T8	CACCAGTCACACGACCAGTAATAAAAGGGACTTCAAGCCAATCAAGGACGGGTTTGAGTT
NS1-60nt-T9	CATTCTGGCCAACAGAGATAGAACCCTTCTGATAATTTCTTTTGGGGCTTTTAGAGGCAT
NS1-60nt-T10	GACCTGAAAGCGTAAGAATACGTGGCACAGACTTCTCATTAAGTACTTTATCAATCCTTT
NS1-60nt-T11	ACAATATTTTTGAATGGCTATTAGTCTTTACTCATATTGAGTTGATGGCTCAAACTCTTC
NS1-60nt-T12	ATGCGCGAACTGATAGCCCTAAAACATCGCGGTTGAACCTCAACAATTGTTTGAATAGTA
NS1-60nt-T13	CATTAAAAATACCGAACGAACCACCAGCAGTTTAAGTGGTCCATTAGTAGCTATGTAATC
NS1-60nt-T14	AAGATAAAACAGAGGTGAGGCGGTCAGTATAATACCAGCTGATAATAATGGTGCAAGTAG
NS1-60nt-T15	TAACACCGCCTGCAACAGTGCCACGCTGAGCCTCTTTAGGAATCTCAGCGATCTTTTGTT
NS1-60nt-T16	AGCCAGCAGCAAATGAAAAATCTAAAGCATATCTGGATGAAGATTGCCATTAATGTCAAT
NS1-60nt-T17	CACCTTGCTGAACCTCAAATATCAAACCCTCGGGTAAGTGGTTATATAATTGTCTGTTGG
NS1-60nt-T18	CAATCAATATCTGGTCAGTTGGCAAATCAACCTCTTGTATTTTAATACCCTTATATTTAC
NS1-60nt-T19	CAGTTGAAAGGAATTGAGGAAGGTTATCTAAGCAAGTGAGATGGTTTCAATAAAATGTTC
NS1-60nt-T20	AAATATCTTTAGGAGCACTAACAACTAATAATAAAATGTTTTACCTTCATGTGAATTATG
NS1-60nt-T21	GATTAGAGCCGTCAATAGATAATACATTTGTGTAATAAGCATCTTGTAGAGCAGGTGGAT
NS1-60nt-T22	AGGATTTAGAAGTATTAGACTTTACAAACACTTAAGGGTTGTCTGCTGTTGTCCACAAGT
NS1-60nt-T23	ATTCGACAACTCGTATTAAATCCTTTGCCCCCGTCTATGCAATACAAAGTTTCTTTAGAA
NS1-60nt-T24	GAACGTTATTAATTTTAAAAGTTTGAGTAATTGCGTTTGGATATGGTTGGTTTGGTACAA
**Nanoswitch 2 60 nt targets**	
NS2-60nt-T1	TCTGTCCATCACGCAAATTAACCGTTGTAGATAGGTTTATGTAACAATTTAGCTCCTTTC
NS2-60nt-T2	CAATACTTCTTTGATTAGTAATAACATCACATTAGATCTGTGTGGCCAACCTCTTCTGTA
NS2-60nt-T3	TTGCCTGAGTAGAAGAACTCAAACTATCGGGTACACAATTGTAGCAATAAAGTAAAGAAA
NS2-60nt-T4	CCTTGCTGGTAATATCCAGAACAATATTACGATTAAAGAACCTAGGCAAACACTTAATAG
NS2-60nt-T5	CGCCAGCCATTGCAACAGGAAAAACGCTCACATTTAAAAGATGAAATGGTAATTTGTATA
NS2-60nt-T6	TGGAAATACCTACATTTTGACGCTCAATCGCATACATAATAAAATGATGCAAAGAAGATG
NS2-60nt-T7	TCTGAAATGGATTATTTACATTGGCAGATTCTGTAACACTATCAACGATGTAAGAAGACT
NS2-60nt-T8	CACCAGTCACACGACCAGTAATAAAAGGGAGCTGATTTTGCAGATGATTCTTCACATTTT
NS2-60nt-T9	CATTCTGGCCAACAGAGATAGAACCCTTCTAGTTGCAACTAGTGTTTTGAGTTTTTCCAT
NS2-60nt-T10	GACCTGAAAGCGTAAGAATACGTGGCACAGCCAAGGTCACGGGGTGTCATGTTTTCAACT
NS2-60nt-T11	ACAATATTTTTGAATGGCTATTAGTCTTTATGTTTAGACATGACATGAACAGGTGTTATT
NS2-60nt-T12	ATGCGCGAACTGATAGCCCTAAAACATCGCACAAGCTGATGTTGCAAAGTCAGTGTACTC
NS2-60nt-T13	CATTAAAAATACCGAACGAACCACCAGCAGTCAAGGTAGGTGTTAGGAAATTGAATAATA
NS2-60nt-T14	AAGATAAAACAGAGGTGAGGCGGTCAGTATGTTAAACAGAGTACAGTGAATGACATAAGG
NS2-60nt-T15	TAACACCGCCTGCAACAGTGCCACGCTGAGGGAAATACAAATGATATAAGCAATTGTTAT
NS2-60nt-T16	AGCCAGCAGCAAATGAAAAATCTAAAGCATCTGAGGTGATAGAGGTTTGTGGTGGTTGGT
NS2-60nt-T17	CACCTTGCTGAACCTCAAATATCAAACCCTTCTGTCCTGGTTGAATGCGAACAAACTTAT
NS2-60nt-T18	CAATCAATATCTGGTCAGTTGGCAAATCAAGTAACAAAAAGAGACACAGTCATAATCTAT
NS2-60nt-T19	CAGTTGAAAGGAATTGAGGAAGGTTATCTAAAAGAGGTCCTAGTATGTCAACATGGTCTT
NS2-60nt-T20	AAATATCTTTAGGAGCACTAACAACTAATACCATAGCAAAAGGTAAAAAGGCATTTTCAT

NS2-60nt-T21 GATTAGAGCCGTCAATAGATAATACATTTGTCATAAGGATTAGTAACACTACAGCTGATG
NS2-60nt-T22 AGGATTTAGAAGTATTAGACTTTACAAACATGAAGAAAATAGGGCAATACTCAACACACA
NS2-60nt-T23 ATTCGACAACTCGTATTAAATCCTTTGCCCGTTGCAAAACTGAGAGTAAGACTACTGATG
NS2-60nt-T24 GAACGTTATTAATTTTAAAAGTTTGAGTAAGCAAAAGCTGCATATGATGGAAGGGAACTA

Nanoswitch 3 60 nt targets

NS3-60nt-T1 TCTGTCCATCACGCAAATTAACCGTTGTAGATGTTGTTGAGTGCATCATTATCCAACTTT
NS3-60nt-T2 CAATACTTCTTTGATTAGTAATAACATCACCTTAAAGCTGTTACAATAAGAGGCCATGCT
NS3-60nt-T3 TTGCCTGAGTAGAAGAACTCAAACTATCGGGTATAGATAGTACCAGTTCCATCACTCTTA
NS3-60nt-T4 CCTTGCTGGTAATATCCAGAACAATATTACTAAGCTTTAGCAGCATCTACAGCAAAAGCA
NS3-60nt-T5 CGCCAGCCATTGCAACAGGAAAAACGCTCACAGACTGTGTTTTTAAGTGTAAAACCCACA
NS3-60nt-T6 TGGAAATACCTACATTTTGACGCTCAATCGCCTTTTCTTGGAAGCGACAACAATTAGTTT
NS3-60nt-T7 TCTGAAATGGATTATTTACATTGGCAGATTTAAAGCATAGACGAGGTCTGCCATTGTGTA
NS3-60nt-T8 CACCAGTCACACGACCAGTAATAAAAGGGAGTATACGCGTAATATATCTGGGTTTTCTAC
NS3-60nt-T9 CATTCTGGCCAACAGAGATAGAACCCTTCTCAAGGTTAATATAGGCATTAACAATGAATA
NS3-60nt-T10 GACCTGAAAGCGTAAGAATACGTGGCACAGTGGTCCAAAACTTGTAGGTGGGAACACTGT
NS3-60nt-T11 ACAATATTTTTGAATGGCTATTAGTCTTTACATAGAAGTCTTTGTTAAAATTACCGGGTT
NS3-60nt-T12 ATGCGCGAACTGATAGCCCTAAAACATCGCAATGGAAAACCAGCTGATTTGTCTAGGTTG
NS3-60nt-T13 CATTAAAAATACCGAACGAACCACCAGCAGCGAGCTCTATTCTTTGCACTAATGGCATAC
NS3-60nt-T14 AAGATAAAACAGAGGTGAGGCGGTCAGTATCACGTTGTATGTTTGCGAGCAAGAACAAGT
NS3-60nt-T15 TAACACCGCCTGCAACAGTGCCACGCTGAGTATTGAAACACACAACAGCATCGTCAGAGA
NS3-60nt-T16 AGCCAGCAGCAAATGAAAAATCTAAAGCATACAAAACAGCCGGCCCCTAGGATTCTTGAT
NS3-60nt-T17 CACCTTGCTGAACCTCAAATATCAAACCCTTGTCTAACATGTGTCCTGTTAACTCATCAT
NS3-60nt-T18 CAATCAATATCTGGTCAGTTGGCAAATCAACGGATTAACAGACAAGACTAATTTATGTGA
NS3-60nt-T19 CAGTTGAAAGGAATTGAGGAAGGTTATCTAGCTTGAGTCTTTCAGTACAGGTGTTAGCTA
NS3-60nt-T20 AAATATCTTTAGGAGCACTAACAACTAATATGTTTTTAGTTACACGATAACCAGTAAAGA
NS3-60nt-T21 GATTAGAGCCGTCAATAGATAATACATTTGCAAGCTGTATACACTATGCGAGCAGAAGGG
NS3-60nt-T22 AGGATTTAGAAGTATTAGACTTTACAAACAATTGAAATATTCTGGTTCTAGTGTGCCCTT
NS3-60nt-T23 ATTCGACAACTCGTATTAAATCCTTTGCCCGTGAATTATAAGGTGAAATAAAGACAGCTT
NS3-60nt-T24 GAACGTTATTAATTTTAAAAGTTTGAGTAATACTACAATCTTTAAAGAGTCCTGTTACAT

Nanoswitch 4 60 nt targets

NS4-60nt-T1 TCTGTCCATCACGCAAATTAACCGTTGTAGGCTTCTTCGCGGGTGATAAACATGTTAGGG
NS4-60nt-T2 CAATACTTCTTTGATTAGTAATAACATCACAAGTCCTTTGTACATAAGTGGTATGAGGTG
NS4-60nt-T3 TTGCCTGAGTAGAAGAACTCAAACTATCGGTCACATAGACAACAGGTGCGCTCAGGTCCT
NS4-60nt-T4 CCTTGCTGGTAATATCCAGAACAATATTACTTAATCTTCAGTTCATCACCAATTATAGGA
NS4-60nt-T5 CGCCAGCCATTGCAACAGGAAAAACGCTCATGAATTTGTCAGAATGTGTGGCATAAGAAT
NS4-60nt-T6 TGGAAATACCTACATTTTGACGCTCAATCGTCACATGGACTGTCAGAGTAATAGAAAAAT
NS4-60nt-T7 TCTGAAATGGATTATTTACATTGGCAGATTGTCTTGTAAAAGTGTTCCAGAGGTTATAAG
NS4-60nt-T8 CACCAGTCACACGACCAGTAATAAAAGGGACAAAAAAGACAGTGAGTGGTGCACAAATCG
NS4-60nt-T9 CATTCTGGCCAACAGAGATAGAACCCTTCTGTTTAAATTCTTGTAAATTTCTACTCTGAG
NS4-60nt-T10 GACCTGAAAGCGTAAGAATACGTGGCACAGGCGCATCTGTTATGAAATAGTTTTTAACTG
NS4-60nt-T11 ACAATATTTTTGAATGGCTATTAGTCTTTACCACGCTTGACTAGATTGTAATTTTGGGTA
NS4-60nt-T12 ATGCGCGAACTGATAGCCCTAAAACATCGCCCAAAATGTATAACTCTCATATTATAGGGT
NS4-60nt-T13 CATTAAAAATACCGAACGAACCACCAGCAGCTTTAGAGTCATTTTCTTTTGTAACATTTT
NS4-60nt-T14 AAGATAAAACAGAGGTGAGGCGGTCAGTATTTTGTATTCCTCCAAAATATGTAATTTGCA
NS4-60nt-T15 TAACACCGCCTGCAACAGTGCCACGCTGAGCCACGTGTGAAAGAATTAGTGTATGCAGGG
NS4-60nt-T16 AGCCAGCAGCAAATGAAAAATCTAAAGCATCAACATTAGTAGCGTTATTAACAATAAGTA
NS4-60nt-T17 CACCTTGCTGAACCTCAAATATCAAACCCTCACTAAATTAATAGGCGTGTGCTTAGAATA
NS4-60nt-T18 CAATCAATATCTGGTCAGTTGGCAAATCAAACAGCATCTGTAATGGTTCCATTTTCATTA
NS4-60nt-T19 CAGTTGAAAGGAATTGAGGAAGGTTATCTAGCATAGACATTAGTAAAGCAGAGATCATTT
NS4-60nt-T20 AAATATCTTTAGGAGCACTAACAACTAATACCAACACCATTAGTGGGTTGGAAACCATAT
NS4-60nt-T21 GATTAGAGCCGTCAATAGATAATACATTTGTCTGTGGATCACGGACAGCATCAGTAGTGT
NS4-60nt-T22 AGGATTTAGAAGTATTAGACTTTACAAACAGTTTGAAAAACATTAGAACCTGTAGAATAA
NS4-60nt-T23 ATTCGACAACTCGTATTAAATCCTTTGCCCGGTAGAATTTCTGTGGTAACACTAATAGTA
NS4-60nt-T24 GAACGTTATTAATTTTAAAAGTTTGAGTAAATCTTTAATTGGTGGTGTTTTGTAAATTTG

Nanoswitch 5 60 nt targets

NS5-60nt-T1 TCTGTCCATCACGCAAATTAACCGTTGTAGGAAGTGTATTGAGCAATCATTTCATCTGTG
NS5-60nt-T2 CAATACTTCTTTGATTAGTAATAACATCACACTTTGTCAAGACGTGAAAGGATATCATTT
NS5-60nt-T3 TTGCCTGAGTAGAAGAACTCAAACTATCGGTTTTCCATCATGACAAATGGCAGGAGCAGT
NS5-60nt-T4 CCTTGCTGGTAATATCCAGAACAATATTACTGAATGAGTCTAATTCAGGTTGCAAAGGAT
NS5-60nt-T5 CGCCAGCCATTGCAACAGGAAAAACGCTCAAAGTTCTTGGAGATCGATGAGAGATTCATT
NS5-60nt-T6 TGGAAATACCTACATTTTGACGCTCAATCGTCCTTGATTTCACCTTGCTTCAAAGTTACA
NS5-60nt-T7 TCTGAAATGGATTATTTACATTGGCAGATTCATAAAGATAGAGAAAAGGGGCTTCAAGGC
NS5-60nt-T8 CACCAGTCACACGACCAGTAATAAAAGGGACAATAGTCGTAACAATTAGTATGCCAGCAA
NS5-60nt-T9 CATTCTGGCCAACAGAGATAGAACCCTTCTACAATTTTATTGTAGATGAAGAAGGTAACA
NS5-60nt-T10 GACCTGAAAGCGTAAGAATACGTGGCACAGTAGTGTAACTAGCAAGAATACCACGAAAGC
NS5-60nt-T11 ACAATATTTTTGAATGGCTATTAGTCTTTACGTTGGAATCTGCCATGGCTAAAATTAAAG

NS5-60nt-T12	ATGCGCGAACTGATAGCCCTAAAACATCGCCATGGAGTGGCACGTTGAGAAGAATGTTAG
NS5-60nt-T13	CATTAAAAATACCGAACGAACCACCAGCAGCCCAATTTGTAATAAGAAAGCGTTCGTGAT
NS5-60nt-T14	AAGATAAAACAGAGGTGAGGCGGTCAGTATCTCAGTTAGTGACTTAGATAAATTTTTAAT
NS5-60nt-T15	TAACACCGCCTGCAACAGTGCCACGCTGAGAATTGAGTGCTAAAGCAAGTCAGTGCAAAT
NS5-60nt-T16	AGCCAGCAGCAAATGAAAAATCTAAAGCATAAGATAATAAGCATAATTAAAACAAGGAAT
NS5-60nt-T17	CACCTTGCTGAACCTCAAATATCAAACCCTTAAAAGGTAAACAGGAAACTGTATAATTAC
NS5-60nt-T18	CAATCAATATCTGGTCAGTTGGCAAATCAACTTGCCATGTTGAGTGAGAGCGGTGAACCA
NS5-60nt-T19	CAGTTGAAAGGAATTGAGGAAGGTTATCTATAGAAATACCATCTTGGACTGAGATCTTTC
NS5-60nt-T20	AAATATCTTTAGGAGCACTAACAACTAATACCTGGAGTTGAATTTCTTGAACTGTTGCGA
NS5-60nt-T21	GATTAGAGCCGTCAATAGATAATACATTTGCAAAATTTCCTTGGGTTTGTTCTGGACCAC
NS5-60nt-T22	AGGATTTAGAAGTATTAGACTTTACAAACACTTCTTCTTTTTGTCCTTTTTAGGCTCTGT
NS5-60nt-T23	ATTCGACAACTCGTATTAAATCCTTTGCCCGAGAATTCATTCTGCACAAGAGTAGACTAT
NS5-60nt-T24	GAACGTTATTAATTTTAAAAGTTTGAGTAAGCTATTAAAATCACATGGGGATAGCACTAC

Detector 2 sequence (nt 1-30 hybridize with target, 31-60 hybridizing M13)

Nanoswitch 1 60 nt targets

NS1-60nt-T1	TCTACAAGAGATCGAAAGTTGGTTGGTTTGTCAACCGATTGAGGGAGGGAAGGTAAATAT
NS1-60nt-T2	GTTTTCTCGTTGAAACCAGGGACAAGGCTCTGACGGAAATTATTCATTAAAGGTGAATTA
NS1-60nt-T3	AGTGCCAAGCTCGTCGCCTAAGTCAAATGATCACCGTCACCGACTTGAGCCATTTGGGAA
NS1-60nt-T4	AATTTAAGGGAAATACAAAATTTGGACATTTTAGAGCCAGCAAAATCACCAGTAGCACCA
NS1-60nt-T5	ATTTTAACAACAGCATTTTGGGGTAAGTAATTACCATTAGCAAGGCCGGAAACGTCACCA
NS1-60nt-T6	GAGTATTTCAAGAAGGTTGTCATTAAGACCATGAAACCATCGATAGCAGCACCGTAATCA
NS1-60nt-T7	TTTAGCTTTTCCTTTTGTAACTTTAAAATTGTAGCGACAGAATCAAGTTTGCCTTTAGCG
NS1-60nt-T8	TTTCATAAACAGTGCCAAAGATGTTAGTTATCAGACTGTAGCGCGTTTTCATCGGCATTT
NS1-60nt-T9	GAGTAGGCCAGTTTCTTCTCTGGATTTAACTCGGTCATAGCCCCCTTATTAGCGTTTGCC
NS1-60nt-T10	CATCAAGTTCAAAAGTGATATTCACACTCTATCTTTTCATAATCAAAATCACCGGAACCA
NS1-60nt-T11	TTCTTCACAATCACCTTCTTCTTCATCCTCGAGCCACCACCGGAACCGCCTCCCTCAGAG
NS1-60nt-T12	GTTGTCTGATTGTCCTCACTGCCGTCTTGTCCGCCACCCTCAGAACCGCCACCCTCAGAG
NS1-60nt-T13	ATCAGATTCAACTTGCATGGCATTGTTAGTCCACCACCCTCAGAGCCGCCACCAGAACCA
NS1-60nt-T14	AACTTCGTGCTGATTAAAATTTTCATAAGCCCACCAGAGCCGCCGCCAGCATTGACAGGA
NS1-60nt-T15	CAACTTGCTTTTCACTCTTCATTTCCAAAAGGTTGAGGCAGGTCAGACGATTGGCCTTGA
NS1-60nt-T16	ATAAAGTAACAAGTTTTCTGTGAGGAACTTTATTCACAAACAAATAAATCCTCATTAAAG
NS1-60nt-T17	CACTTTTCTCAAAGCTTTCGCTAGCATTTCCCAGAATGGAAAGCGCAGTCTCTGAATTTA
NS1-60nt-T18	GCTGTATAGTTGAAACTATGGCTTTAGTTTCCGTTCCAGTAAGCGTCATACATGGCTTTT
NS1-60nt-T19	TTCAGGTGTTTTAGAAGAAGAAGTAAGATAGATGATACAGGAGTGTACTGGTAATAAGTT
NS1-60nt-T20	AGGTTTTATTTTAGTAACATCAGCTCCATCTTAACGGGGTCAGTGCCTTGAGTAACAGTG
NS1-60nt-T21	TAAACTTCAACTCTATTTGTTGGAGTGTTACCCGTATAAACAGTTAATGCCCCCTGCCTA
NS1-60nt-T22	TTTACACACCACGTTCAAGACTCTTTTGCATTTCGGAACCTATTATTCTGAAACATGAAA
NS1-60nt-T23	GTTATATGTTTATAGTGACCACACTGGTAAGTATTAAGAGGCTGAGACTCCTCAAGAGAA
NS1-60nt-T24	GATCAATTGGTTGCTCTGTGAAATAAGAATGGATTAGGATTAGCGGGGTTTTGCTCAGTA

Nanoswitch 2 60 nt targets

NS2-60nt-T1	TTAAAAGAGGGTGTGTAGTGTTTATAATCATCAACCGATTGAGGGAGGGAAGGTAAATAT
NS2-60nt-T2	ATTTTTAAACTATTATTTGCTGGTTTAAGTTGACGGAAATTATTCATTAAAGGTGAATTA
NS2-60nt-T3	TAAGGCATATAATTAGTACAAACACGGTTTTCACCGTCACCGACTTGAGCCATTTGGGAA
NS2-60nt-T4	TAAAAACCAAATTATAATATTTATCAGTTTTTAGAGCCAGCAAAATCACCAGTAGCACCA
NS2-60nt-T5	GTTTCTAAAGAAGGATAGGTGTCTAAAGAATTACCATTAGCAAGGCCGGAAACGTCACCA
NS2-60nt-T6	TACATTCTAACCATAGCTGAAATCGGGGCCATGAAACCATCGATAGCAGCACCGTAATCA
NS2-60nt-T7	GGTCAGTAGGATTTATTGGTCTTTTAAACTGTAGCGACAGAATCAAGTTTGCCTTTAGCG
NS2-60nt-T8	GATTTACCATCAAAAACTATAACATTAATATCAGACTGTAGCGCGTTTTCATCGGCATTT
NS2-60nt-T9	TGGTACGTTAAAAGTTGATGAAAACGTATTTCGGTCATAGCCCCCTTATTAGCGTTTGCC
NS2-60nt-T10	TTGTTATAGGTGAGCATATAGTTATTACAAATCTTTTCATAATCAAAATCACCGGAACCA
NS2-60nt-T11	AAATAGAAAATAGCAGCAACAAAAAGGAACGAGCCACCACCGGAACCGCCTCCCTCAGAG
NS2-60nt-T12	TATAAGTTTTGATGGTGTGTAACAGATGTTCCGCCACCCTCAGAACCGCCACCCTCAGAG
NS2-60nt-T13	GAGCCATCCATGAGCACATAACGTGTGTCACCACCACCCTCAGAGCCGCCACCAGAACCA
NS2-60nt-T14	AATAGTAAAGTATTAAAGGCAACTACATGACCACCAGAGCCGCCGCCAGCATTGACAGGA
NS2-60nt-T15	CCAGAAAGGTACTAAAGGTGTGAACATAACGGTTGAGGCAGGTCAGACGATTGGCCTTGA
NS2-60nt-T16	AAAGAACATCAGAACCTGAGTTACTGAAGTTATTCACAAACAAATAAATCCTCATTAAAG
NS2-60nt-T17	ACTTAGGTGTCTTAGGATTGGCTGTATCAACCAGAATGGAAAGCGCAGTCTCTGAATTTA
NS2-60nt-T18	GTTAAAACCAACACTACCACATGAACCATTCCGTTCCAGTAAGCGTCATACATGGCTTTT
NS2-60nt-T19	GTGTTAGAGGTTCATAATTGTACTTCATAGGATGATACAGGAGTGTACTGGTAATAAGTT
NS2-60nt-T20	ACAAAAAAAAGAACAAAGACCATTGAGTACTTAACGGGGTCAGTGCCTTGAGTAACAGTG
NS2-60nt-T21	CATACATAACACAGTCTTTTAGCTTAAAACCCCGTATAAACAGTTAATGCCCCCTGCCTA
NS2-60nt-T22	TAAAAACAATACCTCTGGCCAAAAACATGATTTCGGAACCTATTATTCTGAAACATGAAA
NS2-60nt-T23	TGCACTTTACATCTGACATTTTAGACTGTAGTATTAAGAGGCTGAGACTCCTCAAGAGAA
NS2-60nt-T24	AACTCTGAGGCTATAGCTTGTAAGGTTGCCGGATTAGGATTAGCGGGGTTTTGCTCAGTA

Nanoswitch 3 60 nt targets

NS3-60nt-T1	CTAAGCATAGTGAAAAGCATTGTCTGCATATCAACCGATTGAGGGAGGGAAGGTAAATAT
NS3-60nt-T2	AAATTAGGTGAATTGTCCATACTAATTTCATGACGGAAATTATTCATTAAAGGTGAATTA
NS3-60nt-T3	GGGAATCTAGCCCATTTCAAATCCTGTAAATCACCGTCACCGACTTGAGCCATTTGGGAA
NS3-60nt-T4	CAGAAAGATAATACAGTTGAATTGGCAGGCTTAGAGCCAGCAAAATCACCAGTAGCACCA
NS3-60nt-T5	GGGTCATTAGCACAAGTTGTAGGTATTTGTTTACCATTAGCAAGGCCGGAAACGTCACCA
NS3-60nt-T6	TTAGGAATTTAGCAAAACCAGCTACTTTATATGAAACCATCGATAGCAGCACCGTAATCA
NS3-60nt-T7	TTTAGTAAGACGTTGACGTGATATATGTGGGTAGCGACAGAATCAAGTTTGCCTTTAGCG
NS3-60nt-T8	AAAATCATACCAGTCCTTTTTATTGAAATATCAGACTGTAGCGCGTTTTCATCGGCATTT
NS3-60nt-T9	ATAAGAATCTACAACAGGAACTCCACTACCTCGGTCATAGCCCCCTTATTAGCGTTTGCC
NS3-60nt-T10	AGAGAATAAAACATTAAAGTTTGCACAATGATCTTTTCATAATCAAAATCACCGGAACCA
NS3-60nt-T11	TGACAGTTTGAAAAGCAACATTGTTAGTAAGAGCCACCACCGGAACCGCCTCCCTCAGAG
NS3-60nt-T12	TTGACGATGACTTGGTTAGCATTAATACAGCCGCCACCCTCAGAACCGCCACCCTCAGAG
NS3-60nt-T13	TTAAGATTCATTTGAGTTATAGTAGGGATGCCACCACCCTCAGAGCCGCCACCAGAACCA
NS3-60nt-T14	GAGGCCATAATTCTAAGCATGTTAGGCATGCCACCAGAGCCGCCGCCAGCATTGACAGGA
NS3-60nt-T15	GTATCATCATTGAGAAATGTTTACGCAAATGGTTGAGGCAGGTCAGACGATTGGCCTTGA
NS3-60nt-T16	GGATCTGGGTAAGGAAGGTACACATAATCATATTCACAAACAAATAAATCCTCATTAAAG
NS3-60nt-T17	GTAGCTTTCTTATGTATTGTAAGTACAAATCCAGAATGGAAAGCGCAGTCTCTGAATTTA
NS3-60nt-T18	TGTTGATATGACATGGTCGTAACAGCATTTCCGTTCCAGTAAGCGTCATACATGGCTTTT
NS3-60nt-T19	AAATGTAATCACCAGCATTTGTCCAGTCACGATGATACAGGAGTGTACTGGTAATAAGTT
NS3-60nt-T20	CATAATTTCGGTTAAGTGGTGGTCTAGGTTTTAACGGGGTCAGTGCCTTGAGTAACAGTG
NS3-60nt-T21	TAGTAGAGAGCTAGGCCAATAGCAAAATGACCCGTATAAACAGTTAATGCCCCCTGCCTA
NS3-60nt-T22	AGTTAGCAATGTGCGTGGTGCAGGTAATTGTTTCGGAACCTATTATTCTGAAACATGAAA
NS3-60nt-T23	TTCTCCAAGCAGGGTTACGTGTAAGGAATTGTATTAAGAGGCTGAGACTCCTCAAGAGAA
NS3-60nt-T24	TTTCAGCTTGTAAAGTTGCCACATTCCTACGGATTAGGATTAGCGGGGTTTTGCTCAGTA

Nanoswitch 4 60 nt targets

NS4-60nt-T1	TAACCATTAACTTGATAATTCATTTTAAAATCAACCGATTGAGGGAGGGAAGGTAAATAT
NS4-60nt-T2	TTTAAATTGATCTCCAGGCGGTGGTTTAGCTGACGGAAATTATTCATTAAAGGTGAATTA
NS4-60nt-T3	ATTTTCACAAAATACTTCATAGATGTCAACTCACCGTCACCGACTTGAGCCATTTGGGAA
NS4-60nt-T4	TATTCAATAGTCCAGTCAACACGCTTAACATTAGAGCCAGCAAAATCACCAGTAGCACCA
NS4-60nt-T5	AGAATAATTCTTCTATTTTATAAGCTTTGTTTACCATTAGCAAGGCCGGAAACGTCACCA
NS4-60nt-T6	GGTAATTGTTTTAAATTAACAAAAGCACTTATGAAACCATCGATAGCAGCACCGTAATCA
NS4-60nt-T7	TATCAAATTGTTTGTAAACCCACAAGCTAAGTAGCGACAGAATCAAGTTTGCCTTTAGCG
NS4-60nt-T8	TTTCAGTTGGTTTCTTGGCTATGTCAGTCATCAGACTGTAGCGCGTTTTCATCGGCATTT
NS4-60nt-T9	TAAAGTAAGTTTCAGGTAATTGTTGGACAATCGGTCATAGCCCCCTTATTAGCGTTTGCC
NS4-60nt-T10	TACTGTCCATAGGAATAAAATCTTCTAATTATCTTTTCATAATCAAAATCACCGGAACCA
NS4-60nt-T11	AAATGTTTCTACATGGCCATCTTTACACCAGAGCCACCACCGGAACCGCCTCCCTCAGAG
NS4-60nt-T12	ACAGCTAATGTTAATGTGTTTAAATATTGACCGCCACCCTCAGAACCGCCACCCTCAGAG
NS4-60nt-T13	TAGTCTTAGGGTCGTACATATCACTAATAACCACCACCCTCAGAGCCGCCACCAGAACCA
NS4-60nt-T14	TGCATGACATAACCATCTATTTGTTCGCGTCCACCAGAGCCGCCGCCAGCATTGACAGGA
NS4-60nt-T15	GGTAATTGAGTTCTGGTTGTAAGATTAACAGGTTGAGGCAGGTCAGACGATTGGCCTTGA
NS4-60nt-T16	GGGACTGGGTCTTCGAATCTAAAGTAGTACTATTCACAAACAAATAAATCCTCATTAAAG
NS4-60nt-T17	TATTTTAAAATAACCATCAATATTCTTAAACCAGAATGGAAAGCGCAGTCTCTGAATTTA
NS4-60nt-T18	TATTTTAATAGAAAAGTCCTAGGTTGAAGACCGTTCCAGTAAGCGTCATACATGGCTTTT
NS4-60nt-T19	AATTTAGTAGGAGACACTCCATAACACTTAGATGATACAGGAGTGTACTGGTAATAAGTT
NS4-60nt-T20	GATTGTAAAGGAAAGTAACAATTAAAACCTTTAACGGGGTCAGTGCCTTGAGTAACAGTG
NS4-60nt-T21	CAGCAATGTCTCTGCCAAATTGTTGGAAAGCCCGTATAAACAGTTAATGCCCCCTGCCTA
NS4-60nt-T22	ACACGCCAAGTAGGAGTAAGTTGATCTGCATTTCGGAACCTATTATTCTGAAACATGAAA
NS4-60nt-T23	AAATTTGTGGGTATGGCAATAGAGTTATTAGTATTAAGAGGCTGAGACTCCTCAAGAGAA
NS4-60nt-T24	TTTGACTTGTGCAAAAACTTCTTGGGTGTTGGATTAGGATTAGCGGGGTTTTGCTCAGTA

Nanoswitch 5 60 nt targets

NS5-60nt-T1	AGCAAAGGTGGCAAAACAGTAAGGCCGTTATCAACCGATTGAGGGAGGGAAGGTAAATAT
NS5-60nt-T2	AAAACACTTGAAATTGCACCAAAATTGGAGTGACGGAAATTATTCATTAAAGGTGAATTA
NS5-60nt-T3	TGTGAAGTTCTTTTCTTGTGCAGGGACATATCACCGTCACCGACTTGAGCCATTTGGGAA
NS5-60nt-T4	CATAAACTGTGTTGTTGACAATTCCTATTATTAGAGCCAGCAAAATCACCAGTAGCACCA
NS5-60nt-T5	TAAATTCTTGGCAACCTCATTGAGGCGGTCTTACCATTAGCAAGGCCGGAAACGTCACCA
NS5-60nt-T6	GTTCCAATTGTGAAGATTCTCATAAACAAAATGAAACCATCGATAGCAGCACCGTAATCA
NS5-60nt-T7	CAGCAGCAACGAGCAAAAGGTGTGAGTAAAGTAGCGACAGAATCAAGTTTGCCTTTAGCG
NS5-60nt-T8	AGAAAATAGTTGGCATCATAAAGTAATGGGTCAGACTGTAGCGCGTTTTCATCGGCATTT
NS5-60nt-T9	TGTTCAACACCAGTGTCTGTACTCAATTGATCGGTCATAGCCCCCTTATTAGCGTTTGCC
NS5-60nt-T10	AAGAAAAAGAAGTACGCTATTAACTATTAAATCTTTTCATAATCAAAATCACCGGAACCA
NS5-60nt-T11	TTCCAAACAGAAAAACTAATATAATATTTAGAGCCACCACCGGAACCGCCTCCCTCAGAG
NS5-60nt-T12	TTTCTGGATTGAATGACCACATGGAACGCGCCGCCACCCTCAGAACCGCCACCCTCAGAG
NS5-60nt-T13	GTAGCAACAGTGATTTCTTTAGGCAGGTCCCCACCACCCTCAGAGCCGCCACCAGAACCA
NS5-60nt-T14	TATGAGGTTTATGATGTAATCAAGATTCCACCACCAGAGCCGCCGCCAGCATTGACAGGA
NS5-60nt-T15	TTGTTATCAGCTAGAGGATGAAATGGTGAAGGTTGAGGCAGGTCAGACGATTGGCCTTGA

NS5-60nt-T16	AGCAGAAAGGCTAAAAAGCACAAATAGAAGTATTCACAAACAAATAAATCCTCATTAAAG
NS5-60nt-T17	CGATATCGATGTACTGAATGGGTGATTTAGCCAGAATGGAAAGCGCAGTCTCTGAATTTA
NS5-60nt-T18	AGACGCAGTATTATTGGGTAAACCTTGGGGCCGTTCCAGTAAGCGTCATACATGGCTTTT
NS5-60nt-T19	ATTTTACCGTCACCACCACGAATTCGTCTGGATGATACAGGAGTGTACTGGTAATAAGTT
NS5-60nt-T20	CTACGTGATGAGGAACGAGAAGAGGCTTGATTAACGGGGTCAGTGCCTTGAGTAACAGTG
NS5-60nt-T21	GTCTGCCGAAAGCTTGTGTTACATTGTATGCCCGTATAAACAGTTAATGCCCCCTGCCTA
NS5-60nt-T22	TGGTGGGAATGTTTTGTATGCGTCAATATGTTTCGGAACCTATTATTCTGAAACATGAAA
NS5-60nt-T23	ATATCGTAAACGGAAAAGCGAAAACGTTTAGTATTAAGAGGCTGAGACTCCTCAAGAGAA
NS5-60nt-T24	TAAAATTAATTTTACACATTAGGGCTCTTCGGATTAGGATTAGCGGGGTTTTGCTCAGTA

Table S3 – Tiling oligo sequences

Tiling oligos for 24 target nanoswitches (base set)

Var 1	AACATCCAATAAATCATACAGGCAAGGCAAAGAATTAGCAAAATTAAGCAATAAAGCCTC
1.01	AGAGCATAAAGCTAAATCGGTTGTACCAAAAACATTATGACCCTGTAATACTTTTGCGGG
1.02	AGAAGCCTTTATTTCAACGCAAGGATAAAAATTTTTAGAACCCTCATATATTTTAAATGC
1.03	AATGCCTGAGTAATGTGTAGGTAAAGATTCAAAAGGGTGAGAAAGGCCGGAGACAGTCAA
1.04	ATCACCATCAATATGATATTCAACCGTTCTAGCTGATAAATTAATGCCGGAGAGGGTAGC
1.05	TATTTTTGAGAGATCTACAAAGGCTATCAGGTCATTGCCTGAGAGTCTGGAGCAAACAAG
1.06	AGAATCGATGAACGGTAATCGTAAAACTAGCATGTCAATCATATGTACCCCGGTTGATAA
1.07	TCAGAAAAGCCCCAAAAACAGGAAGATTGTATAAGCAAATATTTAAATTGTAAACGTTAA
1.08	TATTTTGTTAAAATTCGCATTAAATTTTTGTTAAATCAGCTCATTTTTTAACCAATAGGA
1.09	ACGCCATCAAAAATAATTCGCGTCTGGCCTTCCTGTAGCCAGCTTTCATCAACATTAAAT
Var 2	GTGAGCGAGTAACAACCCGTCGGATTCTCCGTGGGAACAAACGGCGGATTGACCGTAATG
2.01	GGATAGGTCACGTTGGTGTAGATGGGCGCATCGTAACCGTGCATCTGCCAGTTTGAGGGG
2.02	ACGACGACAGTATCGGCCTCAGGAAGATCGCACTCCAGCCAGCTTTCCGGCACCGCTTCT
2.03	GGTGCCGGAAACCAGGCAAAGCGCCATTCGCCATTCAGGCTGCGCAACTGTTGGGAAGGG
2.04	CGATCGGTGCGGGCCTCTTCGCTATTACGCCAGCTGGCGAAAGGGGGATGTGCTGCAAGG
2.05	CGATTAAGTTGGGTAACGCCAGGGTTTTCCCAGTCACGACGTTGTAAAACGACGGCCAGT
2.06	GCCAAGCTTGCATGCCTGCAGGTCGACTCTAGAGGATCCCCGGGTACCGAGCTCGAATTC
2.07	GTAATCATGGTCATAGCTGTTTCCTGTGTGAAATTGTTATCCGCTCACAATTCCACACAA
2.08	CATACGAGCCGGAAGCATAAAGTGTAAAGCCTGGGGTGCCTAATGAGTGAGCTAACTCAC
2.09	ATTAATTGCGTTGCGCTCACTGCCCGCTTTCCAGTCGGGAAACCTGTCGTGCCAGCTGCA
2.10	TTAATGAATCGGCCAACGCGCGGGGAGAGGCGGTTTGCGTATTGGGCGCCAGGGTGGTTT
Var 3	TTCTTTTCACCAGTGAGACGGGCAACAGCTGATTGCCCTTCACCGCCTGGCCCTGAGAGA
3.01	GTTGCAGCAAGCGGTCCACGCTGGTTTGCCCCAGCAGGCGAAAATCCTGTTTGATGGTGG
3.02	TTCCGAAATCGGCAAAATCCCTTATAAATCAAAAGAATAGCCCGAGATAGGGTTGAGTGT
3.03	TGTTCCAGTTTGGAACAAGAGTCCACTATTAAAGAACGTGGACTCCAACGTCAAAGGGCG
3.04	AAAAACCGTCTATCAGGGCGATGGCCCACTACGTGAACCATCACCCAAATCAAGTTTTTT
3.05	GGGGTCGAGGTGCCGTAAAGCACTAAATCGGAACCCTAAAGGGAGCCCCCGATTTAGAGC
3.06	TTGACGGGGAAAGCCGGCGAACGTGGCGAGAAAGGAAGGGAAGAAAGCGAAAGGAGCGGG
3.07	CGCTAGGGCGCTGGCAAGTGTAGCGGTCACGCTGCGCGTAACCACCACACCCGCCGCGCT
3.08	TAATGCGCCGCTACAGGGCGCGTACTATGGTTGCTTTGACGAGCACGTATAACGTGCTTT
3.09	CCTCGTTAGAATCAGAGCGGGAGCTAAACAGGAGGCCGATTAAAGGGATTTTAGACAGGA
3.10	ACGGTACGCCAGAATCCTGAGAAGTGTTTTTATAATCAGTGAGGCCACCGAGTAAAAGAG
5.01	CATTATCATTTTGCGGAACAAAGAAACCACCAGAAGGAGCGGAATTATCATCATATTCCT
5.02	GATTATCAGATGATGGCAATTCATCAATATAATCCTGATTGTTTGGATTATACTTCTGAA
5.03	TAATGGAAGGGTTAGAACCTACCATATCAAAATTATTTGCACGTAAAACAGAAATAAAGA
5.04	AATTGCGTAGATTTTCAGGTTTAACGTCAGATGAATATACAGTAACAGTACCTTTTACAT
5.05	CGGGAGAAACAATAACGGATTCGCCTGATTGCTTTGAATACCAAGTTACAAAATCGCGCA
5.06	GAGGCGAATTATTCATTTCAATTACCTGAGCAAAAGAAGATGATGAAACAAACATCAAGA
5.07	AAACAAAATTAATTACATTTAACAATTTCATTTGAATTACCTTTTTTAATGGAAACAGTA
5.08	CATAAATCAATATATGTGAGTGAATAACCTTGCTTCTGTAAATCGTCGCTATTAATTAAT
5.09	TTTCCCTTAGAATCCTTGAAAACATAGCGATAGCTTAGATTAAGACGCTGAGAAGAGTCA
5.10	ATAGTGAATTTATCAAAATCATAGGTCTGAGAGACTACCTTTTTAACCTCCGGCTTAGGT
Var 6	TGGGTTATATAACTATATGTAAATGCTGATGCAAATCCAATCGCAAGACAAAGAACGCGA
6.01	GAAAACTTTTTCAAATATATTTTAGTTAATTTCATCTTCTGACCTAAATTTAATGGTTTG
6.02	AAATACCGACCGTGTGATAAATAAGGCGTTAAATAAGAATAAACACCGGAATCATAATTA
6.03	CTAGAAAAAGCCTGTTTAGTATCATATGCGTTATACAAATTCTTACCAGTATAAAGCCAA
6.04	CGCTCAACAGTAGGGCTTAATTGAGAATCGCCATATTTAACAACGCCAACATGTAATTTA
6.05	GGCAGAGGCATTTTCGAGCCAGTAATAAGAGAATATAAAGTACCGACAAAAGGTAAAGTA
6.06	ATTCTGTCCAGACGACGACAATAAACAACATGTTCAGCTAATGCAGAACGCGCCTGTTTA
6.07	TCAACAATAGATAAGTCCTGAACAAGAAAAATAATATCCCATCCTAATTTACGAGCATGT
6.08	AGAAACCAATCAATAATCGGCTGTCTTTCCTTATCATTCCAAGAACGGGTATTAAACCAA
6.09	GTACCGCACTCATCGAGAACAAGCAAGCCGTTTTTATTTTCATCGTAGGAATCATTACCG
6.10	CGCCCAATAGCAAGCAAATCAGATATAGAAGGCTTATCCGGTATTCTAAGAACGCGAGGC
Var 7	GTTTTAGCGAACCTCCCGACTTGCGGGAGGTTTTGAAGCCTTAAATCAAGATTAGTTGCT
7.01	ATTTTGCACCCAGCTACAATTTTATCCTGAATCTTACCAACGCTAACGAGCGTCTTTCCA
7.02	GAGCCTAATTTGCCAGTTACAAAATAAACAGCCATATTATTTATCCCAATCCAAATAAGA
7.03	AACGATTTTTTGTTTAACGTCAAAAATGAAAATAGCAGCCTTTACAGAGAGAATAACATA
7.04	AAAACAGGGAAGCGCATTAGACGGGAGAATTAACTGAACACCCTGAACAAAGTCAGAGGG
7.05	TAATTGAGCGCTAATATCAGAGAGATAACCCACAAGAATTGAGTTAAGCCCAATAATAAG
7.06	AGCAAGAAACAATGAAATAGCAATAGCTATCTTACCGAAGCCCTTTTTAAGAAAAGTAAG
7.07	CAGATAGCCGAACAAAGTTACCAGAAGGAAACCGAGGAAACGCAATAATAACGGAATACC

7.08	CAAAAGAACTGGCATGATTAAGACTCCTTATTACGCAGTATGTTAGCAAACGTAGAAAAT
7.09	ACATACATAAAGGTGGCAACATATAAAAGAAACGCAAAGACACCACGGAATAAGTTTATT
7.10	TTGTCACAATCAATAGAAAATTCATATGGTTTACCAGCGCCAAAGACAAAAGGGCGACAT
9.01	CCAGGCGGATAAGTGCCGTCGAGAGGGTTGATATAAGTATAGCCCGGAATAGGTGTATCA
9.02	CCGTACTCAGGAGGTTTAGTACCGCCACCCTCAGAACCGCCACCCTCAGAACCGCCACCC
9.03	TCAGAGCCACCACCCTCATTTTCAGGGATAGCAAGCCCAATAGGAACCCATGTACCGTAA
9.04	CACTGAGTTTCGTCACCAGTACAAACTACAACGCCTGTAGCATTCCACAGACAGCCCTCA
9.05	TAGTTAGCGTAACGATCTAAAGTTTTGTCGTCTTTCCAGACGTTAGTAAATGAATTTTCT
9.06	GTATGGGATTTTGCTAAACAACTTTCAACAGTTTCAGCGGAGTGAGAATAGAAAGGAACA
9.07	ACTAAAGGAATTGCGAATAATAATTTTTTCACGTTGAAAATCTCCAAAAAAAAAGGCTCCA
9.08	AAAGGAGCCTTTAATTGTATCGGTTTATCAGCTTGCTTTCGAGGTGAATTTCTTAAACAG
9.09	CTTGATACCGATAGTTGCGCCGACAATGACAACAACCATCGCCCACGCATAACCGATATA
9.10	TTCGGTCGCTGAGGCTTGCAGGGAGTTAAAGGCCGCTTTTGCGGGATCGTCACCCTCAGC
Var 10	AGCGAAAGACAGCATCGGAACGAGGGTAGCAACGGCTACAGAGGCTTTGAGGACTAAAGA
10.01	CTTTTTCATGAGGAAGTTTCCATTAAACGGGTAAAATACGTAATGCCACTACGAAGGCAC
10.02	CAACCTAAAACGAAAGAGGCAAAAGAATACACTAAAACACTCATCTTTGACCCCCAGCGA
10.03	TTATACCAAGCGCGAAACAAAGTACAACGGAGATTTGTATCATCGCCTGATAAATTGTGT
10.04	CGAAATCCGCGACCTGCTCCATGTTACTTAGCCGGAACGAGGCGCAGACGGTCAATCATA
10.05	AGGGAACCGAACTGACCAACTTTGAAAGAGGACAGATGAACGGTGTACAGACCAGGCGCA
10.06	TAGGCTGGCTGACCTTCATCAAGAGTAATCTTGACAAGAACCGGATATTCATTACCCAAA
10.07	TCAACGTAACAAAGCTGCTCATTCAGTGAATAAGGCTTGCCCTGACGAGAAACACCAGAA
10.08	CGAGTAGTAAATTGGGCTTGAGATGGTTTAATTTCAACTTTAATCATTGTGAATTACCTT
10.09	ATGCGATTTTAAGAACTGGCTCATTATACCAGTCAGGACGTTGGGAAGAAAAATCTACGT
10.10	TAATAAAACGAACTAACGGAACAACATTATTACAGGTAGAAAGATTCATCAGTTGAGATT
Var 11	TAGGAATACCACATTCAACTAATGCAGATACATAACGCCAAAAGGAATTACGAGGCATAG
11.01	TAAGAGCAACACTATCATAACCCTCGTTTACCAGACGACGATAAAAACCAAAATAGCGAG
11.02	AGGCTTTTGCAAAAGAAGTTTTGCCAGAGGGGGTAATAGTAAAATGTTTAGACTGGATAG
11.03	CGTCCAATACTGCGGAATCGTCATAAATATTCATTGAATCCCCCTCAAATGCTTTAAACA
11.04	GTTCAGAAAACGAGAATGACCATAAATCAAAAATCAGGTCTTTACCCTGACTATTATAGT
11.05	CAGAAGCAAAGCGGATTGCATCAAAAAGATTAAGAGGAAGCCCGAAAGACTTCAAATATC
11.06	GCGTTTTAATTCGAGCTTCAAAGCGAACCAGACCGGAAGCAAACTCCAACAGGTCAGGAT
11.07	TAGAGAGTACCTTTAATTGCTCCTTTTGATAAGAGGTCATTTTTGCGGATGGCTTAGAGC
11.08	TTAATTGCTGAATATAATGCTGTAGCTCAACATGTTTTAAATATGCAACTAAAGTACGGT
11.09	GTCTGGAAGTTTCATTCCATATAACAGTTGATTCCCAATTCTGCGAACGAGTAGATTTAG
11.10	TTTGACCATTAGATACATTTCGCAAATGGTCAATAACCTGTTTAGCTAT
Var 12	ATTTTCATTTGGGGCGCGAGCTGAAAAGGTGGCATCAATTCTACTAATAGTAGTAGCATT

Additional tiling oligos for 5 target nanoswitch (added to base set)

4.025	TGGAAATACCTACATTTTGACGCTCAATCG
4.03	TCTGAAATGGATTATTTACATTGGCAGATTCACCAGTCACACGACCAGTAATAAAAGGGA
4.04	CATTCTGGCCAACAGAGATAGAACCCTTCTGACCTGAAAGCGTAAGAATACGTGGCACAG
4.05	ACAATATTTTTGAATGGCTATTAGTCTTTAATGCGCGAACTGATAGCCCTAAAACATCGC
4.06	CATTAAAAATACCGAACGAACCACCAGCAGAAGATAAAACAGAGGTGAGGCGGTCAGTAT
4.07	TAACACCGCCTGCAACAGTGCCACGCTGAGAGCCAGCAGCAAATGAAAAATCTAAAGCAT
4.08	CACCTTGCTGAACCTCAAATATCAAACCCTCAATCAATATCTGGTCAGTTGGCAAATCAA
4.09	CAGTTGAAAGGAATTGAGGAAGGTTATCTAAAATATCTTTAGGAGCACTAACAACTAATA
4.1	GATTAGAGCCGTCAATAGATAATACATTTGAGGATTTAGAAGTATTAGACTTTACAAACA
Var5	ATTCGACAACTCGTATTAAATCCTTTGCCCGAACGTTATTAATTTTAAAAGTTTGAGTAA
8.025	ATGAAACCATCGATAGCAGCACCGTAATCA
8.03	GTAGCGACAGAATCAAGTTTGCCTTTAGCGTCAGACTGTAGCGCGTTTTCATCGGCATTT
8.04	TCGGTCATAGCCCCCTTATTAGCGTTTGCCATCTTTTCATAATCAAAATCACCGGAACCA
8.05	GAGCCACCACCGGAACCGCCTCCCTCAGAGCCGCCACCCTCAGAACCGCCACCCTCAGAG
8.06	CCACCACCCTCAGAGCCGCCACCAGAACCACCACCAGAGCCGCCGCCAGCATTGACAGGA
8.07	GGTTGAGGCAGGTCAGACGATTGGCCTTGATATTCACAAACAAATAAATCCTCATTAAAG
8.08	CCAGAATGGAAAGCGCAGTCTCTGAATTTACCGTTCCAGTAAGCGTCATACATGGCTTTT
8.09	GATGATACAGGAGTGTACTGGTAATAAGTTTTAACGGGGTCAGTGCCTTGAGTAACAGTG
8.1	CCCGTATAAACAGTTAATGCCCCCTGCCTATTTCGGAACCTATTATTCTGAAACATGAAA
Var9	GTATTAAGAGGCTGAGACTCCTCAAGAGAAGGATTAGGATTAGCGGGGTTTTGCTCAGTA

Additional tiling oligos for single target nanoswitch (added to base set)

Var4.5	CAATACTTCTTTGATTAGTAATAACATCAC
4.01	TTGCCTGAGTAGAAGAACTCAAACTATCGGCCTTGCTGGTAATATCCAGAACAATATTAC
4.02	CGCCAGCCATTGCAACAGGAAAAACGCTCATGGAAATACCTACATTTTGACGCTCAATCG
4.03	TCTGAAATGGATTATTTACATTGGCAGATTCACCAGTCACACGACCAGTAATAAAAGGGA
4.04	CATTCTGGCCAACAGAGATAGAACCCTTCTGACCTGAAAGCGTAAGAATACGTGGCACAG
4.05	ACAATATTTTTGAATGGCTATTAGTCTTTAATGCGCGAACTGATAGCCCTAAAACATCGC

4.06	CATTAAAAATACCGAACGAACCACCAGCAGAAGATAAAACAGAGGTGAGGCGGTCAGTAT
4.07	TAACACCGCCTGCAACAGTGCCACGCTGAGAGCCAGCAGCAAATGAAAAATCTAAAGCAT
4.08	CACCTTGCTGAACCTCAAATATCAAACCCTCAATCAATATCTGGTCAGTTGGCAAATCAA
4.09	CAGTTGAAAGGAATTGAGGAAGGTTATCTAAAATATCTTTAGGAGCACTAACAACTAATA
4.1	GATTAGAGCCGTCAATAGATAATACATTTGAGGATTTAGAAGTATTAGACTTTACAAACA
Var5	ATTCGACAACTCGTATTAAATCCTTTGCCCGAACGTTATTAATTTTAAAAGTTTGAGTAA
Var8.5	TGACGGAAATTATTCATTAAAGGTGAATTA
8.01	TCACCGTCACCGACTTGAGCCATTTGGGAATTAGAGCCAGCAAAATCACCAGTAGCACCA
8.02	TTACCATTAGCAAGGCCGGAAACGTCACCAATGAAACCATCGATAGCAGCACCGTAATCA
8.03	GTAGCGACAGAATCAAGTTTGCCTTTAGCGTCAGACTGTAGCGCGTTTTCATCGGCATTT
8.04	TCGGTCATAGCCCCCTTATTAGCGTTTGCCATCTTTTCATAATCAAAATCACCGGAACCA
8.05	GAGCCACCACCGGAACCGCCTCCCTCAGAGCCGCCACCCTCAGAACCGCCACCCTCAGAG
8.06	CCACCACCCTCAGAGCCGCCACCAGAACCACCACCAGAGCCGCCGCCAGCATTGACAGGA
8.07	GGTTGAGGCAGGTCAGACGATTGGCCTTGATATTCACAAACAAATAAATCCTCATTAAAG
8.08	CCAGAATGGAAAGCGCAGTCTCTGAATTTACCGTTCCAGTAAGCGTCATACATGGCTTTT
8.09	GATGATACAGGAGTGTACTGGTAATAAGTTTTAACGGGGTCAGTGCCTTGAGTAACAGTG
8.1	CCCGTATAAACAGTTAATGCCCCCTGCCTATTTCGGAACCTATTATTCTGAAACATGAAA
Var9	GTATTAAGAGGCTGAGACTCCTCAAGAGAAGGATTAGGATTAGCGGGGTTTTGCTCAGTA
